# Genetic Sharing with Cardiovascular Disease Risk Factors and Diabetes Reveals Novel Bone Mineral Density Loci

**DOI:** 10.1371/journal.pone.0144531

**Published:** 2015-12-22

**Authors:** Sjur Reppe, Yunpeng Wang, Wesley K. Thompson, Linda K. McEvoy, Andrew J. Schork, Verena Zuber, Marissa LeBlanc, Francesco Bettella, Ian G. Mills, Rahul S. Desikan, Srdjan Djurovic, Kaare M. Gautvik, Anders M. Dale, Ole A. Andreassen, GEFOS Consortium

**Affiliations:** 1 Department of Medical Biochemistry, Oslo University Hospital, Oslo, Norway; 2 Lovisenberg Diakonale Hospital, Oslo, Norway; 3 Institute of Basic Medical Sciences, University of Oslo, Oslo, Norway; 4 NORMENT, KG Jebsen Centre for Psychosis Research, Institute of Clinical Medicine, University of Oslo, Oslo, Norway; 5 Division of Mental Health and Addiction, Oslo University Hospital, Oslo, Norway; 6 Department of Psychiatry, University of California San Diego, La Jolla, California, United States of America; 7 Department of Neurosciences, University of California San Diego, La Jolla, California, United States of America; 8 Multimodal Imaging Laboratory, University of California San Diego, La Jolla, California, United States of America; 9 Department of Radiology, University of California San Diego, La Jolla, California, United States of America; 10 Cognitive Sciences Graduate Program, University of California San Diego, La Jolla, California, United States of America; 11 Department of Clinical Molecular Biology, Institute of Clinical Medicine, University of Oslo, Oslo, Norway; 12 Oslo Centre for Biostatistics and Epidemiology, Department of Biostatistics, University of Oslo, Oslo, Norway; 13 Prostate Cancer Research Group, Centre for Molecular Medicine Norway (NCMM), University of Oslo and Oslo University Hospital, Oslo, Norway; University of Birmingham, UNITED KINGDOM

## Abstract

Bone Mineral Density (BMD) is a highly heritable trait, but genome-wide association studies have identified few genetic risk factors. Epidemiological studies suggest associations between BMD and several traits and diseases, but the nature of the suggestive comorbidity is still unknown. We used a novel genetic pleiotropy-informed conditional False Discovery Rate (FDR) method to identify single nucleotide polymorphisms (SNPs) associated with BMD by leveraging cardiovascular disease (CVD) associated disorders and metabolic traits. By conditioning on SNPs associated with the CVD-related phenotypes, type 1 diabetes, type 2 diabetes, systolic blood pressure, diastolic blood pressure, high density lipoprotein, low density lipoprotein, triglycerides and waist hip ratio, we identified 65 novel independent BMD loci (26 with femoral neck BMD and 47 with lumbar spine BMD) at conditional FDR < 0.01. Many of the loci were confirmed in genetic expression studies. Genes validated at the mRNA levels were characteristic for the osteoblast/osteocyte lineage, Wnt signaling pathway and bone metabolism. The results provide new insight into genetic mechanisms of variability in BMD, and a better understanding of the genetic underpinnings of clinical comorbidity.

## Introduction

Low bone mineral density (BMD) is an important human phenotype predisposing for bone fractures [1]. Primary and secondary osteoporosis, (defined as BMD less than 2.5 SD of young controls) occur frequently in all populations and lead to high risk for fractures and lasting functional impairment, resulting in long term personal suffering and high social costs [2]. Several lines of evidence suggest an overlap between BMD/osteoporosis and several traits related to metabolism and cardiovascular disease (CVD): -presence of osteoporosis is associated with a ~4-fold increase in risk for an acute cardiovascular event [3].—BMD loss is associated with increased mortality from coronary heart disease and pulmonary diseases [4]- an inverse relationship is found between high-density lipoprotein (HDL) cholesterol and BMD [5–9]. The relationship between low-density lipoprotein (LDL) cholesterol and BMD appears to be less profound, but a positive association has been found in some studies [5,10]. While not all studies have identified a relationship between Triglycerides (TG) and BMD, a few larger studies have shown an inverse relationship [7,8,10]. Furthermore, statins are widely used as cholesterol-lowering drugs, and a recent meta-analysis indicates that statins may help improve and maintain BMD at the lumbar spine, hip and femoral neck, especially in Caucasians and Asians [11].

Blood pressure and anthropometric measures have also been found to be associated with BMD in epidemiological studies. Lee et al. [12]. found that both high systolic blood pressure (SBP) and high diastolic blood pressure (DBP) were associated with low femoral BMD, but not with lumbar BMD in a total study sample consisting of 8439 men and postmenopausal women aged 50 years and older. A study of 586 postmenopausal Turkish women also showed a significant correlation between SBP and femur BMD [13]. It should be noted that several studies also failed to find a link between blood pressure and osteoporosis, e.g. [14].

There is also clinical and epidemiological evidence for association between BMD and metabolic traits. As reviewed [15–17], it is well documented that Type 1 Diabetes (T1D) and Type 2 Diabetes (T2D) increase risk of fracture. Also, it is well established that a major part of the increased fracture risk in T1D is caused by reduced BMD, due to defects in osteoblast differentiation and activity as well as contributing factors including accumulation of advanced glycation end products (AGEs)[18]. Thus, it is plausible that the microenvironment in which B cells develop, the bone marrow including osteoblasts, is influenced by genetic factors that affect both an autoimmune disease like T1D and osteoporosis.

The relationship between T2D and BMD or fracture is more complicated, since the effect on bone microstructure appears to be more important. However, Sayers et al. [19] found an inverse association between insulin and both periosteal circumference and cortical BMD in adolescents after adjusting for all body composition variables, indicating that insulin levels and diabetes have effects on bone metabolism. In adults T2D has been associated with high BMD [16,17] and Billings et al. [20] identified Integrin, Alpha 1 (*ITGA1*) as a new locus candidate, capable of influencing both fasting glucose and BMD, thus pointing to a possible explanation for the epidemiological observations linking T2D diabetes and BMD/osteoporosis. The previous concept, that obesity is protective for osteoporosis is weakened since several studies have shown a negative correlation between WHR and BMD [21–23]. Many of the previous studies did not take into consideration that DXA measurements are falsely elevated by increased body fat and that the associated increase in bone marrow adiposity occurs at the expense of bone [23].

The co-morbidity between BMD and CVD risk factors or metabolic traits have been postulated to be, at least partly, caused by overlapping genes (pleiotropy) [24]. GWAS have identified several genes and single nucleotide polymorphisms (SNPs) associated with BMD [25], and CVD risk factors or metabolic traits, including HDL [26], LDL [26], TG [26], T1D [27], T2D [28], SBP [29], DBP [29] and WHR [29]. Despite the strong heritable component of BMD, the genes identified in GWAS so far explain only a small proportion of the variance (‘missing heritability‘) [25]. Due to the polygenic architecture of BMD, a large number of SNPs have associations too weak to be identified in the currently available cohorts. Thus, pleiotropic enrichment together with cost-effective analytical methods may identify a larger proportion of SNPs associated with BMD.

Standard methods to assess genetic pleiotropy have not taken full advantage of the existing GWAS data and the majority of these studies have focused on the subset of SNPs exceeding a Bonferroni-corrected threshold of significance for each trait or disorder [30,31]. However, this Bonferroni–based approach cannot detect SNPs that only reach genome-wide significance in the combined analysis but do not meet significance cutoffs in the individual phenotype. In the current study, we applied a recently developed genetic pleiotropy-informed approach for GWAS to leverage the power of multiple large GWAS of CVD risk factors blood lipids (HDL, LDL, TG), metabolic disorders (T1D, T2D), blood pressure (SBP, DBP), and waist-hip ratio (WHR) to identify susceptibility SNPs, and capture more of the polygenic effects in BMD [32–34]. This novel genetic epidemiological approach is able to take advantage of polygenic pleiotropy among several types of diseases to identify genetic variants with smaller effect sizes, and thus elucidate the mechanism of variability in BMD. We used summary statistics (p-values and allele frequencies) from the analysis data (up to 32,961 individuals) in the primary study of BMD [25] for both femoral neck (FN) and lumbar spine (LS) BMD phenotypes.

## Materials and Methods

### Participant Samples and Statistical Strategy

The study was approved by the Norwegian Regional Ethical Committee (REK no: 2010/2539) and conducted according to the Declaration of Helsinki (2000). Written informed consent was given by participants for their clinical records to be used in this study. We obtained complete stage 1 GWAS results in the form of summary statistics p-values from public access websites or through collaboration with investigators (T1D cases and controls from The Type 1 Diabetes Genetics Consortium, BMD cases and controls from the GEFOS Consortium). There was some overlap among several of the participants in the anthropometric GWAS and the BMD GWAS sample (for further details, see S1 Table).

### Statistical Analyses

#### Overall Approach

After applying genomic inflation control, we compute the stratified empirical cumulative distribution functions (*cdfs*) of the nominal p-values. Strata are determined by relative enrichment of pleiotropic SNPs in BMD as a function of increased nominal p-values in the different associated traits and disorders. Using this stratified methodology, we construct two-dimensional FDR “look-up” tables (S1 and S2 Figs), with FDR in BMD SNPs computed *conditional* on nominal associated phenotypes p-values (conditional FDR). Using this table we identify loci that are significantly associated with BMD at a conditional FDR level of 0.01. All p-values were corrected for inflation using the genomic control procedure [35], and for overlap in samples [36] as previously described [37]. Finally, the SNP gene associations were validated using information from global transcriptional mapping of bone biopsies from postmenopausal women [38,39].

#### Genomic Control

The empirical null distribution in GWAS is affected by global variance inflation due to population stratification and cryptic relatedness and deflation due to over-correction of test statistics for polygenic traits by standard genomic control methods. We used the same formulism as in Schork *et al*. [35]. The genomic inflation factor λ_GC_ for each phenotype were estimated based on intergenic SNPs as the median z-score squared divided by the expected median of a chi-square distribution with one degree of freedom and divided all test statistics by λ_GC_. We have previously reported that intergenic SNPs, as defined in our annotation protocol (Schork et al, 2013) are deplete of association with >30 complex traits/diseases, and it seems that this is a generic feature for SNPs in this category. Furthermore, intergenic SNPs do not show skewed distribution towards small minor allele frequency (MAF) based on the 1000 Genomes Project (1KGP) [32,33,37].

#### Conditional Q-Q Plots for Pleiotropic Enrichment

**Fig 1:**
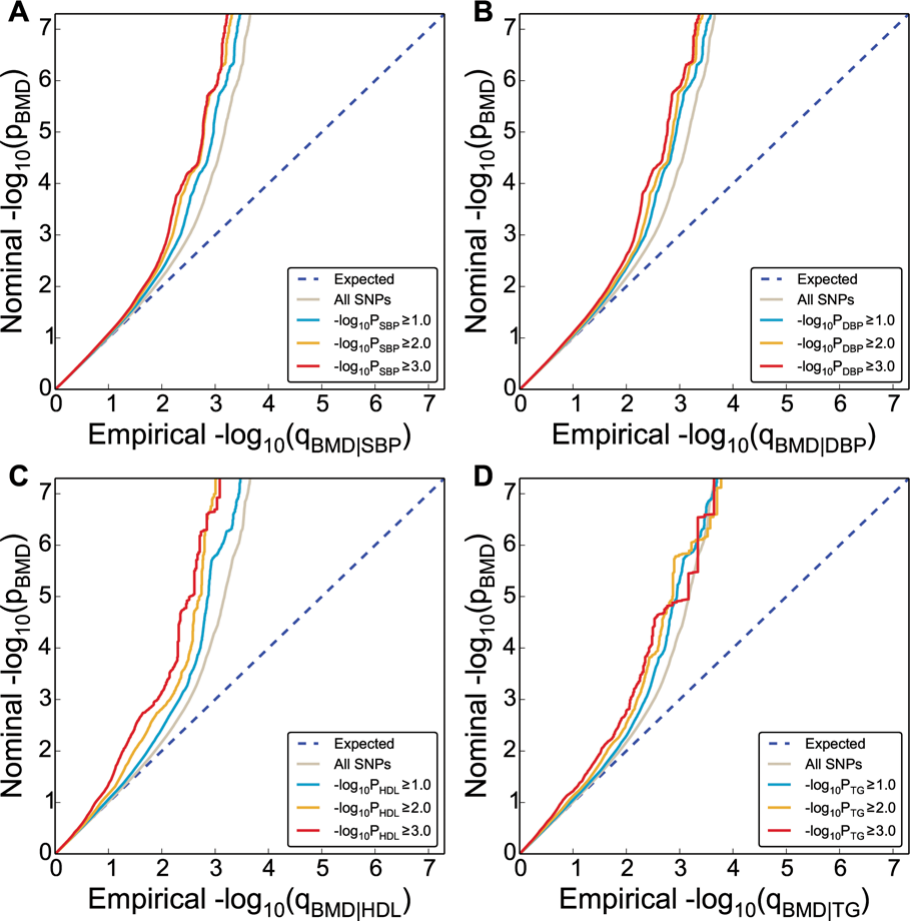
Genetic enrichment. Conditional Q-Q plot of nominal versus empirical -log_10_ p-values (corrected for inflation) in bone mineral density (BMD, femoral neck) below the standard GWAS threshold of p < 5x10^-8^ as a function of significance of association with CVD risk factors, including systolic blood pressure (SBP), diastolic blood pressure (DBP), high density lipoproteins (HDL) and triglycerides (TG) at the level of -log_10_(p) ≥ 0 (all SNPs),–log_10_(p) ≥ 1,–log_10_(p) ≥ 2,–log_10_(p) ≥ 3 corresponding to p ≤ 1, p ≤ 0.1, p ≤ 0.01, p ≤ 0.001, respectively. Dotted lines indicate the null-hypothesis.

To assess pleiotropic enrichment, we used Q-Q plot conditional by ‘pleiotropic’ effects as described in detail earlier (Fig 1) [33,34,37]. For a given associated phenotype, enrichment for pleiotropic signals is present if the degree of deflection from the expected null line is dependent on SNP associations with the second phenotype. Specifically, we computed the empirical cumulative distribution of nominal p-values for a given phenotype for all SNPs and for SNPs with significance levels below the indicated cut-offs for the other phenotype (–log_10_(p) ≥ 0,–log_10_(p) ≥ 1,–log_10_(p) ≥2,–log_10_(p) ≥3 corresponding to p < 1, p < 0.1, p < 0.01, p < 0.001, respectively). The nominal p-values (–log_10_(p)) are plotted on the y-axis, and the empirical quantiles (–log_10_(q), where q = 1-cdf(p)) are plotted on the x-axis. To assess for polygenic effects below the standard GWAS significance threshold, we focused the conditional Q-Q plots on SNPs with nominal–log_10_(p) < 7.3 (corresponding to p > 5x10^-8^).

#### Conditional Statistics–Test of Association with BMD

To improve detection of SNPs associated with BMD, we used a conditional False discovery rate (FDR) approach, leveraging pleiotropic phenotypes [32–34,37]. Specifically, the conditional FDR of a trait (e.g. BMD) for a SNP with p-value *P*_1_ on a second pleiotropic trait with p-value *P*_2_, is computed as the posterior probability that the SNP is null for the first trait given that the p-values for both phenotypes are as small as or smaller than the observed p-values, *FDR*(*P*_1_│*P*_2_) = π_0_(*P*_2_)*P*_1_/*F*(*P*_1_│*P*_2_), where *F*(*P*_1_│*P*_2_)is the conditional *cdf* and π_0_(*P*_2_)the conditional proportional of null SNPs for the first phenotype given that p-value for the second phenotype are *P*_2_ or smaller. The values of *FDR*(*P*_1_│*P*_2_) were conservatively estimated by setting π_0_(*P*_2_) equal one and replacing *F*(*P*_1_│*P*_2_) by empirical conditional *cdf*. The conditional FDR values for BMD on second pleiotropic traits (denoted by *FDR*_*BMD*_, where the dot denotes a second phenotype) were assigned, based on the combination of p-value for the SNP correlated to BMD and the associated trait, by interpolation into a 2-D look-up table (S1 and S2 Figs). All SNPs with FDR < 0.01 (-log_10_(FDR) > 2) in BMD given the different associated phenotypes were identified. A significance threshold of FDR < 0.01 corresponds to 1 false positive per 100 reported associations.

#### Annotation of Novel Loci

Based on 1KGP linkage disequilibrium (LD) structure, significant SNPs identified by conditional FDR were clustered into LD blocks at the LD-r^2^ > 0.2 level. This threshold was chosen since it has been used in a large number of reported GWAS, thus making our result comparable to previous studies, e.g.[25,39,40]. The blocks were numbered as loci # in Table 1 and S2, S3 and S4 Tables and any one block may contain more than one SNPs. Genes close to each SNPs were obtained from NCBI gene database. Blocks that do not contain SNPs or close-by genes to SNPs from primary study were deemed as novel loci in current study (Table 1 and S3 Table). And, loci that contain SNPs or genes from primary study were considered as replication of primary findings (S2 and S4 Tables for FN and LS BMD, respectively). The same procedure was applied to both FN BMD and LS BMD phenotypes. To identify non-overlapping loci between FN BMD and LS BMD, the SNP rs-numbers and gene symbols for these two phenotypes were compared. Loci containing SNPs with same rs-number or same genes were considered overlapping.

**Table 1:** Novel femoral neck BMD associated genes at conditional FDR <0.01.

									Expressed QTL (Age and BMI adj.)	
Loci #	SNP	Gene symbol	Map Loc.	BMD pvalue	BMD FDR	Min cond FDR	Waldstats	Drivingphenotype	Affymetrix ID	r
1	rs10779702	*RERE*	1p36.23	7,78E-08	**3,06E-04**	1,60E-04	-5.26	HDL	200940_s_at	**-0.23**
6	rs12137389	*TESK2*	1p32	1,88E-06	**4,15E-03**	4,01E-03	4.67	HDL	206758_at	-0.11
9	rs11809524	*COL11A1*	1p21	8,21E-07	**2,03E-03**	1,34E-03	-4.83	SBP	37892_at	**0.25**
11	rs9309664	*PPP1CB*	2p23	7,55E-06	1,20E-02	8,22E-03	4.39	HDL	228222_at	**-0.30**
15	rs11675051	*TMEM194B*	2q32.2	1,46E-06	**3,47E-03**	1,56E-03	-4.72	SBP	238014_at	0.09
15	rs13005335	*NAB1*	2q32.3-q33	1,54E-06	**3,47E-03**	1,56E-03	-4.71	SBP	209272_at	0.05
16	rs12995369	*CDK15*	2q33.2	1,07E-07	**3,69E-04**	2,80E-04	-5.2	SBP	1552559_a_at	0.16
17	rs7594560	*METTL21A*	2q33.3	3,42E-06	**5,91E-03**	3,74E-03	4.55	HDL	1553743_at	-0.11
23	rs4957742	*RAB9BP1*	5q21.2	2,98E-06	**5,91E-03**	6,27E-03	-4.58	DBP	NA	NA
27	rs6583337	*FAM20C*	7p22.3	3,30E-06	**5,91E-03**	3,38E-03	4.56	LDL	229438_at	0.18
29	rs2282930	*GRB10*	7p12.2	5,20E-06	**8,40E-03**	7,20E-03	4.46	TG	210999_s_at	**-0.35**
32	rs10953178	*C7orf76*	7q21.3	3,75E-11	**6,36E-07**	3,53E-07	-6.48	HDL	NA	NA
32	rs10464592	*SHFM1*	7q21.3	4,28E-10	**2,35E-06**	4,07E-06	6.11	SBP	202276_at	-0.05
35	rs1670346	*PTPRN2/MIR595*	7q36	1,73E-06	**3,47E-03**	1,80E-03	-4.68	SBP	203030_s_at	0.16
37	rs980299	*EYA1*	8q13.3	1,18E-07	**4,45E-04**	3,39E-04	5.19	HDL	214608_s_at	-0.03
38	rs13272568	*PKIA*	8q21.11	1,29E-06	**2,90E-03**	2,52E-03	4.74	SBP	204612_at	**-0.35**
40	rs665556	*KLF4*	9q31	6,68E-06	1,00E-02	5,84E-03	4.41	DBP	220266_s_at	**-0.34**
49	rs600231	*MALAT1*	11q13.1	7,75E-06	1,20E-02	7,60E-03	-4.38	SBP	231735_s_at	**0.29**
51	rs258415	*KLHL42*	12p11.22	3,55E-08	**1,69E-04**	1,43E-04	-5.4	SBP	NA	NA
53	rs11614913	*MIR196A2*	12q13.13	4,20E-08	**1,69E-04**	1,25E-04	5.37	SBP	NA	NA
54	rs10746070	*RIC8B*	12q23.3	2,14E-06	**4,15E-03**	3,13E-03	-4.64	HDL	229637_at	0.04
58	rs7175531	*CYP19A1*	15q21	2,30E-06	**4,96E-03**	4,82E-03	-4.63	HDL	240705_at	**0.25**
58	rs10851498	*MIR4713*	15q21	2,73E-06	**4,96E-03**	4,45E-03	-4.59	TG	NA	NA
61	rs3198697	*PDXDC1*	16p13.11	1,01E-05	1,44E-02	5,00E-03	4.32	HDL	212053_at	0.08
67	rs199529	*NSF*	17q21	2,39E-06	**4,96E-03**	2,55E-03	4.62	SBP	202395_at	-0.13
71	rs8090312	*NFATC1*	18q23	4,54E-06	**8,40E-03**	6,40E-03	-4.49	T1D	211105_s_at	-0.15
74	rs756632	*RTDR1/GNAZ*	22q11.2	3,39E-06	**5,91E-03**	4,75E-03	-4.55	HDL	220105_at	-0.01
74	rs4820539	*RAB36*	22q11.2	3,06E-06	**5,91E-03**	7,44E-03	4.57	HDL	211471_s_at	0.14

Independent complex or single gene loci (LD-r^2^ < 0.2) with SNP(s) with a conditional FDR (condFDR) < 0.01 in bone mineral density (BMD, Femoral neck) given the association in other phenotype. We defined the most significant BMD SNP in each LD block based on the minimum condFDR (min condFDR) for each phenotype. The most significant SNPs in each gene of the LD block are listed and the second phenotype which provides the minimal FDR signal (Driving phenotype). All loci with SNPs with condFDR < 0.01 were used to define the number of the loci. The following abbreviations were used: Type 1 diabetes (T1D), type 2 diabetes (T2D), systolic blood pressure (SBP), diastolic blood pressure (DBP), low-density lipoproteins (LDL) cholesterol and high-density lipoproteins (HDL) cholesterol, chromosome location (Map Loc.). BMD FDR values < 0.01 are in bold. Bold r values represent nominally significant (p<0.05) Pearson correlations. Gene titles and ontology function terms are presented in S5 Table. Wald stats: z-score transformed from p values NA: not applicable (undetected)

#### Conditional FDR Manhattan Plots

**Fig 2:**
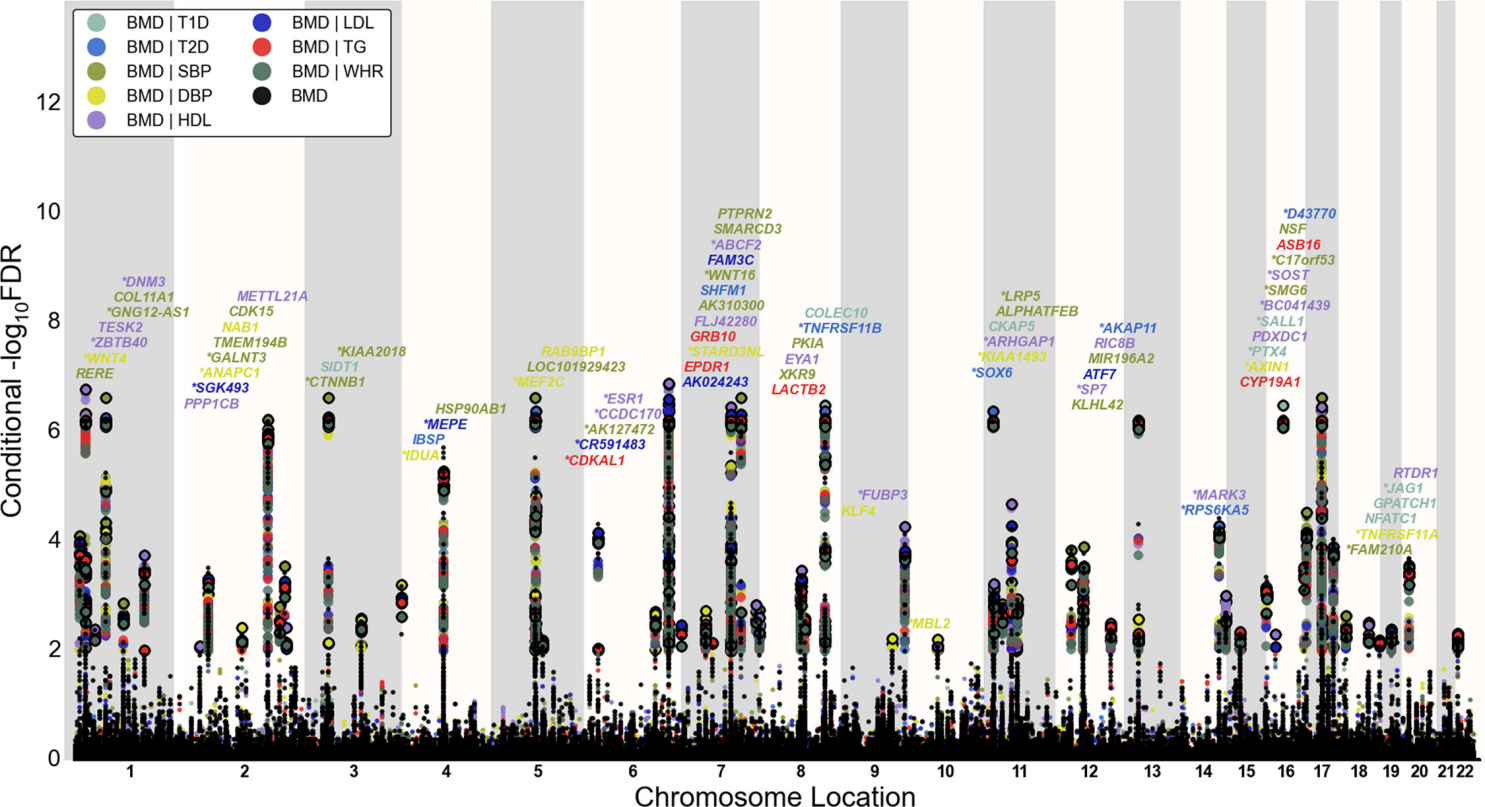
‘Conditional FDR Manhattan plot’ of conditional–log_10_ (FDR) values for bone mineral density (BMD, femoral neck) alone (small black dots) and BMD given the associated phenotypes type 1 diabetes (T1D; BMD|T1D), type 2 diabetes (T2D; BMD|T2D), waist hip ratio (WHR, BMD|WHR), systolic blood pressure (SBP, BMD|SBP), diastolic blood pressure (DBP, BMD|DBP), high density lipoproteins (HDL, BMD|HDL), low density lipoproteins (LDL, BMD|LDL) and triglycerides (TG, BMD|TG). SNPs with conditional–log_10_ FDR > 2 (i.e. FDR < 0.01) are shown with large points. A black line around the large points indicates the most significant SNP in each LD block and this SNP was annotated with the closest gene which is listed above the symbols in each locus, except for the HLA region on chromosome 6. Gene symbols were obtained from NCBI gene databases and colored in line with the second phenotype which gives the minimal conditional FDR value. Details for the novel loci with–log_10_ FDR > 2 (i.e. FDR < 0.01) are shown in Table 1 and S1 Table. Genes previously reported by other studies were marked by stars (*).

To illustrate the localization of the genetic markers associated with BMD given the CVD risk factor effect, we used a ‘Conditional FDR Manhattan plot’, plotting all SNPs within an LD block in relation to their chromosomal location. As illustrated in Fig 2 and S3 Fig, the large points represent the SNPs with FDR < 0.01, whereas the small points represent the non-significant SNPs. All SNPs without ‘pruning’ (removing all SNPs with LD-r^2^ > 0.2 based on 1KGP LD structure) are shown. The strongest signal in each LD block is marked by larger points with black edges. This was identified by ranking all SNPs in increasing order, based on the conditional FDR value for BMD, and then removing SNPs in LD-r^2^ > 0.2 with any higher ranked SNP. Thus, the selected locus was the most significantly associated with BMD in each LD block (Fig 2 and S3 Fig).

#### Validation by Expression Genetics

We looked for expressional association between the SNP associated genes and BMD in bone biopsies from postmenopausal women (n = 84) [38,39]. The Iliac biopsies were analyzed with Affymetrix microchips and log_2_ transformed signal values were correlated to BMD levels (Table 1, S2 Table). The primary data have been submitted to the European Bioinformatics Institute (EMBL-EBI; ID: E-MEXP-1618).

## Results

### Pleiotropic Enrichment-Polygenic Overlap

Conditional Q-Q plots for FN BMD conditioned on nominal p-values of association with T1D, T2D, SBP, DBP, HDL, LDL, TG and WHR showed enrichment across different levels of significance (Fig 1 and S5 Fig). Similar plots for LS BMD are shown in S4 Fig. The earlier departure from the null line (leftward shift) suggests a greater proportion of true associations for a given nominal FN BMD p-value (See S1 File for detailed explanation). Successive leftward shifts for decreasing nominal p-values of a second phenotype indicate that the proportion of non-null effects varies considerably across different levels of association with the comorbidity trait or disease.

### Loci Associated with BMD

To identify SNPs associated with FN BMD, we constructed a “conditional FDR” Manhattan plot showing the FDR conditional on each of the risk factors (Fig 2). We identified significant loci associated with FN BMD leveraging the reduced FDR obtained by the associated phenotype. To estimate the number of independent loci, we pruned the associated SNPs (removed SNP with LD-r^2^ > 0.2), and identified a total of 74 independent loci with a conditional FDR < 0.01 of which 15 were complex loci and 59 single gene loci (marked in Fig 2 by points with black edges). The 74 loci encompassed 84 different genes. Using the FDR method in FN BMD alone, 70 loci were identified (bold values in the “BMD-FDR” column, Table 1 and S2 Table). The remaining 4 loci would not have been identified in the current sample without using the conditional FDR method. Similarly, the 95 independent loci for LS BMD encompasses 107 different genes, and the extra number of loci identified with our conditional FDR compared with FDR method is 21 (bold value in the “BMD-FDR” column, S3 and S4 Tables, marked by points with black edges in S3 Fig). Since there are overlaps in loci between the two phenotypes, we identified a total of 122 independent loci for FN BMD and/or LS BMD, representing 155 different genes in all.

### SNP Detection and Verification

The previous study of BMD related SNPs by Estrada *et al*. [25] identified a total of 56 loci associated with FN BMD and/or LS BMD (49 loci with FN BMD and 48 loci with LS BMD). This was based on two-stage analysis (consisting up to 83,894 and 77,508 individuals), whereas in the stage-1 sample analysis (consisting 32,961 and 31,800 individuals), 20 and 26 loci were associated with FN BMD and LS BMD, respectively. Our analysis re-identified all (20 FN and 26 LS) loci reported in the primary study stage-1 analysis by Estrada et al. [25]. Also in the cross stage (I and II) analyses, all but 5 loci for FN BMD and 8 loci for LS were successfully re-identified (S2 and S4 Tables).

The FDR method identified 26 novel loci associated with FN BMD and 47 novel loci associated with LS BMD, not reported in the previous BMD GWAS [25].

### Gene Expression Analysis

Global gene expression profiling in iliac bone biopsies from 84 postmenopausal women [38] permitted us to calculate the correlation values between BMD and the mRNA levels of all genes associated with the identified loci, as shown in the rightmost columns of Table 1 (novel genes) and S2 Table (genes identified also by Estrada *et al*. [25]). We found a similar fraction of transcripts that were significantly correlated with FN BMD among the novel BMD associated genes (8 out of 26 reaching detection level), very similar to the Estrada study [25], 31% vs. 30%, respectively.

### Functional Enrichment Analysis

The 155 genes encompassed by all loci at FDR < 0.01 for FN and LS BMD were analyzed with Ingenuity Pathway Analysis (IPA). The top-most significantly affected canonical pathway was “Role of Osteoblasts and Chondrocyte in Rheumatoid Arthritis” (p = 4.1x10^-12^), which includes Wnt signaling, and the function and interaction of many of the identified genes in bone related cells (Table 2).

**Table 2:** Top Canonical pathways and Top diseases and Bio Functions from Ingenuity Pathway Analysis

**Canonical Pathways**	**Ratio(p-value)**		**Molecules**
Role of Osteoblasts, Osteoclasts and Chondrocytes in Rheumatoid Arthritis	16/231 (4.10E-12)		SFRP4,RELA,LRP5,TNFSF11,SPP1,AXIN1,WNT2B,WNT16,SP7, TNFRSF11A, NFATC1, WNT4, BMP7, SOST, CTNNB1, TNFRSF11B
Role of Macrophages, Fibroblasts and Endothelial Cells in Rheumatoid Arthritis	12/329 (1.86E-6)		SFRP4, RELA, TNFSF11, LRP5, AXIN1, WNT2B, WNT16, WNT4, SOST,CTNNB1, NFATC1, TNFRSF11B
Wnt/Î^2^-catenin Signaling	9/174 (3.69E-6)		SFRP4, LRP5, SOX6, AXIN1, WNT2B, WNT16, WNT4, SOX9, CTNNB1
Basal Cell Carcinoma Signaling	6/75 (1.32E-5)		AXIN1, WNT2B, WNT16, WNT4, BMP7, CTNNB1
Role of NANOG in Mammalian Embryonic Stem Cell Pluripotency	7/117 (1.52E-5)		AXIN1, WNT2B, WNT16, WNT4, BMP7, CTNNB1, ZFP42
Human Embryonic Stem Cell Pluripotency	7/153 (5.06E-5)		AXIN1, SMAD3, WNT2B, WNT16, WNT4, BMP7, CTNNB1
Colorectal Cancer Metastasis Signaling	9/254 (5.87E-5)		RELA, LRP5, AXIN1, SMAD3, WNT2B, ADCY6, WNT16, WNT4,CTNNB1
Protein Kinase A Signaling	11/389 (9.02E-5)		DHH, RELA, PTPRD, SMAD3, ADCY6, PPP1CB, CTNNB1, EYA1,ANAPC1, NFATC1, AKAP11
Role of Wnt/GSK-3Î^2^ Signaling in the Pathogenesis of Influenza	5/82 (2.48E-4)		AXIN1, WNT2B, WNT16, WNT4, CTNNB1
Regulation of the Epithelial-Mesenchymal Transition Pathway	7/190 (3.13E-4)		RELA, AXIN1, SMAD3, WNT2B, WNT16, WNT4, JAG1
**Categories in Top Diseases and Bio Functions**	**Diseases orFunctionsAnnotation**	**# Molecules(p-Value)**	**Molecules**
Connective Tissue Development and Function, Embryonic Development, Organ Development, Organ Morphology, Organismal Development, Skeletal and Muscular System Development and Function, Tissue Development	abnormalmorphology ofbone	27 (1,86E-15)	ARHGAP1, BMP7, CYP19A1, ESR1, EYA1, FAM20C, GALNT3,HOXC4, HOXC5, HOXC6, IBSP, IDUA, LRP5, MEOX1, MEPE,NAB1, PKDCC, SALL1, SMAD3, SOST, SOX6, SOX9, SPP1,TNFRSF11A, TNFRSF11B, TNFSF11, ULK4
Organismal Development, Skeletal and Muscular System Development and Function	abnormalmorphology of limb	18 (2,48E-13)	BMP7, ESR1, EYA1, FAM20C, GALNT3, IBSP, IDUA, LRP4,LRP5, PKDCC, SALL1, SMAD3, SOST, SOX9, TNFRSF11A,TNFRSF11B, TNFSF11, WNT4
Skeletal and Muscular System Development and Function	abnormalmorphology ofskeleton	19 (1,16E-12)	ARHGAP1, BMP7, ESR1, EYA1, FAM20C, GALNT3, HOXC4,HOXC5, HOXC6, IBSP, IDUA, LRP5, MEOX1, PKDCC, SMAD3,SOST, SOX9, TNFRSF11B, TNFSF11
Connective Tissue Development and Function, Skeletal and Muscular System Development and Function	bone mineraldensity	15 (4,51E-12)	ARHGAP1, CYP19A1, ERCC1, ESR1, FAM20C, GALNT3, IBSP,LRP5, NAB1, SMAD3, SOST, SPP1, TNFRSF11A, TNFRSF11B,TNFSF11
Connective Tissue Development and Function, Embryonic Development, Organ Development, Organ Morphology, Organismal Development, Skeletal and Muscular System Development and Function, Tissue Development	morphology oflimb bone	13 (1,36E-11)	BMP7, ESR1, FAM20C, GALNT3, IBSP, IDUA, LRP5, PKDCC,SMAD3, SOST, SOX9, TNFRSF11B, TNFSF11
Connective Tissue Development and Function, Embryonic Development, Organ Development, Organismal Development, Skeletal and Muscular System Development and Function, Tissue Development	mineralization ofbone	13 (4,78E-11)	BMP7, ESR1, FAM20C, IBSP, LRP5, MEPE, PK DCC, SMAD3,SOST, SOX9, SPP1, TNFRSF11B, WNT4
Skeletal and Muscular System Development and Function	abnormalmorphology ofappendicularskeleton	13 (5,66E-11)	BMP7, ESR1, FAM20C, GALNT3, IBSP, IDUA, LRP5, PKDCC,SMAD3, SOST, SOX9, TNFRSF11B, TNFSF11
Cellular Development	differentiation ofconnective tissuecells	23 (1,94E-10)	AREG/AREGB, AXIN1, BMP7, CTNNB1, FAM20C, JAG1, KLF4,LGR4, LRP5, MEF2C, NFATC1, PKDCC, RELA, SFRP4, SMAD3,SOST, SOX9, SP7, SPP1, TNFRSF11A, TNFRSF11B, TNFSF11,WNT4
Organismal Injury and Abnormalities	calcinosis	9 (3,26E-10)	BMP7, CTNNB1, GALNT3, IBSP, LRP5, SOX9, SPP1,TNFRSF11B, TNFSF11
Cardiovascular Disease	degenerativemitral valvedisease	5 (3,48E-10)	CTNNB1, IBSP, LRP5, SOX9, SPP1

The genes associated with all identified loci (min Cond FDR < 0.01) were subjected to Ingenuity Pathway Analysis. The topmost significantly affected canonical pathways (upper panel) and Categories in Top Diseases and Bio Functions (lower panel) from the analysis are shown.

**Fig 3:**
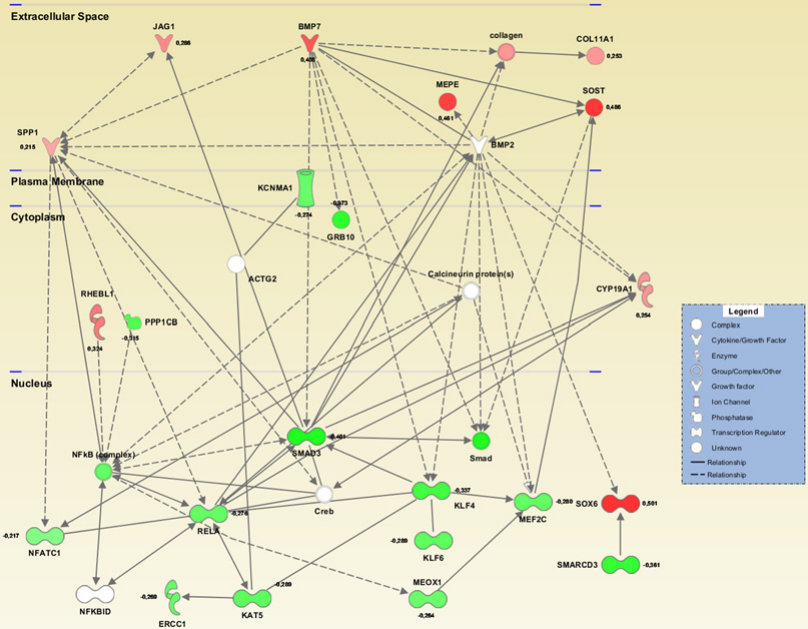
Network analysis IPA-generated network illustrating molecular interactions among the genes correlating inversely (green) or positively (red) to FN or LS BMD.

Out of all the loci at FDR < 0.01 (LS and FN BMD), 48 associated gene transcripts were significantly correlated to BMD in bone biopsies from postmenopausal women. This subset of genes was also analyzed by IPA, and a network of interacting genes including *NFATC1*, *RELA*, *NFΚB* and *SMAD3* as central nuclear hubs were generated (Fig 3).

All genes associated with FN or LS SNPs were analyzed for over-representation in KEGG pathways using Gene Codis (http://genecodis2.dacya.ucm.es/). “Wnt signaling pathway” ranked 1^st^ with 9 genes and corrected chi square p = 8.4x10^-21^. Other highly ranked pathways included “Hedgehog signaling pathway”, “Osteoclast differentiation”, “Focal adhesion” and “Endocrine and other factor-regulated calcium reabsorption”. Interestingly, the pathway “Vascular Smooth Muscle Contraction” also emerged as significant (corrected chi square p = 3.9x10^-3^).

## Discussion

The current analyses of combined GWAS data from more than 250,000 individuals demonstrated genetic overlap between BMD and associated CVD risk factor phenotypes. This indicated that some of the co-morbidity observed in epidemiological and clinical studies may be due to shared risk gene variants. Based on the polygenic enrichment we identified 65 novel BMD loci (26 for FN BMD and 47 for LS BMD) not previously reported. Many of these loci are associated with genes that were validated in our expression assay. The high confirmation rate of the current FDR approach and the association to gene expression assay suggest these loci for follow-up analysis.

By comparing GWAS and gene expression profiling of bone, we can suggest which transcriptional regulators drive the expression of the suggested genes identified in this study. Bone remodeling continues throughout life and involves the fine balance between bone building osteoblasts and resorbing osteoclasts. The complexes NFATc1 and NFkB (including p65/RelA) can function as heterodimers and DNA binding transcriptional activators [41] and are central to osteoclast development and differentiation. They do, however, also have an important function in osteoblasts. Strontium ranelate was shown to increase *NFATc1* transactivation in osteoblasts promoting increased expression of *WNT3A* and *WNT5A* as well as beta-catenin transcription in osteoblasts [42,43]. This positions NFATc1 activation upstream of canonical and non-canonical Wnt signaling pathways, networks whose interactions and strong associations to bone and metabolism are clearly underscored in the present work. NFATc1 activation is also pathogenetically associated with blood pressure via binding to promoter elements on endothelin-1 (*ET-1*) thereby regulating its expression [44]. ET-1 regulates salt excretion in the kidney collecting duct [45]. Through regulation of salt excretion, NFATc1 also has a role in mineral metabolism, and thus possibly also affecting the body’s Ca^++^ balance and metabolism. NFATc1 blockade has been shown to completely prevent oxidized LDL-induced osteogenic transformation of human coronary artery smooth muscle cells as well as oxidized LDL-induced stimulation of osteoblast differentiation [46]. NFATc1 may therefore be a master regulator contributing to predisposition in several of these conditions. Interestingly, the application of this approach has uncovered a uniquely rich and coherent gene network which fully reflects the biological relationship between NFATc1 and the Wnt signaling pathways governing osteoclast/osteoblast activity and engagement in metabolism. Future work should focus on the identification of surrogate markers (transcripts and proteins) of aberrant NFATc1 activation, which in combination with genotyping could provide more accurate risk predictors for the range of conditions affected by this important transcription factor. Vascular smooth muscle contraction was identified as significantly affected among the BMD associated genes. This process is relevant to bone because the contractile elements used in muscle are also a characteristic feature of the osteocytes which constitute 90–95% of bone cells [47], and are dynamic star shaped cells with stretching and contracting protrusions [48]. It is not known if the mechanisms for osteocyte motility are more characteristic to smooth or striated muscle. However, both smooth and striated muscle share common features with osteocytes[49], and muscle-related gene expression in bone has been shown to be affected in postmenopausal osteoporotic women [39] as well as in human iliac bone with reduced BMD due to primary hyperparathyroidism [50].

T1D and T2D are complex metabolic disorders with multiple possible interactions with BMD. However, our results are only to a minor degree influenced by these disorders, since only one of the 26 novel FN BMD associated SNPs has diabetes (T1D) as the driving phenotype (Table 1 and Fig 2) and only 9 (~10%) of the novel the LS BMD associated SNPs has T1D or T2D as the driving phenotype (S3 Table and S3 Fig).

Our results confirm the feasibility of using a genetic epidemiology framework that leverages overlap in genetic signal from independent GWAS of correlated phenotypes for revealing genetic basis of complex phenotypes/diseases. The increased power provided by additional GWAS of associated phenotypes together with the FDR method, roughly doubled the previous number of BMD associated loci [25]. Using the same methods for functional validation of the current findings obtained with our statistical approach, we report a similar rate of significantly expressed genes as in the original BMD report [25]. Furthermore, “Role of Osteoblasts and Chondrocyte in Rheumatoid Arthritis” was the top-most significantly affected canonical pathway when subjecting the 155 genes encompassed by all loci at FDR < 0.01 for FN and LS BMD to IPA. This pathway was also among the most significantly affected in a study by Gupta et al.[51], using a Bayesian block-clustering algorithm to analyze GWAS of multiple phenotypes related to bone, thus supporting our results. It should be noted that, when analyzing BMD associated genes by IPA and similar methods, intergenic, and also intragenic SNPs, not necessarily affects transcription of the closest gene. Gene polymorphisms have been shown to affect more distant genes located several Mbp away [52,53]. More detailed experimental validation of the current findings is warranted. Our method for correction of the overlap in some of the GWAS cohorts examined, should exclude contribution from environmental factors. We also controlled for inflation using genomic control correction of each primary single phenotype GWAS. Further, the overlapping loci were spread over all autosomes in the different phenotypes. If a single control group used in several samples were driving the findings, it would be expected that the same region would have been significant across different phenotypes. This is particularly evident in the GWAS of blood lipids, where the same sample was used to discover new genes for three different phenotypes [26], but the pattern of loci was quite different across the different traits. This suggests that the findings are not due to common genetic variation in potentially overlapping control groups.

In conclusion, we identified 26 and 47 novel genomic loci associated with BMD in FN and LS, respectively, by leveraging genetic pleiotropy with several CVD-related traits, including T1D, T2D, SBP, DBP, LDL, TG, WHR and HDL. Association analyses point to genes involved in metabolism and activated immunological pathways. The results warrant further experimental investigations to clarify the clinical implications, and could lead to improved screening programs and prevention strategies.

## Supporting Information

S1 Fig Conditional FDR 2-D lookup table for femoral neck BMD. Based on the combination of p-value for the SNPs in femoral neck BMD (P_BMD_) and that of the pleiotropic trait: A. type 1 diabetes (T1D), B. type 2 diabetes (T2D), C. systolic blood pressure (SBP), D. diastolic blood pressure (DBP), E. high density lipoprotein (HDL), F. low density lipoprotein (LDL), G. triglycerides (TG), and H. waist hip ratio (WHR) we assigned a conditional FDR value to each SNP associated with femoral neck BMD, by interpolation into a 2-D look-up table. Color scale refers to the conditional FDR values.
(TIF)

S2 Fig Conditional FDR 2-D lookup table for Lumbar Spine BMD. Based on the combination of p-value for the SNPs in lumbar spine BMD (P_BMD_) and that of the pleiotropic trait: A. type 1 diabetes (T1D), B. type 2 diabetes (T2D), C. systolic blood pressure (SBP), D. diastolic blood pressure (DBP), E. high density lipoprotein (HDL), F. low density lipoprotein (LDL), G. triglycerides (TG), and H. waist hip ratio (WHR), we assigned a conditional FDR value to each SNP associated with lumbar spine BMD, by interpolation into a 2-D look-up table. Color scale refers to the conditional FDR values.
(TIF)

S3 Fig Conditional FDR Manhattan plots for lumbar spine BMD. ‘Conditional Manhattan plot’ of conditional–log10 (FDR) values for bone mineral density (BMD, lumbar spine) alone (small black dots) and BMD given the associated phenotypes type 1 diabetes (T1D; BMD|T1D), type 2 diabetes (T2D; BMD|T2D), systolic blood pressure (SBP; BMD|SBP), diastolic blood pressure (DBP; BMD|DBP), high density lipoprotein (HDL; BMD|HDL), low density lipoprotein (LDL; BMD|LDL), triglycerides (TG; BMD|TG), and waist hip ratio (WHR; BMD|WHR). SNPs with conditional–log10 FDR > 2 (i.e. FDR < 0.01) are shown with large points. A black line around the large points indicates the most significant SNP in each LD block and this SNP was annotated with the closest gene, which is listed above the symbols in each locus. Gene symbols were obtained from HGNC gene databases and colored in line with the second phenotype, which gives the minimal conditional FDR value. Genes previously reported by other studies were marked by stars (*).
(TIF)

S4 Fig Genetic pleiotropy enrichment. Conditional Q-Q plot of nominal versus empirical -log10 p-values (corrected for inflation) in bone mineral density (BMD, femoral neck) below the standard GWAS threshold of p < 5x10-8 as a function of significance of association with CVD risk factors, including type 1 diabetes (T1D), type 2 diabetes (T2D), low density lipoprotein (LDL) and waist hip ratio (WHR) at the level of -log10(p) ≥ 0 (all SNPs),–log10(p) ≥ 1,–log10(p) ≥ 2,–log10(p) ≥ 3 corresponding to p ≤ 1, p ≤ 0.1, p ≤ 0.01, p ≤ 0.001, respectively. Dotted lines indicate the null-hypothesis.
(TIF)

S5 Fig QQ plots for Lumbar Spine-BMD. Conditional Q-Q plot of nominal versus empirical -log_10_ p-values (corrected for inflation) in bone mineral density (BMD, lumbar spine) below the standard GWAS threshold of p < 5x10^-8^ as a function of significance of association with A. type 1 diabetes (T1D), B. type 2 diabetes (T2D), C. systolic blood pressure (SBP), D. diastolic blood pressure (DBP), E. high density lipoprotein (HDL), F. low density lipoprotein (LDL), G. triglycerides (TG), and H. waist hip ratio (WHR) at the level of -log_10_(p) ≥ 0 (all SNPs),–log_10_(p) ≥ 1,–log_10_(p) ≥ 2,–log_10_(p) ≥ 3 corresponding to p ≤ 1, p ≤ 0.1, p ≤ 0.01, p ≤ 0.001, respectively. Dotted lines indicate the null-hypothesis.
(TIF)

S6 Fig Conditional QQ plot for Femoral neck BMD on CAD. Conditional Q-Q plot of nominal versus empirical -log_10_ p-values (corrected for inflation) in bone mineral density (BMD, femoral neck) below the standard GWAS threshold of p < 5x10^-8^ as a function of significance of association with Coronary Artery Disease (CAD) at the level of -log_10_(p) ≥ 0 (all SNPs),–log_10_(p) ≥ 1,–log_10_(p) ≥ 2,–log_10_(p) ≥ 3 corresponding to p ≤ 1, p ≤ 0.1, p ≤ 0.01, p ≤ 0.001, respectively. Dotted lines indicate the null-hypothesis.
(TIF)

S7 Fig Conditional QQ plot for lumbar spine BMD on CAD. Conditional Q-Q plot of nominal versus empirical -log_10_ p-values (corrected for inflation) in bone mineral density (BMD, lumbar spine) below the standard GWAS threshold of p < 5x10^-8^ as a function of significance of association with Coronary Artery Disease (CAD) at the level of -log_10_(p) ≥ 0 (all SNPs),–log_10_(p) ≥ 1,–log_10_(p) ≥ 2,–log_10_(p) ≥ 3 corresponding to p ≤ 1, p ≤ 0.1, p ≤ 0.01, p ≤ 0.001, respectively. Dotted lines indicate the null-hypothesis.
(TIF)

S1 File Details of Statistical Analysis (DOC)

S1 Table Summary data from all GWAS used in the current study (DOCX)

S2 Table All identified loci associated with femoral neck BMD (DOCX)

S3 Table Identified loci containing novel SNPs or genes associated with lumbar spine BMD (DOCX)

S4 Table Identified loci containing known SNPs or genes associated with lumbar spine BMD (DOCX)

S5 Table Gene titles and gene ontology function terms of genes associated with LS an FN BMD loci at FDR <0.01 (DOCX)

## References

[1] Kanis JA, Oden A, Johnell O, Johansson H, De Laet C, Brown J, et al (2007) The use of clinical risk factors enhances the performance of BMD in the prediction of hip and osteoporotic fractures in men and women. Osteoporos Int 18: 1033–1046. 17323110 10.1007/s00198-007-0343-y

[2] Compston J (2010) Osteoporosis: social and economic impact. Radiol Clin North Am 48: 477–482. S0033-8389(10)00011-4 [pii]; 10.1016/j.rcl.2010.02.010 20609886

[3] Tanko LB, Christiansen C, Cox DA, Geiger MJ, McNabb MA, Cummings SR (2005) Relationship between osteoporosis and cardiovascular disease in postmenopausal women. J Bone Miner Res 20: 1912–1920. 10.1359/JBMR.050711 16234963

[4] Kado DM, Browner WS, Blackwell T, Gore R, Cummings SR (2000) Rate of bone loss is associated with mortality in older women: a prospective study. J Bone Miner Res 15: 1974–1980. 10.1359/jbmr.2000.15.10.1974 11028450

[5] Lawlor DA, Sattar N, Sayers A, Tobias JH (2012) The association of fasting insulin, glucose, and lipids with bone mass in adolescents: findings from a cross-sectional study. J Clin Endocrinol Metab 97: 2068–2076. jc.2011-2721 [pii]; 10.1210/jc.2011-2721 22492875 PMC3387416

[6] Li S, Guo H, Liu Y, Wu F, Zhang H, Zhang Z, et al (2015) Relationships of serum lipid profiles and bone mineral density in postmenopausal Chinese women. Clin Endocrinol (Oxf) 82: 53–58. 10.1111/cen.12616 25279969

[7] Kim T, Park S, Pak YS, Lee S, Lee EH (2013) Association between metabolic syndrome and bone mineral density in Korea: the Fourth Korea National Health and Nutrition Examination Survey (KNHANES IV), 2008. J Bone Miner Metab 31: 652–662. 10.1007/s00774-013-0459-4 23543212

[8] Kim YH, Nam GE, Cho KH, Choi YS, Kim SM, Han BD, et al (2013) Low bone mineral density is associated with dyslipidemia in South Korean men: the 2008–2010 Korean National Health and Nutrition Examination Survey. Endocr J 60: 1179–1189. DN/JST.JSTAGE/endocrj/EJ13-0224 [pii]. 23877056 10.1507/endocrj.ej13-0224

[9] Buizert PJ, van Schoor NM, Lips P, Deeg DJ, Eekhoff EM (2009) Lipid levels: a link between cardiovascular disease and osteoporosis? J Bone Miner Res 24: 1103–1109. 10.1359/jbmr.081262 19113906

[10] Garg MK, Marwaha RK, Tandon N, Bhadra K, Mahalle N (2014) Relationship of lipid parameters with bone mineral density in Indian population. Indian J Endocrinol Metab 18: 325–332. 10.4103/2230-8210.131165;IJEM-18-325 [pii]. 24944926 PMC4056130

[11] Liu J, Zhu LP, Yang XL, Huang HL, Ye DQ (2013) HMG-CoA reductase inhibitors (statins) and bone mineral density: a meta-analysis. Bone 54: 151–156. S8756-3282(13)00061-6 [pii]; 10.1016/j.bone.2013.01.044 23388418

[12] Lee HT, Shin J, Min SY, Lim YH, Kim KS, Kim SG, et al (2014) The relationship between bone mineral density and blood pressure in the Korean elderly population: the Korea National Health and Nutrition Examination Survey, 2008–2011. Clin Exp Hypertens 1–6. 10.3109/10641963.2014.933971 25057784

[13] Yazici S, Yazici M, Korkmaz U, Engin EM, Erdem BA, Erden I, et al (2011) Relationship between blood pressure levels and bone mineral density in postmenopausal Turkish women. Arch Med Sci 7: 264–270. 10.5114/aoms.2011.22077;AMS-7-2-264 [pii]. 22291766 PMC3258724

[14] Kaplan S, Smith SR, Zuckerman IH (2010) Blood pressure and bone mineral density in premenopausal and postmenopausal women. J Womens Health (Larchmt) 19: 1209–1215. 10.1089/jwh.2009.1587 20545562

[15] Jackuliak P, Payer J (2014) Osteoporosis, fractures, and diabetes. Int J Endocrinol 2014: 820615 10.1155/2014/820615 25050121 PMC4094869

[16] Vestergaard P (2007) Discrepancies in bone mineral density and fracture risk in patients with type 1 and type 2 diabetes—a meta-analysis. Osteoporos Int 18: 427–444. 10.1007/s00198-006-0253-4 17068657

[17] Oei L, Zillikens MC, Dehghan A, Buitendijk GH, Castano-Betancourt MC, Estrada K, et al (2013) High bone mineral density and fracture risk in type 2 diabetes as skeletal complications of inadequate glucose control: the Rotterdam Study. Diabetes Care 36: 1619–1628. dc12-1188 [pii]; 10.2337/dc12-1188 23315602 PMC3661786

[18] Khan TS, Fraser LA (2015) Type 1 diabetes and osteoporosis: from molecular pathways to bone phenotype. J Osteoporos 2015: 174186 10.1155/2015/174186 25874154 PMC4385591

[19] Sayers A, Lawlor DA, Sattar N, Tobias JH (2012) The association between insulin levels and cortical bone: findings from a cross-sectional analysis of pQCT parameters in adolescents. J Bone Miner Res 27: 610–618. 10.1002/jbmr.1467 22095452 PMC3378703

[20] Billings LK, Hsu YH, Ackerman RJ, Dupuis J, Voight BF, Rasmussen-Torvik LJ, et al (2012) Impact of common variation in bone-related genes on type 2 diabetes and related traits. Diabetes 61: 2176–2186. db11-1515 [pii]; 10.2337/db11-1515 22698912 PMC3402303

[21] Kim CJ, Oh KW, Rhee EJ, Kim KH, Jo SK, Jung CH, et al (2009) Relationship between body composition and bone mineral density (BMD) in perimenopausal Korean women. Clin Endocrinol (Oxf) 71: 18–26. CEN3452 [pii]; 10.1111/j.1365-2265.2008.03452.x 19178508

[22] Zillikens MC, Uitterlinden AG, van Leeuwen JP, Berends AL, Henneman P, van Dijk KW, et al (2010) The role of body mass index, insulin, and adiponectin in the relation between fat distribution and bone mineral density. Calcif Tissue Int 86: 116–125. 10.1007/s00223-009-9319-6 19957167 PMC2809303

[23] Janicka A, Wren TA, Sanchez MM, Dorey F, Kim PS, Mittelman SD, et al (2007) Fat mass is not beneficial to bone in adolescents and young adults. J Clin Endocrinol Metab 92: 143–147. jc.2006-0794 [pii]; 10.1210/jc.2006-0794 17047019

[24] Ackert-Bicknell CL (2012) HDL cholesterol and bone mineral density: is there a genetic link? Bone 50: 525–533. S8756-3282(11)01080-5 [pii]; 10.1016/j.bone.2011.07.002 21810493 PMC3236254

[25] Estrada K, Styrkarsdottir U, Evangelou E, Hsu YH, Duncan EL, Ntzani EE, et al (2012) Genome-wide meta-analysis identifies 56 bone mineral density loci and reveals 14 loci associated with risk of fracture. Nat Genet 44: 491–501. ng.2249 [pii]; 10.1038/ng.2249 22504420 PMC3338864

[26] Teslovich TM, Musunuru K, Smith AV, Edmondson AC, Stylianou IM, Koseki M, et al (2010) Biological, clinical and population relevance of 95 loci for blood lipids. Nature 466: 707–713. nature09270 [pii]; 10.1038/nature09270 20686565 PMC3039276

[27] Barrett JC, Clayton DG, Concannon P, Akolkar B, Cooper JD, Erlich HA, et al (2009) Genome-wide association study and meta-analysis find that over 40 loci affect risk of type 1 diabetes. Nat Genet 41: 703–707. ng.381 [pii]; 10.1038/ng.381 19430480 PMC2889014

[28] Voight BF, Scott LJ, Steinthorsdottir V, Morris AP, Dina C, Welch RP, et al (2010) Twelve type 2 diabetes susceptibility loci identified through large-scale association analysis. Nat Genet 42: 579–589. ng.609 [pii]; 10.1038/ng.609 20581827 PMC3080658

[29] Ehret GB, Munroe PB, Rice KM, Bochud M, Johnson AD, Chasman DI, Smith AV, et al (2011) Genetic variants in novel pathways influence blood pressure and cardiovascular disease risk. Nature 478: 103–109. nature10405 [pii]; 10.1038/nature10405 21909115 PMC3340926

[30] Sivakumaran S, Agakov F, Theodoratou E, Prendergast JG, Zgaga L, Manolio T, et al (2011) Abundant pleiotropy in human complex diseases and traits. Am J Hum Genet 89: 607–618. S0002-9297(11)00438-1 [pii]; 10.1016/j.ajhg.2011.10.004 22077970 PMC3213397

[31] Schizophrenia Psychiatric Genome-Wide Association Study (GWAS) Consortium. (2011) Genome-wide association study identifies five new schizophrenia loci. Nat Genet 43: 969–976. ng.940 [pii]; 10.1038/ng.940 21926974 PMC3303194

[32] Liu JZ, Hov JR, Folseraas T, Ellinghaus E, Rushbrook SM, Doncheva NT, et al (2013) Dense genotyping of immune-related disease regions identifies nine new risk loci for primary sclerosing cholangitis. Nat Genet 45: 670–675. ng.2616 [pii]; 10.1038/ng.2616 23603763 PMC3667736

[33] Andreassen OA, Djurovic S, Thompson WK, Schork AJ, Kendler KS, O'Donovan MC, et al (2013) Improved detection of common variants associated with schizophrenia by leveraging pleiotropy with cardiovascular-disease risk factors. Am J Hum Genet 92: 197–209. S0002-9297(13)00030-X [pii]; 10.1016/j.ajhg.2013.01.001 23375658 PMC3567279

[34] Andreassen OA, McEvoy LK, Thompson WK, Wang Y, Reppe S, Schork AJ, et al (2014) Identifying common genetic variants in blood pressure due to polygenic pleiotropy with associated phenotypes. Hypertension 63: 819–826. HYPERTENSIONAHA.113.02077 [pii]; 10.1161/HYPERTENSIONAHA.113.02077 24396023 PMC3984909

[35] Schork AJ, Thompson WK, Pham P, Torkamani A, Roddey JC, Sullivan PF, et al (2013) All SNPs are not created equal: genome-wide association studies reveal a consistent pattern of enrichment among functionally annotated SNPs. PLoS Genet 9: e1003449 10.1371/journal.pgen.1003449;PGENETICS-D-12-02185 [pii]. 23637621 PMC3636284

[36] Lin DY, Sullivan PF (2009) Meta-analysis of genome-wide association studies with overlapping subjects. Am J Hum Genet 85: 862–872. S0002-9297(09)00515-1 [pii]; 10.1016/j.ajhg.2009.11.001 20004761 PMC2790578

[37] Andreassen OA, Zuber V, Thompson WK, Schork AJ, Bettella F, Djurovic S, et al (2014) Shared common variants in prostate cancer and blood lipids. Int J Epidemiol 43: 1205–1214. dyu090 [pii]; 10.1093/ije/dyu090 24786909 PMC4121563

[38] Reppe S, Refvem H, Gautvik VT, Olstad OK, Hovring PI, Reinholt FP, et al (2010) Eight genes are highly associated with BMD variation in postmenopausal Caucasian women. Bone 46: 604–612. 10.1016/j.bone.2009.11.007 19922823

[39] Jemtland R, Holden M, Reppe S, Olstad OK, Reinholt FP, Gautvik VT, et al (2011) Molecular disease map of bone characterizing the postmenopausal osteoporosis phenotype. J Bone Miner Res 26: 1793–1801. 10.1002/jbmr.396 21452281

[40] Willer CJ, Schmidt EM, Sengupta S, Peloso GM, Gustafsson S, Kanoni S, et al (2013) Discovery and refinement of loci associated with lipid levels. Nat Genet 45: 1274–1283. ng.2797 [pii]; 10.1038/ng.2797 24097068 PMC3838666

[41] Liu Q, Chen Y, Auger-Messier M, Molkentin JD (2012) Interaction between NFkappaB and NFAT coordinates cardiac hypertrophy and pathological remodeling. Circ Res 110: 1077–1086. CIRCRESAHA.111.260729 [pii]; 10.1161/CIRCRESAHA.111.260729 22403241 PMC3341669

[42] Fromigue O, Hay E, Barbara A, Marie PJ (2010) Essential role of nuclear factor of activated T cells (NFAT)-mediated Wnt signaling in osteoblast differentiation induced by strontium ranelate. J Biol Chem 285: 25251–25258. M110.110502 [pii]; 10.1074/jbc.M110.110502 20554534 PMC2919088

[43] Saidak Z, Hay E, Marty C, Barbara A, Marie PJ (2012) Strontium ranelate rebalances bone marrow adipogenesis and osteoblastogenesis in senescent osteopenic mice through NFATc/Maf and Wnt signaling. Aging Cell 11: 467–474. 10.1111/j.1474-9726.2012.00804.x 22321691

[44] Strait KA, Stricklett PK, Kohan RM, Kohan DE (2010) Identification of two nuclear factor of activated T-cells (NFAT)-response elements in the 5'-upstream regulatory region of the ET-1 promoter. J Biol Chem 285: 28520–28528. M110.153189 [pii]; 10.1074/jbc.M110.153189 20647310 PMC2937878

[45] Ge Y, Bagnall A, Stricklett PK, Webb D, Kotelevtsev Y, Kohan DE (2008) Combined knockout of collecting duct endothelin A and B receptors causes hypertension and sodium retention. Am J Physiol Renal Physiol 295: F1635–F1640. 90279.2008 [pii]; 10.1152/ajprenal.90279.2008 18784261 PMC2604820

[46] Goettsch C, Rauner M, Hamann C, Sinningen K, Hempel U, Bornstein SR, et al (2011) Nuclear factor of activated T cells mediates oxidised LDL-induced calcification of vascular smooth muscle cells. Diabetologia 54: 2690–2701. 10.1007/s00125-011-2219-0 21701818

[47] Paic F, Igwe JC, Nori R, Kronenberg MS, Franceschetti T, Harrington P, et al (2009) Identification of differentially expressed genes between osteoblasts and osteocytes. Bone 45: 682–692. S8756-3282(09)01634-2 [pii]; 10.1016/j.bone.2009.06.010 19539797 PMC2731004

[48] Dallas SL, Veno PA (2012) Live imaging of bone cell and organ cultures. Methods Mol Biol 816: 425–457. 10.1007/978-1-61779-415-5_26 22130943

[49] Pidsley R, CC YW, Volta M, Lunnon K, Mill J, Schalkwyk LC (2013) A data-driven approach to preprocessing Illumina 450K methylation array data. BMC Genomics 14: 293 1471-2164-14-293 [pii]; 10.1186/1471-2164-14-293 23631413 PMC3769145

[50] Reppe S, Stilgren L, Abrahamsen B, Olstad OK, Cero F, Brixen K, et al (2007) Abnormal muscle and hematopoietic gene expression may be important for clinical morbidity in primary hyperparathyroidism. Am J Physiol Endocrinol Metab 292: E1465–E1473. 17227961 10.1152/ajpendo.00487.2006

[51] Gupta M, Cheung CL, Hsu YH, Demissie S, Cupples LA, Kiel DP, et al (2011) Identification of homogeneous genetic architecture of multiple genetically correlated traits by block clustering of genome-wide associations. J Bone Miner Res 26: 1261–1271. 10.1002/jbmr.333 21611967 PMC3312758

[52] Fitzpatrick DJ, Ryan CJ, Shah N, Greene D, Molony C, Shields DC (2015) Genome-wide epistatic expression quantitative trait loci discovery in four human tissues reveals the importance of local chromosomal interactions governing gene expression. BMC Genomics 16: 109 10.1186/s12864-015-1300-3;s12864-015-1300-3 [pii]. 25765234 PMC4345003

[53] Jin F, Li Y, Dixon JR, Selvaraj S, Ye Z, Lee AY, et al (2013) A high-resolution map of the three-dimensional chromatin interactome in human cells. Nature 503: 290–294. nature12644 [pii]; 10.1038/nature12644 24141950 PMC3838900

