# Supplementary figures and images for: Genetic Sharing with Cardiovascular Disease Risk Factors and Diabetes Reveals Novel Bone Mineral Density Loci
Source: PLoS One. 2015 Dec 22;10(12):e0144531. doi: 10.1371/journal.pone.0144531 (PMC4687843; doi:10.1371/journal.pone.0144531)

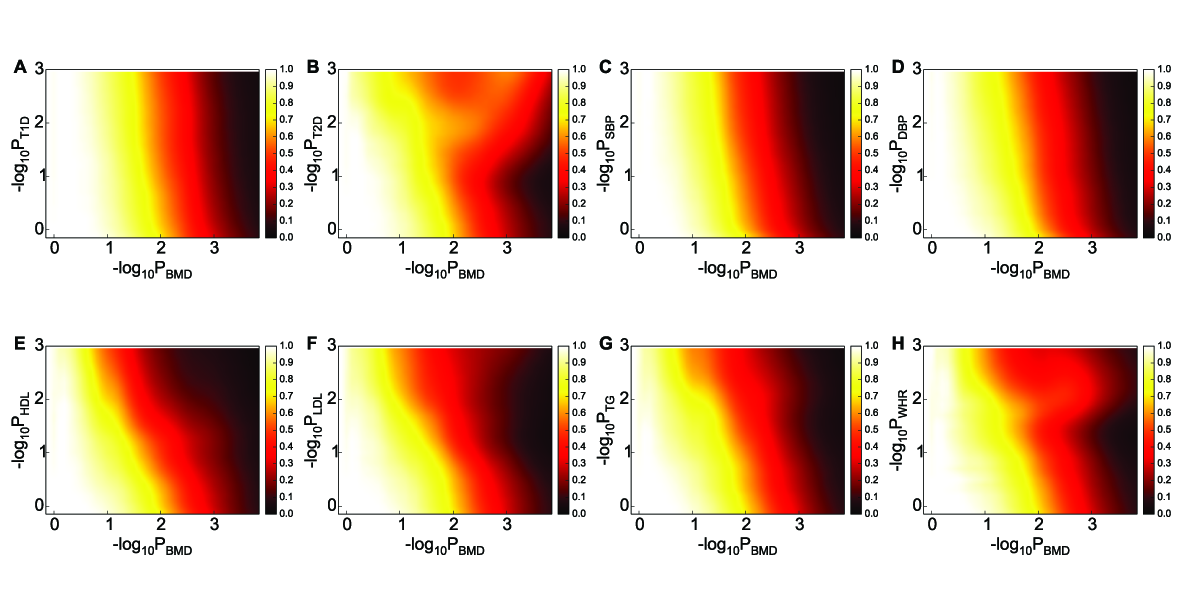

Supplement: S1 Fig — Based on the combination of p-value for the SNPs in femoral neck BMD (PBMD) and that of the pleiotropic trait: A. type 1 diabetes (T1D), B. type 2 diabetes (T2D), C. systolic blood pressure (SBP), D. diastolic blood pressure (DBP), E. high density lipoprotein (HDL), F. low density lipoprotein (LDL), G. triglycerides (TG), and H. waist hip ratio (WHR) we assigned a conditional FDR value to each SNP associated with femoral neck BMD, by interpolation into a 2-D look-up table. Color scale refers to the conditional FDR values. (TIF) [file pone.0144531.s001.tif]

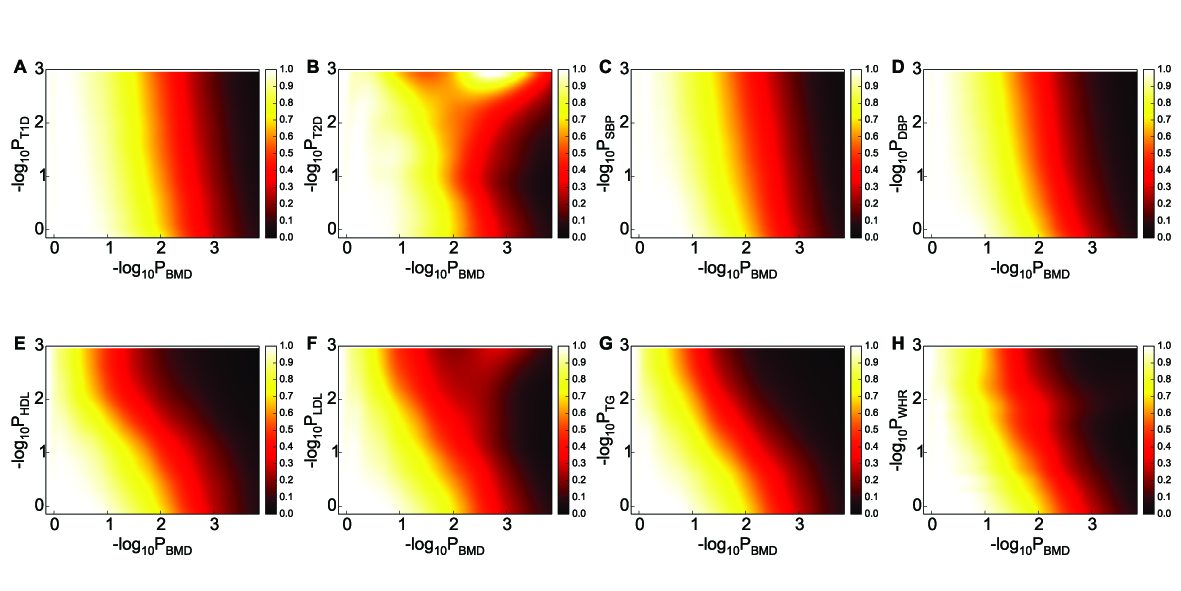

Supplement: S2 Fig — Based on the combination of p-value for the SNPs in lumbar spine BMD (PBMD) and that of the pleiotropic trait: A. type 1 diabetes (T1D), B. type 2 diabetes (T2D), C. systolic blood pressure (SBP), D. diastolic blood pressure (DBP), E. high density lipoprotein (HDL), F. low density lipoprotein (LDL), G. triglycerides (TG), and H. waist hip ratio (WHR), we assigned a conditional FDR value to each SNP associated with lumbar spine BMD, by interpolation into a 2-D look-up table. Color scale refers to the conditional FDR values. (TIF) [file pone.0144531.s002.tif]

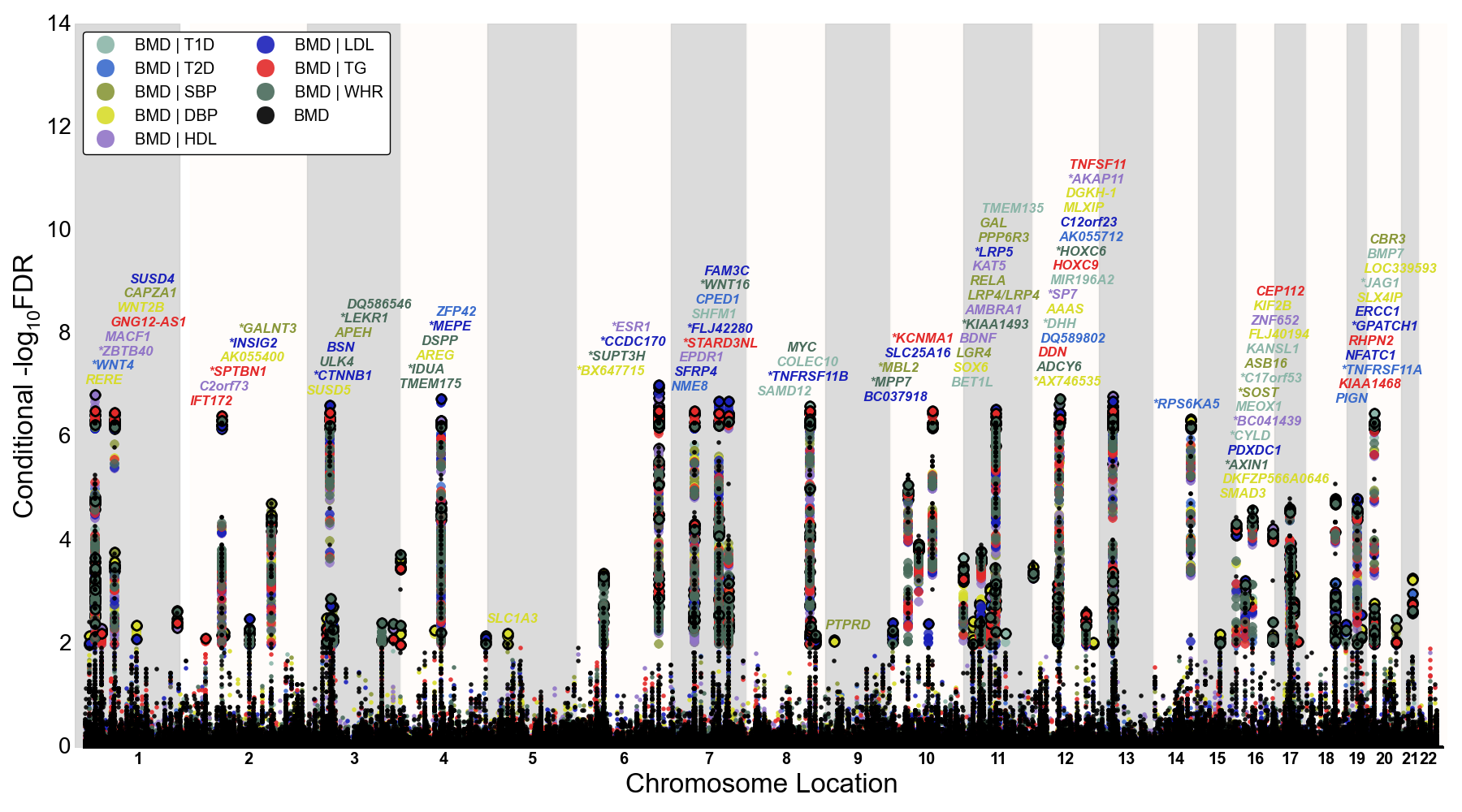

Supplement: S3 Fig — ‘Conditional Manhattan plot’ of conditional–log10 (FDR) values for bone mineral density (BMD, lumbar spine) alone (small black dots) and BMD given the associated phenotypes type 1 diabetes (T1D; BMD|T1D), type 2 diabetes (T2D; BMD|T2D), systolic blood pressure (SBP; BMD|SBP), diastolic blood pressure (DBP; BMD|DBP), high density lipoprotein (HDL; BMD|HDL), low density lipoprotein (LDL; BMD|LDL), triglycerides (TG; BMD|TG), and waist hip ratio (WHR; BMD|WHR). SNPs with conditional–log10 FDR > 2 (i.e. FDR < 0.01) are shown with large points. A black line around the large points indicates the most significant SNP in each LD block and this SNP was annotated with the closest gene, which is listed above the symbols in each locus. Gene symbols were obtained from HGNC gene databases and colored in line with the second phenotype, which gives the minimal conditional FDR value. Genes previously reported by other studies were marked by stars (*). (TIF) [file pone.0144531.s003.tif]

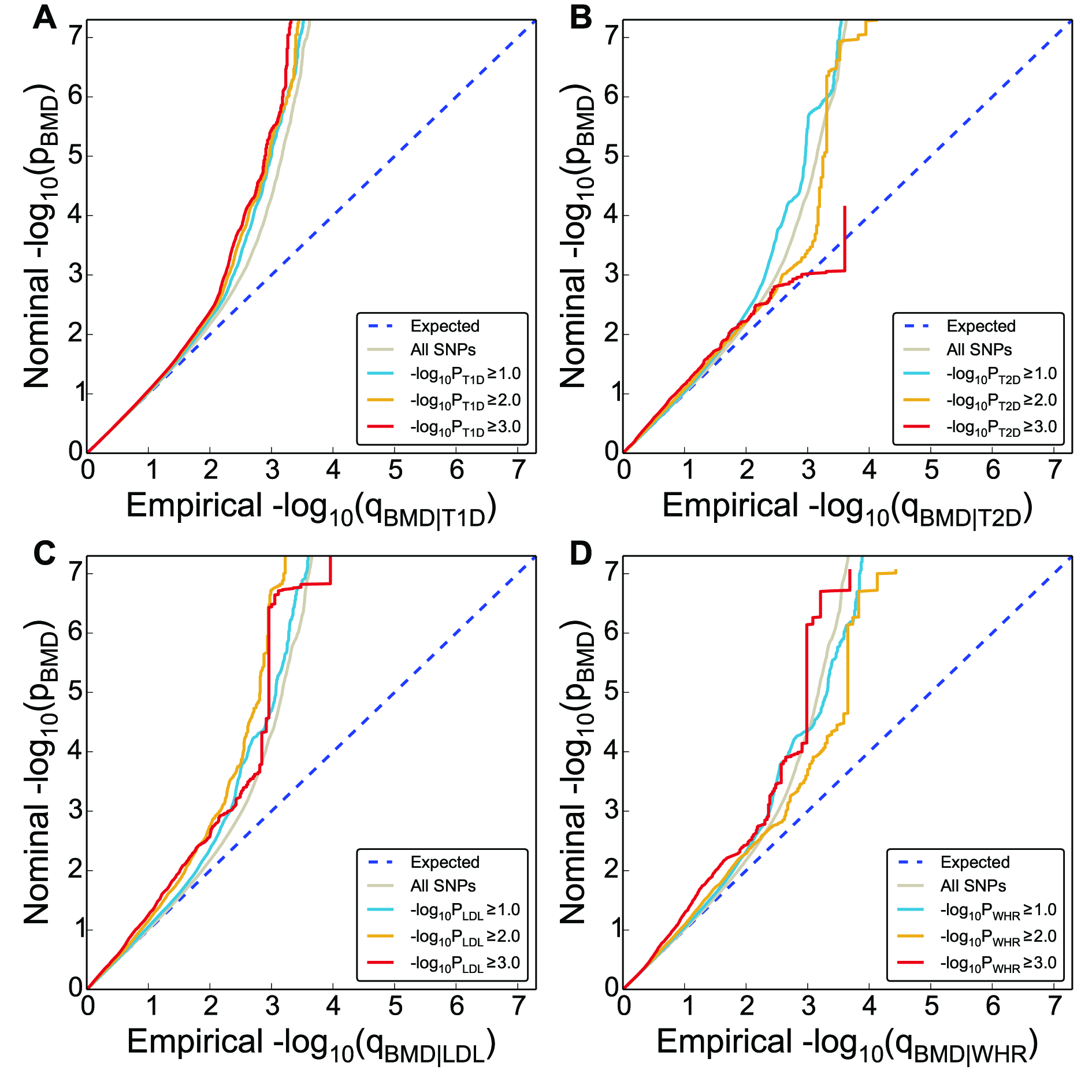

Supplement: S4 Fig — Conditional Q-Q plot of nominal versus empirical -log10 p-values (corrected for inflation) in bone mineral density (BMD, femoral neck) below the standard GWAS threshold of p < 5x10^-8^ as a function of significance of association with CVD risk factors, including type 1 diabetes (T1D), type 2 diabetes (T2D), low density lipoprotein (LDL) and waist hip ratio (WHR) at the level of -log10(p) ≥ 0 (all SNPs),–log10(p) ≥ 1,–log10(p) ≥ 2,–log10(p) ≥ 3 corresponding to p ≤ 1, p ≤ 0.1, p ≤ 0.01, p ≤ 0.001, respectively. Dotted lines indicate the null-hypothesis. (TIF) [file pone.0144531.s004.tif]

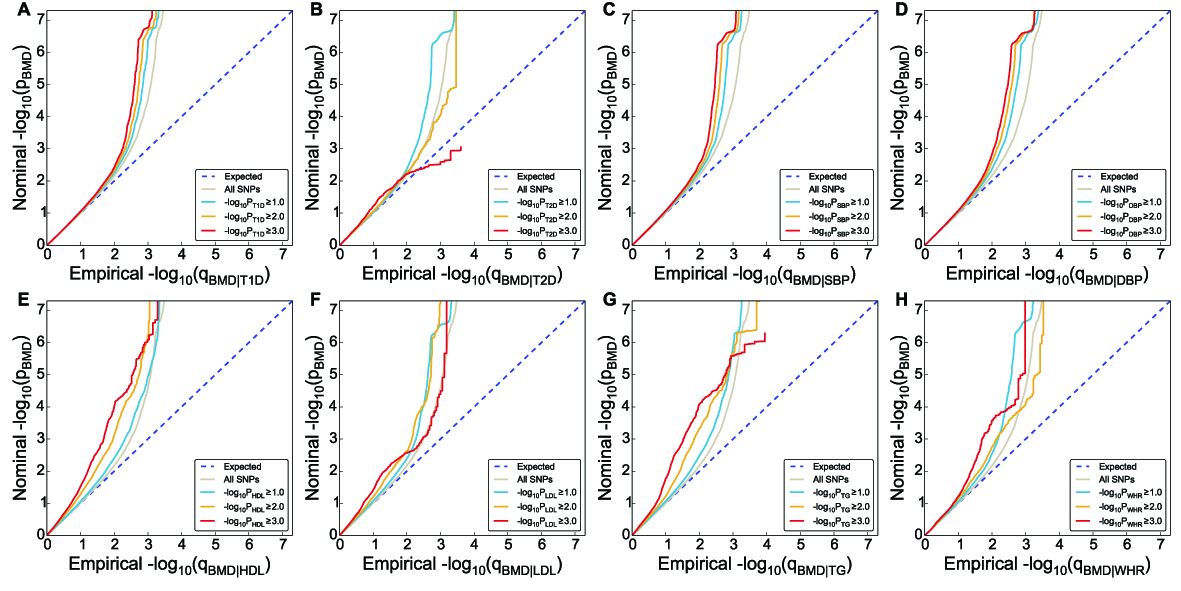

Supplement: S5 Fig — Conditional Q-Q plot of nominal versus empirical -log10 p-values (corrected for inflation) in bone mineral density (BMD, lumbar spine) below the standard GWAS threshold of p < 5x10^-8^ as a function of significance of association with A. type 1 diabetes (T1D), B. type 2 diabetes (T2D), C. systolic blood pressure (SBP), D. diastolic blood pressure (DBP), E. high density lipoprotein (HDL), F. low density lipoprotein (LDL), G. triglycerides (TG), and H. waist hip ratio (WHR) at the level of -log10(p) ≥ 0 (all SNPs),–log10(p) ≥ 1,–log10(p) ≥ 2,–log10(p) ≥ 3 corresponding to p ≤ 1, p ≤ 0.1, p ≤ 0.01, p ≤ 0.001, respectively. Dotted lines indicate the null-hypothesis. (TIF) [file pone.0144531.s005.tif]

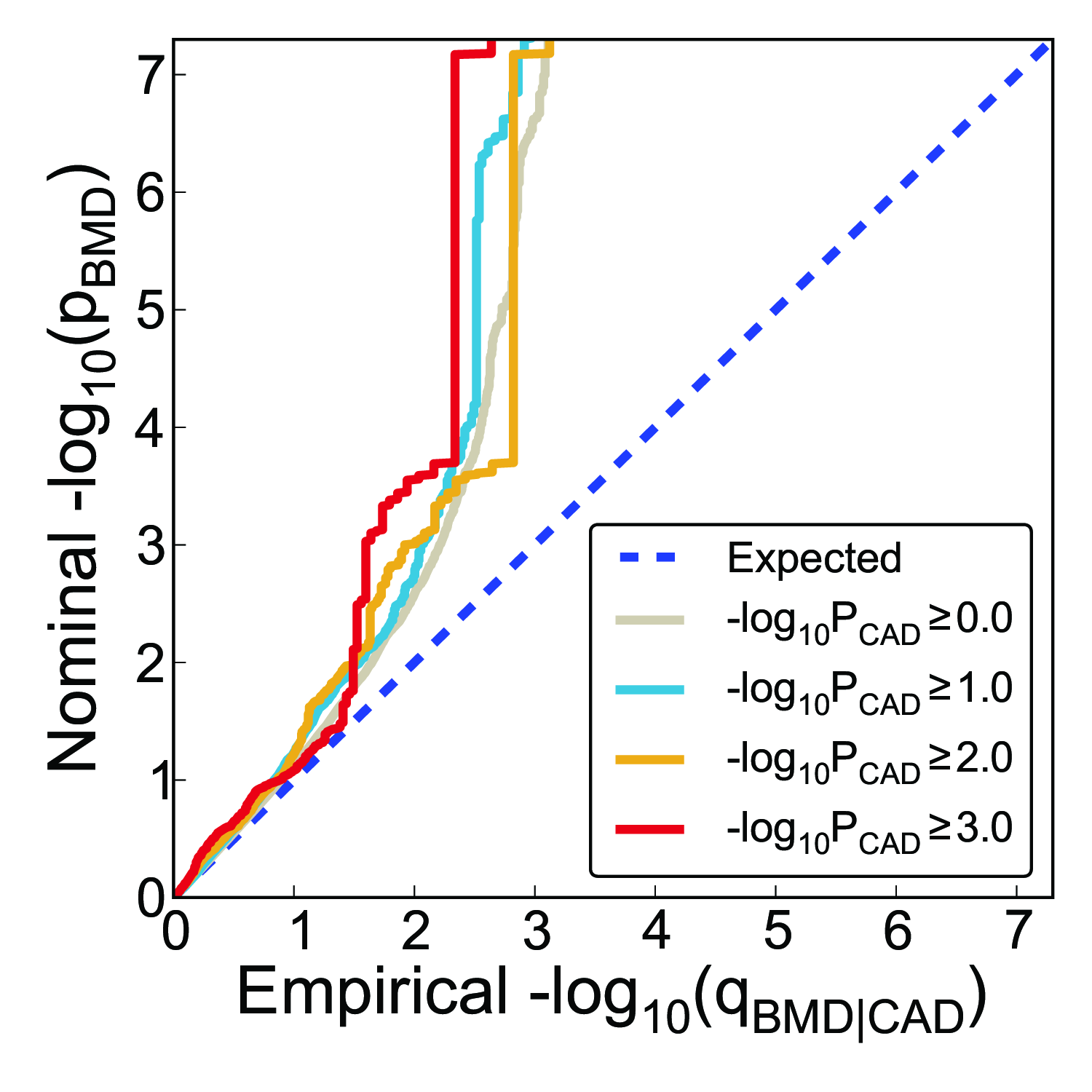

Supplement: S6 Fig — Conditional Q-Q plot of nominal versus empirical -log10 p-values (corrected for inflation) in bone mineral density (BMD, femoral neck) below the standard GWAS threshold of p < 5x10^-8^ as a function of significance of association with Coronary Artery Disease (CAD) at the level of -log10(p) ≥ 0 (all SNPs),–log10(p) ≥ 1,–log10(p) ≥ 2,–log10(p) ≥ 3 corresponding to p ≤ 1, p ≤ 0.1, p ≤ 0.01, p ≤ 0.001, respectively. Dotted lines indicate the null-hypothesis. (TIF) [file pone.0144531.s006.tif]

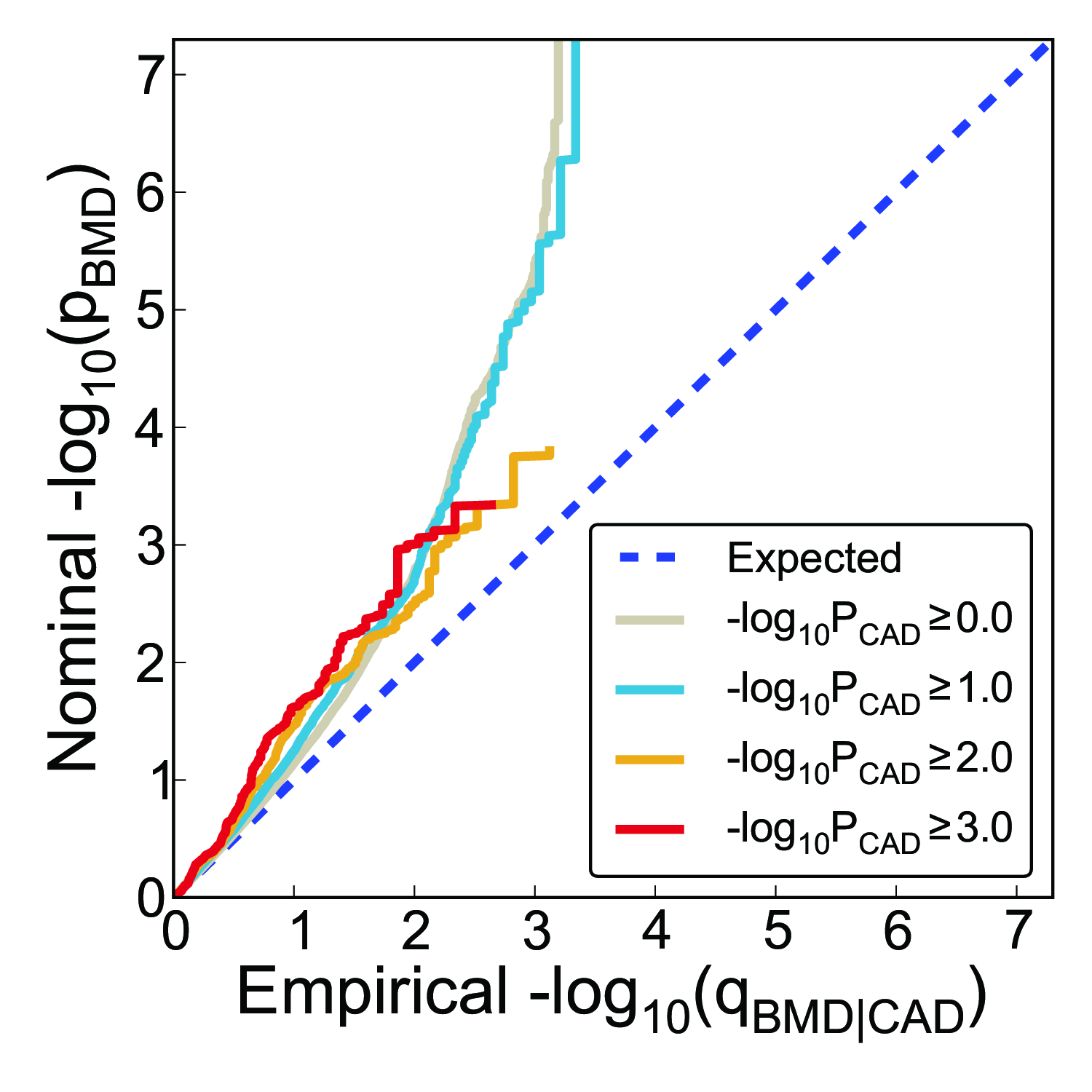

Supplement: S7 Fig — Conditional Q-Q plot of nominal versus empirical -log10 p-values (corrected for inflation) in bone mineral density (BMD, lumbar spine) below the standard GWAS threshold of p < 5x10^-8^ as a function of significance of association with Coronary Artery Disease (CAD) at the level of -log10(p) ≥ 0 (all SNPs),–log10(p) ≥ 1,–log10(p) ≥ 2,–log10(p) ≥ 3 corresponding to p ≤ 1, p ≤ 0.1, p ≤ 0.01, p ≤ 0.001, respectively. Dotted lines indicate the null-hypothesis. (TIF) [file pone.0144531.s007.tif]
