# Supplementary material for: Genetic Sharing with Cardiovascular Disease Risk Factors and Diabetes Reveals Novel Bone Mineral Density Loci
Source: PLoS One. 2015 Dec 22;10(12):e0144531. doi: 10.1371/journal.pone.0144531 (PMC4687843; doi:10.1371/journal.pone.0144531)
Supplement: S1 File — (DOC) [file pone.0144531.s008.doc]

**SUPPLEMENTARY MATERIALS AND METHODS**

**Details of statistical analysis**

***Conditional Q-Q plots***

Q-Q plots compare a nominal probability distribution against an empirical distribution. In the presence of all null relationships, nominal p-values form a straight line on a Q-Q plot when plotted against the empirical distribution. For each phenotype, for all SNPs and for each categorical subset (strata), -log_10_ nominal p-values were plotted against -log_10_ empirical p-values (conditional Q-Q plots). Leftward deflections of the observed distribution from the projected null line reflect increased tail probabilities in the distribution of test statistics (z-scores) and consequently an over-abundance of low p-values compared to that expected by chance, also termed ‘enrichment’.

Under large-scale testing paradigms, such as GWAS, quantitative estimates of likely true associations can be estimated from the distributions of summary statistics.^(1,2)^ A common method for visualizing the enrichment of statistical association relative to that expected under the global null hypothesis is through Q-Q plots of nominal p-values obtained from GWAS summary statistics. The usual Q-Q curve has as the y-ordinate the nominal p-value, denoted by “p”, and as the x-ordinate the corresponding value of the empirical cdf, denoted by “q”. Under the global null hypothesis the theoretical distribution is uniform on the interval [0,1]. As is common in GWAS, we instead plot -log_10_ p against -log_10_ q to emphasize tail probabilities of the theoretical and empirical distributions. Therefore, genetic enrichment results in a leftward shift in the Q-Q curve, corresponding to a larger fraction of SNPs with nominal -log_10_ p-value greater than or equal to a given threshold. *Conditional* Q-Q plots are constructed by creating subsets of SNPs based on levels of an auxiliary measure for each SNP, and computing Q-Q plots separately for each level. If SNP enrichment is captured by variation in the auxiliary measure, this is expressed as successive leftward deflections in a conditional Q-Q plot as levels of the auxiliary measure increase.

***Genomic Control***

The empirical null distribution in GWAS is affected by global variance inflation due to population stratification and cryptic relatedness^(3)^ and deflation due to over-correction of test statistics for polygenic traits by standard genomic control methods. We applied a control method leveraging only intergenic SNPs, which are likely depleted for true associations.^(4)^ First, we annotated the SNPs to genic (5’UTR, exon, intron, 3’UTR) and intergenic regions using information from the 1000 Genomes Project (1KGP). Then, we used intergenic SNPs to estimate the genomic inflation factor, λ_GC_, because their relative depletion of associations suggests that they provide a robust estimate of true null effects and thus seem a better category for genomic control than all SNPs. We converted all p-values to z-scores and for each phenotype computed the inflation factor, λ_GC_ as the median z-score squared divided by the expected median of a chi-square distribution with one degree of freedom and divided all test statistics by λ_GC_. For more information, see Andreassen et al.^(5)^

***Conditional Q-Q plots for pleiotropic enrichment***

To assess pleiotropic enrichment, we used Q-Q plot conditional by ‘pleiotropic’ effects as described in detail earlier.^(5)^ For a given associated phenotype, enrichment for pleiotropic signals is present if the degree of deflection from the expected null line is dependent on SNP associations with the second phenotype. We constructed conditional Q-Q plots of empirical quantiles of nominal –log_10_(p) values for SNP association with blood lipids for all SNPs, and for subsets (strata) of SNPs determined by the nominal p-values of their association with a given immune-mediated disease. Specifically, we computed the empirical cumulative distribution of nominal p-values for a given phenotype for all SNPs and for SNPs with significance levels below the indicated cut-offs for the other phenotype (–log_10_(p) ≥ 0, –log_10_(p) ≥ 1, –log_10_(p) ≥2, –log_10_(p) ≥3 corresponding to p < 1, p < 0.1, p < 0.01, p < 0.001, respectively). The nominal p-values (–log_10_(p)) are plotted on the y-axis, and the empirical quantiles (–log_10_(q), where q=1-cdf(p)) are plotted on the x-axis. To assess for polygenic effects below the standard GWAS significance threshold, we focused the conditional Q-Q plots on SNPs with nominal –log_10_(p) < 7.3 (corresponding to p > 5x10^-8^).

***Conditional True Discovery Rate (TDR)***

Enrichment seen in the conditional Q-Q plots can be directly interpreted in terms of TDR (equivalent to one minus the FDR).^(6)^ We applied a recently developed conditional FDR method.^(4,5,7)^ Specifically, for a given p-value cutoff, the FDR is defined as

FDR(p) = π_0_F_0_(p) / F(p), [1]

where π _0_ is the proportion of null SNPs, F_0_ is the null cdf, and F is the cdf of all SNPs, both null and non-null; see below for details on this simple mixture model formulation.^(8)^ Under the null hypothesis, F_0_ is the cdf of the uniform distribution on the unit interval [0,1], so that Eq. [1] reduces to

FDR(p) = π_0_ p / F(p), [2]

The cdf F can be estimated by the empirical cdf q =N_p_ / N, where N_p_ is the number of SNPs with p-values less than or equal to p, and N is the total number of SNPs. Replacing F by q in Eq. [2], we get

Estimated FDR(p) = π_0_ p / q, [3]

which is biased upwards as an estimate of the FDR.^(8)^ Replacing π_0_ in Equation [3] with unity gives an estimated FDR that is further biased upward;

q* = p/q [4]

If π_0_ is close to one, as is likely true for most GWAS, the increase in bias from Eq. [3] is minimal. The quantity 1 – p/q, is therefore biased downward, and hence is a conservative estimate of the TDR.

Referring to the formulation of the Q-Q plots, we see that q* is equivalent to the nominal p-value divided by the empirical quantile, as defined earlier. Given the -log_10_ of the Q-Q plots we can easily obtain

-log10(q*) = log_10_(q) – log_10_(p) [5]

demonstrating that the (conservatively) estimated FDR is directly related to the horizontal shift of the curves in the conditional Q-Q plots from the expected line x = y, with a larger shift corresponding to a smaller FDR. As before, the estimated TDR can be obtained as 1-FDR. For each range of p-values (stratum) in an associated trait, we calculated the TDR as a function of p-value in blood lipids using each observed p-value as a threshold, according to Eq. [5].

***Additional Analysis performed***

We also investigate the enrichment of BMD conditional on Coronary Artery Diseases (CAD).^(9)^ The level of enrichment for both FN- and LS-BMD is lower than that of other traits. This may because the number of SNPs available in current study for CAD is only 79,000 much less than the other traits (S6 and S7 Figs).

**SUPPLEMENTARY REFERENCES**

1. Efron B. Large-Scale Inference. 1982 Cambridge University Press, Cambridge.

2. Schweder T, Spjotvoll E. Plots of P-values to evaluate many tests simultaneously. Biometrika. 1982;69:493–02.

3. Devlin B, Roeder K. (1999) Genomic Control for Association Studies. Biometrics. 1999;55:997–04

4. Schork AJ, Thompson WK, Pham P, et al. All SNPs Are Not Created Equal: Genome-Wide Association Studies Reveal a Consistent Pattern of Enrichment among Functionally Annotated SNPs. PLoS Genet. 2013;9:e1003449

5. Andreassen OA, Thompson WK, Schork AJ, et al. Improved detection of common variants associated with schizophrenia and bipolar disorder using pleiotropy-informed conditional false discovery rate. PLoS Genet. 2013;9:e1003455

6. Benjamini Y, Hochberg Y. Controlling the False Discovery Rate: A Practical and Powerful Approach to Multiple Testing. J. R. Stat. Soc. Series B Stat. Methodol. 1995;57:289–00.

7. Andreassen OA, Djurovic S, Thompson WK, et al. Improved detection of common variants associated with schizophrenia by leveraging pleiotropy with cardiovascular-disease risk factors. Am. J. Hum. Genet. 2013;92:197–09.

8. Efron B. Size, power and false discovery rates. Ann. Stat. 2007;35:1351–77

9. CARDIoGRAMplusC4D Consortium, Deloukas P, Kanoni S, et al. Large-scale association analysis identifies new risk loci for coronary artery disease. Nat. Genet. 2013;45:25-33

|  |
| --- |

### Supplementary figures

### S1 Fig. Conditional FDR 2-D lookup table for femoral neck BMD

**
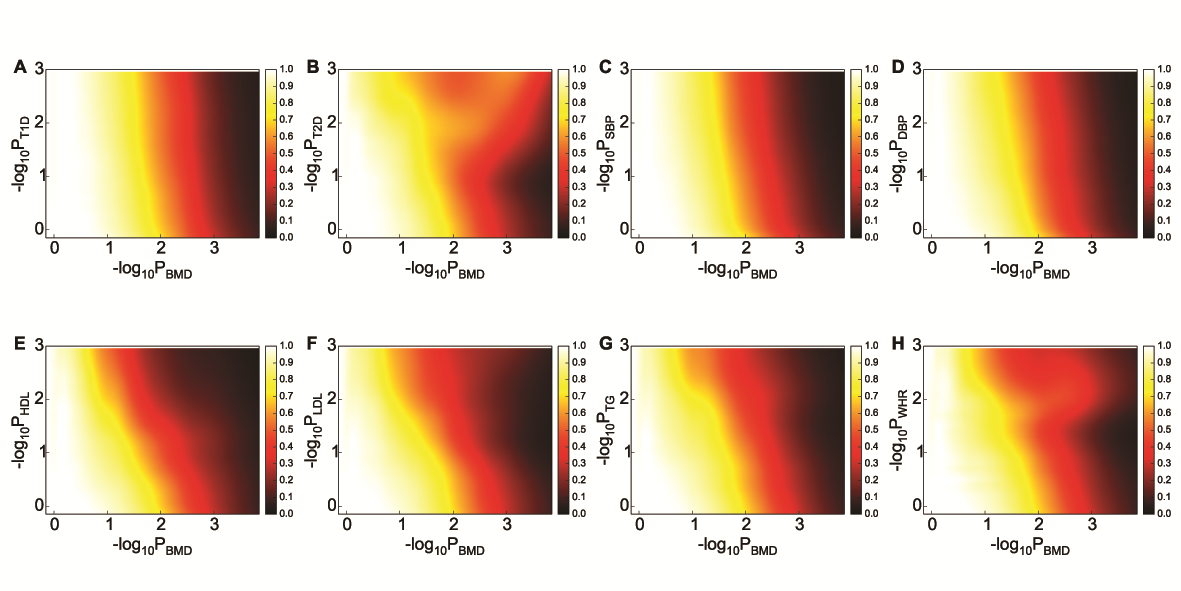
**

Based on the combination of p-value for the SNPs in femoral neck BMD (P_BMD_) and that of the pleiotropic trait: A. type 1 diabetes (T1D), B. type 2 diabetes (T2D), C. systolic blood pressure (SBP), D. diastolic blood pressure (DBP), E. high density lipoprotein (HDL), F. low density lipoprotein (LDL), G. triglycerides (TG), and H. waist hip ratio (WHR) we assigned a conditional FDR value to each SNP associated with femoral neck BMD, by interpolation into a 2-D look-up table. Color scale refers to the conditional FDR values.

###

### S2 Fig. Conditional FDR 2-D lookup table for Lumbar Spine BMD

**
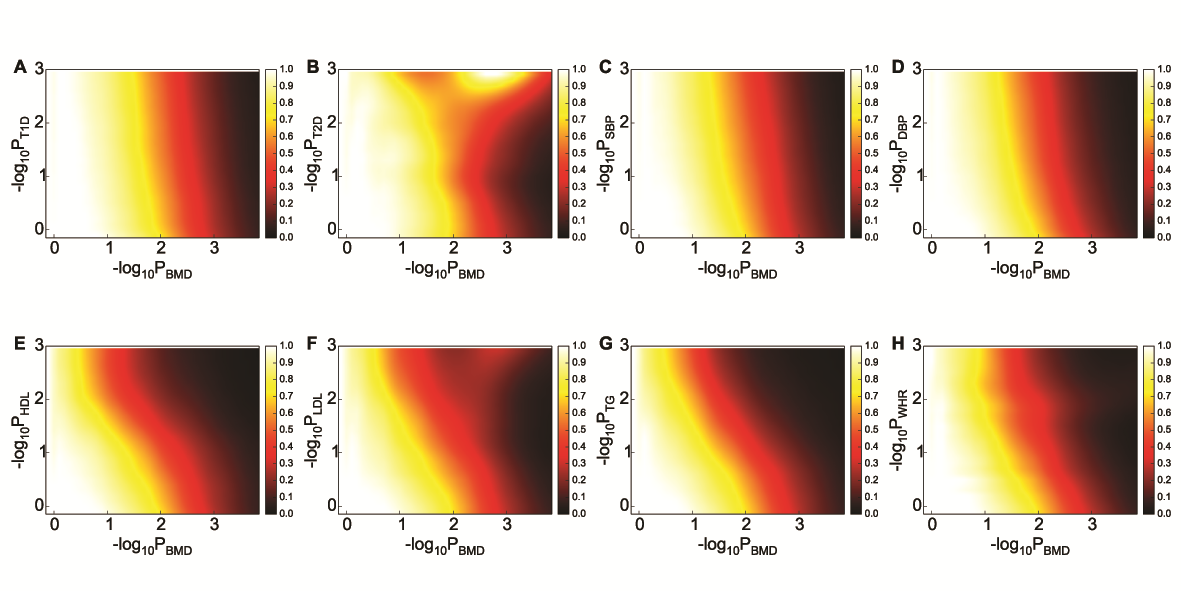
**

Based on the combination of p-value for the SNPs in lumbar spine BMD (P_BMD_) and that of the pleiotropic trait: A. type 1 diabetes (T1D), B. type 2 diabetes (T2D), C. systolic blood pressure (SBP), D. diastolic blood pressure (DBP), E. high density lipoprotein (HDL), F. low density lipoprotein (LDL), G. triglycerides (TG), and H. waist hip ratio (WHR), we assigned a conditional FDR value to each SNP associated with lumbar spine BMD, by interpolation into a 2-D look-up table. Color scale refers to the conditional FDR values.

### S3 Fig. Conditional FDR Manhattan plots for lumbar spine BMD

**
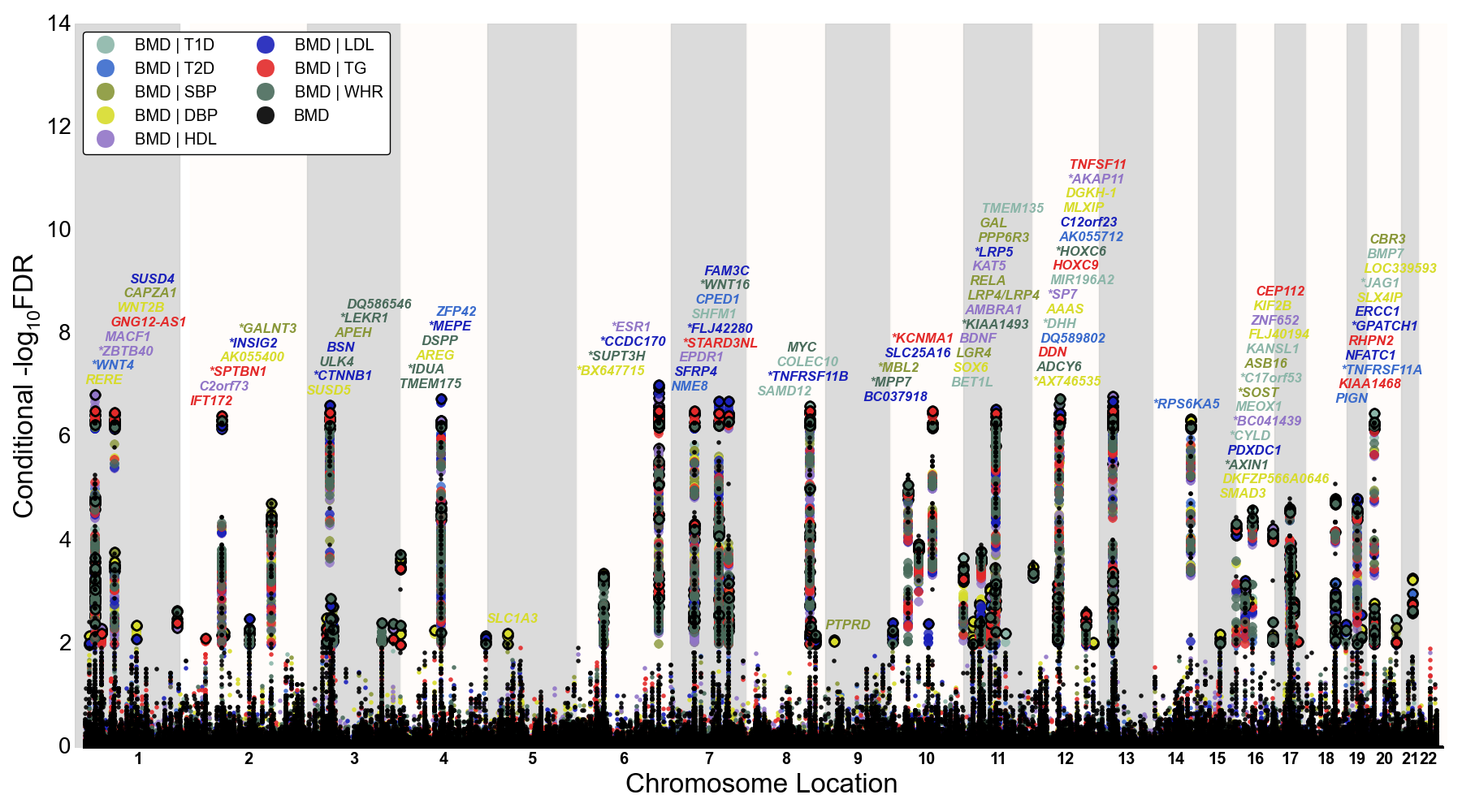
**

### ‘Conditional Manhattan plot’ of conditional –log10 (FDR) values for bone mineral density (BMD, lumbar spine) alone (small black dots) and BMD given the associated phenotypes type 1 diabetes (T1D; BMD|T1D), type 2 diabetes (T2D; BMD|T2D), systolic blood pressure (SBP; BMD|SBP), diastolic blood pressure (DBP; BMD|DBP), high density lipoprotein (HDL; BMD|HDL), low density lipoprotein (LDL; BMD|LDL), triglycerides (TG; BMD|TG), and waist hip ratio (WHR; BMD|WHR). SNPs with conditional –log10 FDR > 2 (i.e. FDR < 0.01) are shown with large points. A black line around the large points indicates the most significant SNP in each LD block and this SNP was annotated with the closest gene, which is listed above the symbols in each locus. Gene symbols were obtained from HGNC gene databases and colored in line with the second phenotype, which gives the minimal conditional FDR value. Genes previously reported by other studies were marked by stars (*).

### S4 Fig. Genetic pleiotropy enrichment

###
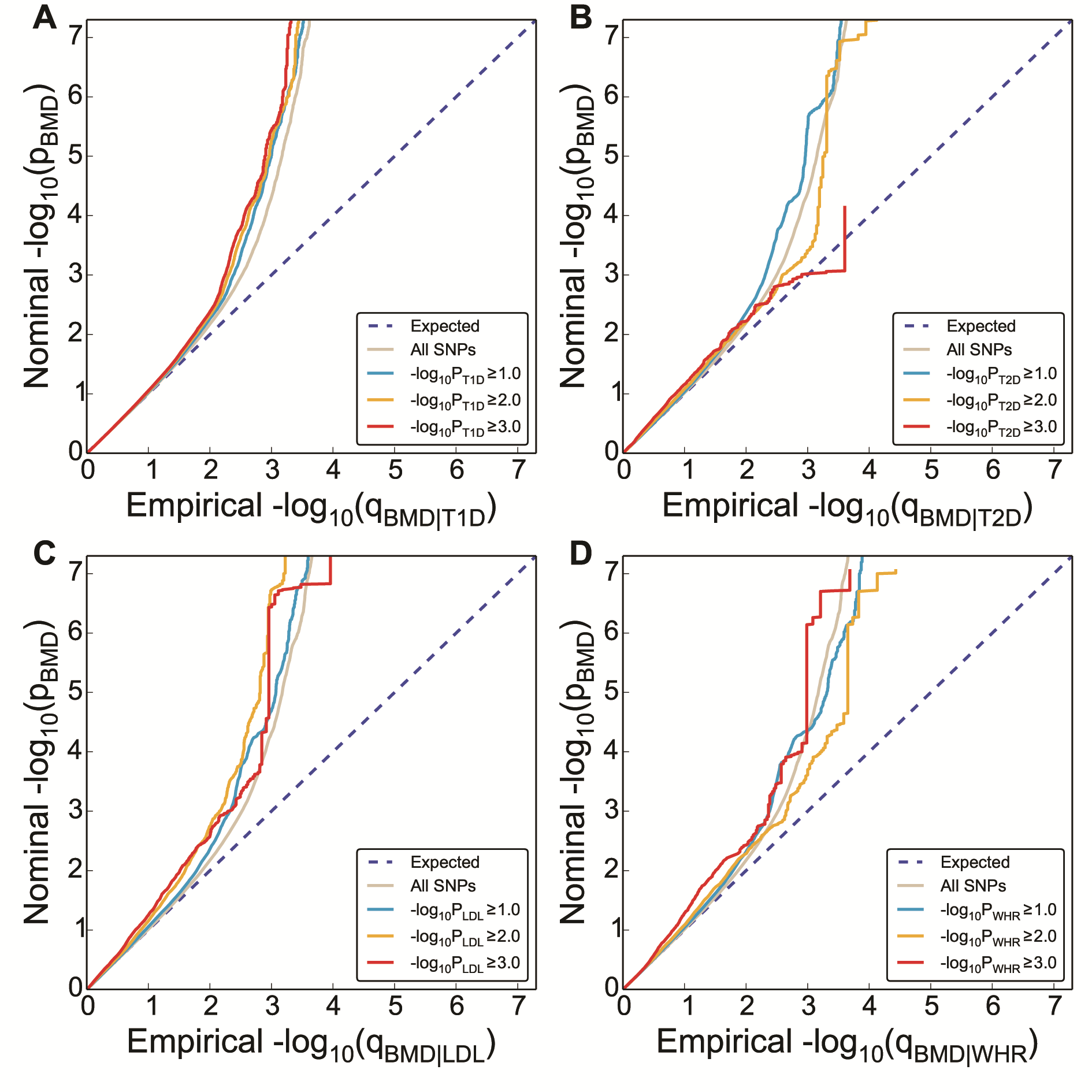


### Conditional Q-Q plot of nominal versus empirical -log10 p-values (corrected for inflation) in bone mineral density (BMD, femoral neck) below the standard GWAS threshold of p < 5x10^-8^ as a function of significance of association with CVD risk factors, including type 1 diabetes (T1D), type 2 diabetes (T2D), low density lipoprotein (LDL) and waist hip ratio (WHR) at the level of -log10(p) ≥ 0 (all SNPs), –log10(p) ≥ 1, –log10(p) ≥ 2, –log10(p) ≥ 3 corresponding to p ≤ 1, p ≤ 0.1, p ≤ 0.01, p ≤ 0.001, respectively. Dotted lines indicate the null-hypothesis.

### S5 Fig. QQ plots for Lumbar Spine-BMD

**
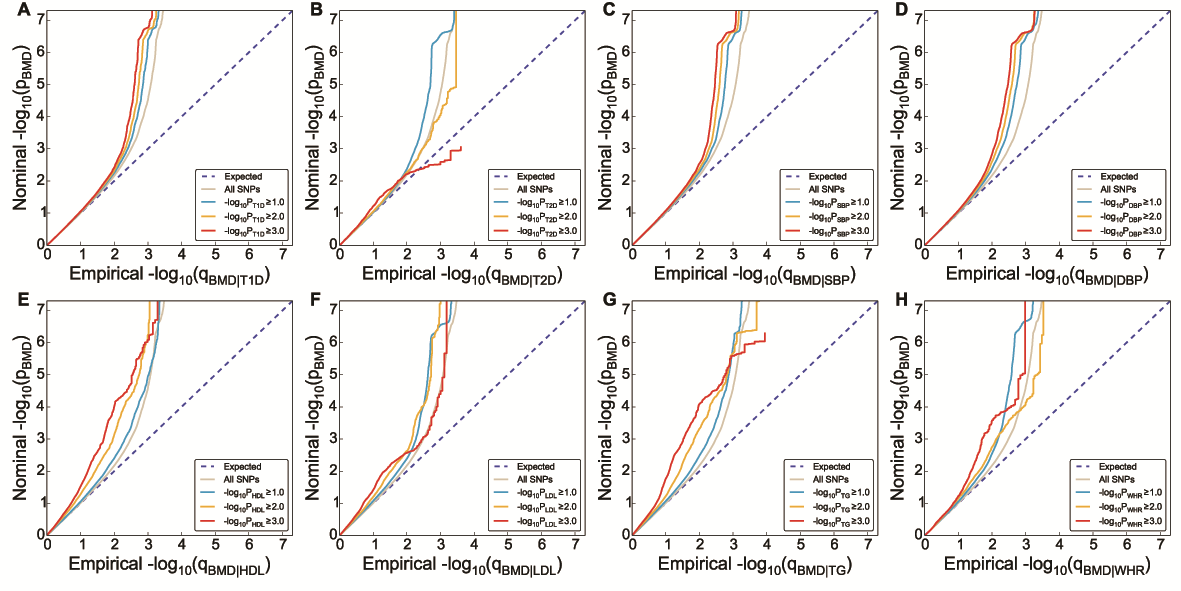
**

Conditional Q-Q plot of nominal versus empirical -log_10_ p-values (corrected for inflation) in bone mineral density (BMD, lumbar spine) below the standard GWAS threshold of p < 5x10^-8^ as a function of significance of association with A. type 1 diabetes (T1D), B. type 2 diabetes (T2D), C. systolic blood pressure (SBP), D. diastolic blood pressure (DBP), E. high density lipoprotein (HDL), F. low density lipoprotein (LDL), G. triglycerides (TG), and H. waist hip ratio (WHR) at the level of -log_10_(p) ≥ 0 (all SNPs), –log_10_(p) ≥ 1, –log_10_(p) ≥ 2, –log_10_(p) ≥ 3 corresponding to p ≤ 1, p ≤ 0.1, p ≤ 0.01, p ≤ 0.001, respectively. Dotted lines indicate the null-hypothesis.

### S6 Fig. Conditional QQ plot for Femoral neck BMD on CAD

**
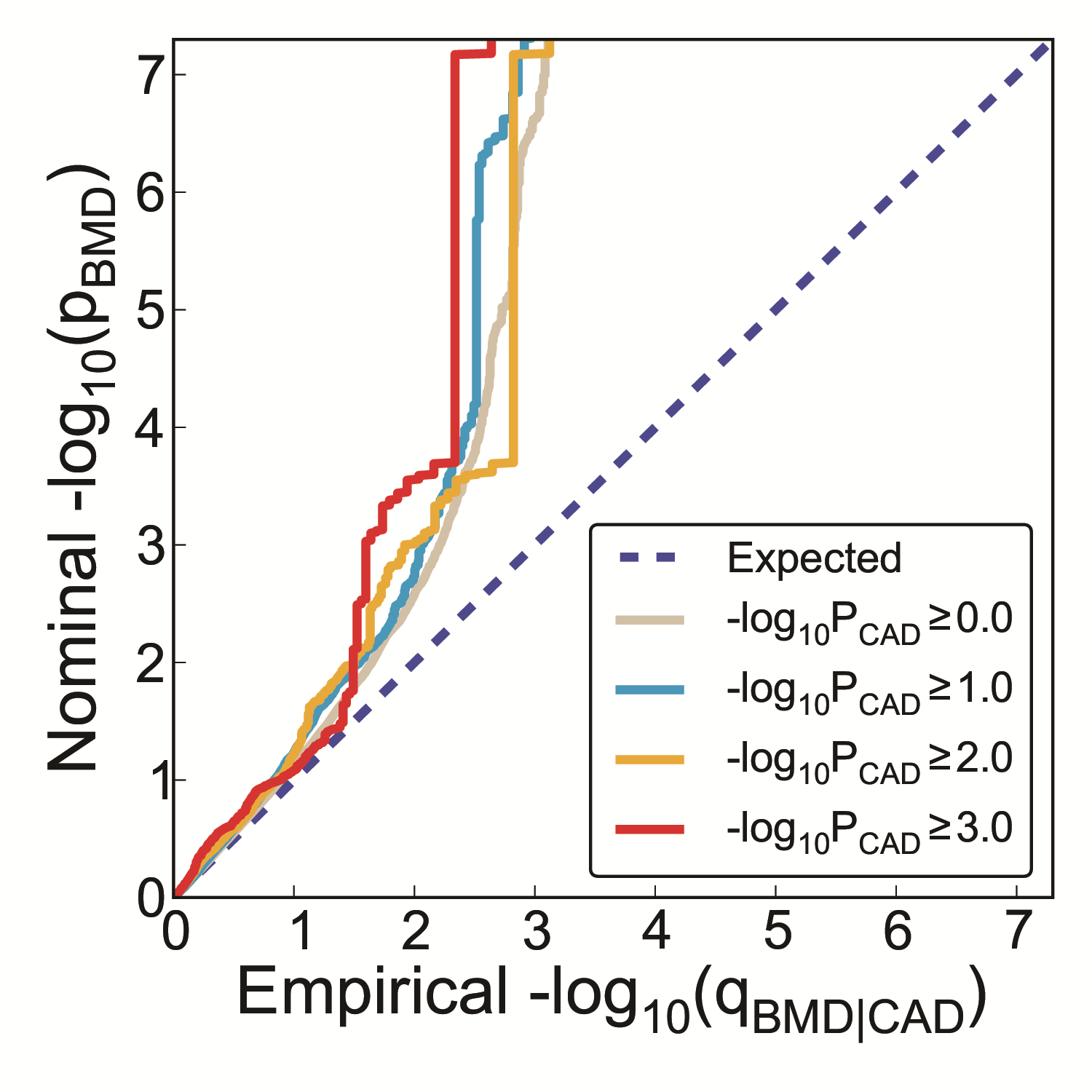
**

Conditional Q-Q plot of nominal versus empirical -log_10_ p-values (corrected for inflation) in bone mineral density (BMD, femoral neck) below the standard GWAS threshold of p < 5x10^-8^ as a function of significance of association with Coronary Artery Disease (CAD) at the level of -log_10_(p) ≥ 0 (all SNPs), –log_10_(p) ≥ 1, –log_10_(p) ≥ 2, –log_10_(p) ≥ 3 corresponding to p ≤ 1, p ≤ 0.1, p ≤ 0.01, p ≤ 0.001, respectively. Dotted lines indicate the null-hypothesis.

### S7 Fig. Conditional QQ plot for lumbar spine BMD on CAD


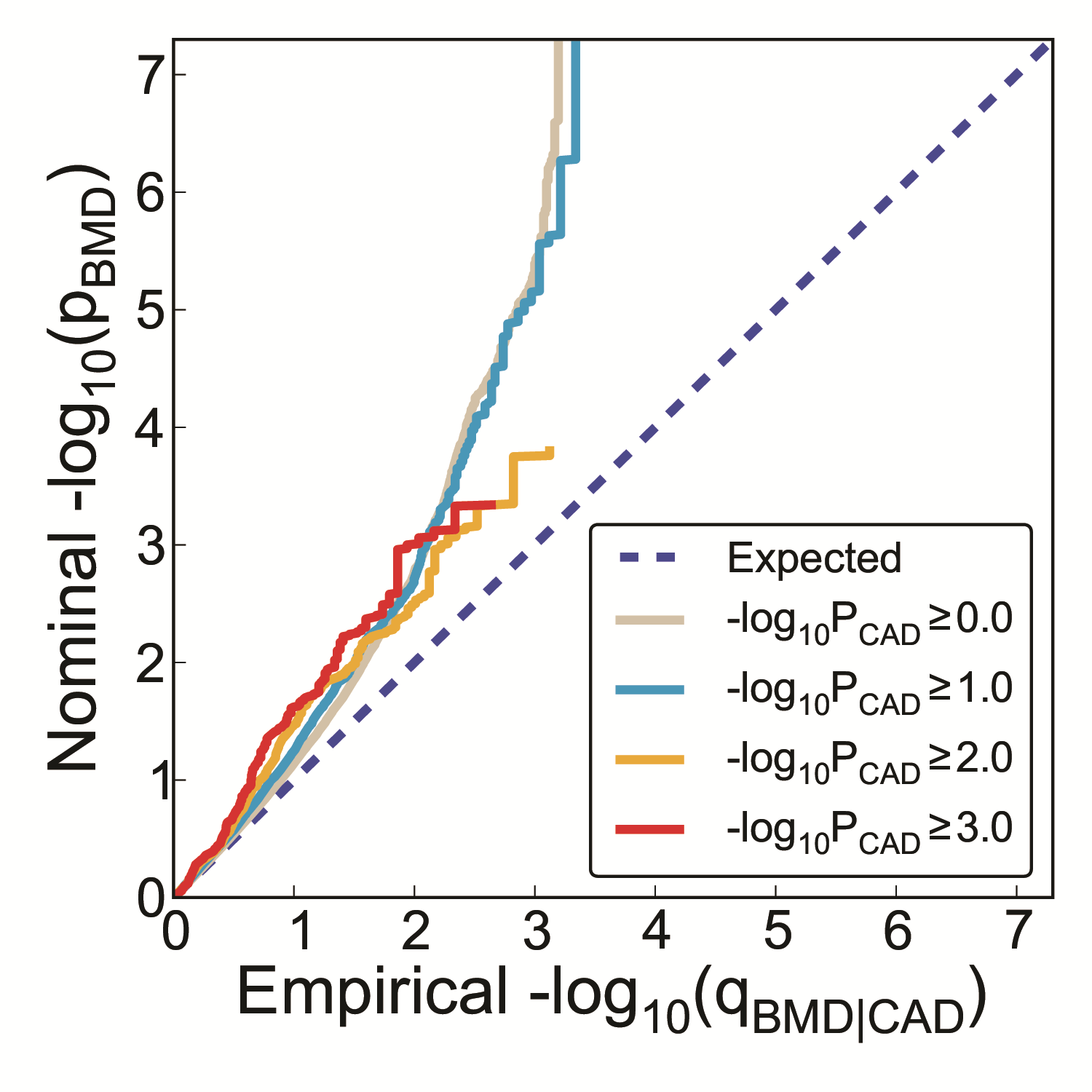


Conditional Q-Q plot of nominal versus empirical -log_10_ p-values (corrected for inflation) in bone mineral density (BMD, lumbar spine) below the standard GWAS threshold of p < 5x10^-8^ as a function of significance of association with Coronary Artery Disease (CAD) at the level of -log_10_(p) ≥ 0 (all SNPs), –log_10_(p) ≥ 1, –log_10_(p) ≥ 2, –log_10_(p) ≥ 3 corresponding to p ≤ 1, p ≤ 0.1, p ≤ 0.01, p ≤ 0.001, respectively. Dotted lines indicate the null-hypothesis.

### Supplementary Tables

### S1 Table. Summary data from all GWAS used in the current study

| **Disease/Trait** | | **N** | **# SNPs** | **Overlap (%)** | **Reference** |
| --- | --- | --- | --- | --- | --- |
| Bone Mineral Density (BMD) |  | 32,961 | 2,500,000 | - | Estrada, K. et al. Genome-wide meta-analysis identifies 56 bone mineral density loci and reveals 14 loci associated with risk of fracture. Nat Genet 2012;44:491-501. |
| Type 2 Diabetes (T2D) |  | 22,044 | 2,426,886 | 19.4 | Lango Allen, H. et al. Hundreds of variants clustered in genomic loci and biological pathways affect human height. Nature 467, 832-8 (2010). |
| Type 1 Diabetes (T1D) |  | 16,559 | 841,622 | 0 | Barrett, J.C. et al .Genome-wide association study and meta-analysis find that over 40 loci affect risk of type 1 diabetes. Nat Genet 2009, 41, 703-7. |
| Systolic Blood Pressure (SBP) |  | 203,056 | 2,382,073 | 7.9 | Ehret GB, Munroe PB, Rice KM, et al. Genetic variants in novel pathways influence blood pressure and cardiovascular disease risk. Nature 2011;478:103-9. |
| Diastolic Blood Pressure (DBP) |  | 203,056 | 2,382,073 | 7.9 |
| High Density Lipoprotein (HDL) |  | 96,598 | 2,508,370 | 10.8 | Teslovich TM, Musunuru K, Smith AV, et al. Biological, clinical and population relevance of 95 loci for blood lipids. Nature 2010;466:707-13. |
| Low Density Lipoprotein (LDL) |  | 99,900 | 2,508,375 | 10.5 |
| Triglycerides (TG) |  | 96,568 | 2,508,369 | 10.8 |
| Waist to hip ratio  (WHR) |  | 77,167 | 2,376,820 | 21.0 | Heid IM, Jackson AU, Randall JC, et al. Meta-analysis identifies 13 new loci associated with waist-hip ratio and reveals sexual dimorphism in the genetic basis of fat distribution. Nat Genet 2010;42:949-60. |

For more details, see also <http://www.genome.gov/gwastudies>

| **S2 Table. All identified loci associated with femoral neck BMD** | | | | | | | | |
| --- | --- | --- | --- | --- | --- | --- | --- | --- |
| **locus#** | **SNP** | **Map loc.** | **Gene Symbol** | **BMD**  **p-value** | **BMD FDR** | **Wald**  **stats** | **Min cond FDR** | **Driving phenotype** |
| 1 | rs10779702 | 1p36.23 | *RERE* | 7,78E-08 | **3,06E-04** | -5.26 | 1,60E-04 | HDL |
| 1 | rs894875 | 1p36.23 | *RERE* | 2,04E-08 | **8,99E-05** | -5.49 | 8,36E-05 | SBP |
| 1 | rs6678140 | 1p36.23 | *RERE* | 2,04E-08 | **8,99E-05** | -5.49 | 7,99E-05 | SBP |
| 2 | rs7521902* | 1p36.23-p35.1 | *WNT4** | 5,77E-08 | **2,52E-04** | 5.31 | 3,51E-04 | DBP |
| 3 | rs6690148 | 1p36 | *ZBTB40** | 5,14E-07 | **1,42E-03** | 4.92 | 1,32E-03 | HDL |
| 3 | rs10917209 | 1p36 | *ZBTB40** | 3,40E-07 | **9,66E-04** | 4.99 | 1,12E-03 | HDL |
| 3 | rs10917214 | 1p36 | *ZBTB40** | 3,60E-07 | **1,17E-03** | -4.98 | 1,41E-03 | LDL |
| 3 | rs12742784 | 1p36 | *ZBTB40** | 3,43E-17 | **6,36E-07** | -8.26 | 5,32E-07 | T2D |
| 3 | rs12029258 | 1p36 | *ZBTB40** | 6,96E-06 | 1,00E-02 | 4.4 | 6,39E-03 | SBP |
| 3 | rs12048810 | 1p36 | *ZBTB40** | 6,68E-06 | 1,00E-02 | 4.41 | 6,87E-03 | SBP |
| 4 | rs11810751 | 1p36 | *ZBTB40** | 2,20E-20 | **6,36E-07** | -9.06 | 2,67E-07 | HDL |
| 4 | rs12723796 | 1p36 | *ZBTB40** | 3,13E-16 | **6,36E-07** | -8 | 4,81E-07 | T2D |
| 4 | rs12568930 | 1p36 | *ZBTB40** | 5,89E-24 | **6,36E-07** | -9.89 | 5,32E-07 | T2D |
| 4 | rs11576345 | 1p36 | *ZBTB40** | 3,29E-07 | **9,66E-04** | -5 | 9,49E-04 | LDL |
| 4 | rs1320601 | 1p36 | *ZBTB40** | 3,39E-07 | **9,66E-04** | -5 | 8,30E-04 | LDL |
| 4 | rs7543680 | 1p36 | *ZBTB40** | 4,66E-16 | **6,36E-07** | -7.95 | 1,65E-07 | HDL |
| 4 | rs4394609 | 1p36 | *ZBTB40** | 1,38E-15 | **6,36E-07** | -7.82 | 5,06E-07 | T2D |
| 4 | rs4409621 | 1p36 | *ZBTB40** | 1,48E-15 | **6,36E-07** | -7.81 | 2,05E-07 | HDL |
| 5 | rs4655059 | 1p36 | *ZBTB40** | 2,55E-08 | **1,11E-04** | 5.46 | 8,41E-05 | SBP |
| 6 | rs12137389 | 1p32 | *TESK2* | 1,88E-06 | **4,15E-03** | 4.67 | 4,01E-03 | HDL |
| 7 | rs1430742 | 1p31.3 | *WLS/GNG12-AS1* | 1,01E-13 | **6,36E-07** | -7.29 | 2,41E-07 | SBP |
| 7 | rs2566755 | 1p31.3 | *WLS/GNG12-AS1* | 1,01E-13 | **6,36E-07** | -7.29 | 2,41E-07 | SBP |
| 7 | rs12407028* | 1p31.3 | *WLS/GNG12-AS1* | 7,26E-09 | **3,81E-05** | 5.67 | 4,50E-05 | SBP |
| 7 | rs2772300 | 1p31.3 | *WLS/GNG12-AS1* | 9,12E-10 | **5,58E-06** | -6 | 7,26E-06 | DBP |
| 8 | rs7554551 | 1p31.3 | *WLS/MIR1262/GNG12-AS1* | 3,69E-13 | **6,36E-07** | -7.12 | 2,41E-07 | SBP |
| 9 | rs11809524 | 1p21 | *COL11A1* | 8,21E-07 | **2,03E-03** | -4.83 | 1,34E-03 | SBP |
| 10 | rs681398 | 1q24.1 | *DNM3*/MIR3120* | 8,85E-06 | 1,20E-02 | -4.35 | 9,78E-03 | TG |
| 10 | rs479336* | 1q24.1 | *DNM3*/MIR3120* | 5,69E-08 | **2,52E-04** | -5.32 | 1,79E-04 | HDL |
| 11 | rs9309664 | 2p23 | *PPP1CB* | 7,55E-06 | 1,20E-02 | 4.39 | 8,22E-03 | HDL |
| 12 | rs7584262* | 2p21 | *PKDCC** | 7,84E-08 | **3,06E-04** | -5.26 | 4,88E-04 | LDL |
| 12 | rs2165239 | 2p21 | *PKDCC** | 2,66E-07 | **7,93E-04** | -5.04 | 9,04E-04 | T1D |
| 13 | rs17040773* | 2q12.1 | *ANAPC1** | 2,67E-06 | **4,96E-03** | 4.6 | 3,76E-03 | DBP |
| 14 | rs6710518 | 2q24-q31 | *GALNT3** | 8,70E-11 | **6,36E-07** | 6.35 | 6,18E-07 | SBP |
| 14 | rs1346004* | 2q24-q31 | *GALNT3** | 7,36E-11 | **6,36E-07** | 6.38 | 5,88E-07 | SBP |
| 15 | rs11675051 | 2q32.2 | *TMEM194B* | 1,46E-06 | **3,47E-03** | -4.72 | 1,56E-03 | SBP |
| 15 | rs3934784 | 2q32.2 | *TMEM194B* | 1,94E-06 | **4,15E-03** | -4.66 | 1,80E-03 | SBP |
| 15 | rs13005335 | 2q32.3-q33 | *NAB1* | 1,54E-06 | **3,47E-03** | -4.71 | 1,56E-03 | SBP |
| 16 | rs12995369 | 2q33.2 | *CDK15* | 1,07E-07 | **3,69E-04** | -5.2 | 2,80E-04 | SBP |
| 17 | rs7594560 | 2q33.3 | *METTL21A* | 3,42E-06 | **5,91E-03** | 4.55 | 3,74E-03 | HDL |
| 18 | rs416486 | 3p21 | *CTNNB1** | 4,57E-16 | **6,36E-07** | -7.96 | 2,41E-07 | SBP |
| 18 | rs1915925 | 3p21 | *CTNNB1** | 7,81E-06 | 1,20E-02 | 4.38 | 7,14E-03 | DBP |
| 18 | rs368006 | 3p21 | *CTNNB1** | 1,27E-14 | **6,36E-07** | 7.55 | 2,41E-07 | SBP |
| 18 | rs398993 | 3p21 | *CTNNB1** | 1,39E-14 | **6,36E-07** | 7.54 | 2,41E-07 | SBP |
| 18 | rs423170 | 3p21 | *CTNNB1** | 3,87E-16 | **6,36E-07** | 7.97 | 2,41E-07 | SBP |
| 18 | rs2024219 | 3p21 | *CTNNB1** | 4,63E-14 | **6,36E-07** | -7.39 | 2,41E-07 | SBP |
| 18 | rs7642431 | 3p21 | *CTNNB1** | 1,92E-12 | **6,36E-07** | -6.89 | 2,41E-07 | SBP |
| 18 | rs6599143 | 3p21 | *CTNNB1** | 8,53E-13 | **6,36E-07** | -7 | 2,41E-07 | SBP |
| 19 | rs11718013 | 3q13.31 | *KIAA2018** | 7,65E-06 | 1,20E-02 | -4.38 | 7,98E-03 | T1D |
| 19 | rs1026364* | 3q13.2 | *KIAA2018** | 1,22E-06 | **2,90E-03** | -4.75 | 2,52E-03 | SBP |
| 19 | rs12493635 | 3q13.2 | *KIAA2018** | 1,22E-06 | **2,90E-03** | -4.75 | 2,52E-03 | SBP |
| 20 | rs3755955* | 4p16.3 | *IDUA* | 2,21E-07 | **6,52E-04** | 5.07 | 6,22E-04 | DBP |
| 21 | rs1054627 | 4q21.1 | *IBSP* | 4,19E-10 | **2,35E-06** | 6.12 | 5,08E-06 | T2D |
| 21 | rs1471399 | 4q21.1 | *MEPE** | 1,17E-09 | **6,92E-06** | 5.96 | 6,62E-06 | LDL |
| 21 | rs1471403 | 4q21.1 | *MEPE** | 1,01E-09 | **5,58E-06** | 5.98 | 7,90E-06 | LDL |
| 21 | rs13130558 | 6p12 | *SPP1* | 1,21E-09 | **6,92E-06** | 5.95 | 1,00E-05 | SBP |
| 22 | rs17558396 | 5q14.3 | *MEF2C** | 4,10E-07 | **1,17E-03** | -4.96 | 7,69E-04 | SBP |
| 22 | rs700592 | 5q14.3 | *MEF2C** | 6,05E-07 | **1,70E-03** | 4.89 | 1,05E-03 | SBP |
| 22 | rs11958401 | 5q14.3 | *MEF2C** | 3,90E-06 | **7,05E-03** | 4.52 | 3,81E-03 | SBP |
| 22 | rs11952384 | 5q14.3 | *MEF2C** | 4,53E-09 | **2,48E-05** | 5.74 | 3,43E-05 | T2D |
| 22 | rs214137 | 5q14.3 | *MEF2C** | 3,48E-09 | **1,62E-05** | -5.79 | 3,43E-05 | T2D |
| 22 | rs169952 | 5q14.3 | *MEF2C** | 1,33E-11 | **6,36E-07** | -6.63 | 5,58E-07 | T2D |
| 22 | rs188515 | 5q14.3 | *MEF2C** | 1,55E-11 | **6,36E-07** | -6.6 | 5,58E-07 | T2D |
| 22 | rs6876387 | 5q14.3 | *MEF2C** | 1,08E-19 | **6,36E-07** | 8.89 | 5,06E-07 | T2D |
| 22 | rs6894139 | 5q14.3 | *MEF2C** | 2,99E-28 | **6,36E-07** | 10.79 | 5,51E-07 | LDL |
| 22 | rs1158464 | 5q14.3 | *MEF2C** | 3,56E-11 | **6,36E-07** | 6.48 | 2,41E-07 | SBP |
| 22 | rs10037512 | 5q14.3 | *MEF2C** | 4,27E-29 | **6,36E-07** | 10.97 | 5,87E-07 | T2D |
| 22 | rs1283614 | 5q14.3 | *MEF2C** | 3,28E-06 | **5,91E-03** | -4.56 | 3,10E-03 | SBP |
| 22 | rs1864180 | 5q14.3 | *MEF2C** | 2,05E-14 | **6,36E-07** | 7.49 | 4,21E-07 | T2D |
| 22 | rs10474292 | 5q14.3 | *MEF2C** | 2,75E-09 | **1,31E-05** | 5.82 | 1,43E-05 | SBP |
| 22 | rs13183402 | 5q14.3 | *MEF2C** | 3,85E-06 | **7,05E-03** | -4.52 | 5,83E-03 | SBP |
| 22 | rs7445369 | 5q14.3 | *MEF2C** | 5,79E-06 | 1,00E-02 | 4.44 | 8,66E-03 | DBP |
| 23 | rs4957742 | 5q21.2 | *RAB9BP1* | 2,98E-06 | **5,91E-03** | -4.58 | 6,27E-03 | DBP |
| 24 | rs1005886 | 6p22.2 | *CDKAL1** | 5,57E-06 | **8,40E-03** | 4.45 | 9,21E-03 | T1D |
| 24 | rs6456420 | 6p22.2 | *CDKAL1** | 5,57E-06 | **8,40E-03** | -4.45 | 9,16E-03 | TG |
| 24 | rs9466056* | 6p22.2 | *CDKAL1** | 9,01E-09 | **4,73E-05** | -5.63 | 6,89E-05 | LDL |
| 25 | rs10484759 | 6q22.32 | *CENPW* | 6,45E-06 | 1,00E-02 | 4.42 | 8,91E-03 | DBP |
| 25 | rs17563605 | 6q22.33 | *RSPO3* | 9,35E-07 | **2,43E-03** | 4.8 | 1,94E-03 | SBP |
| 25 | rs13204965* | 6q22.33 | *RSPO3* | 8,93E-07 | **2,43E-03** | 4.81 | 1,94E-03 | SBP |
| 26 | rs9479055 | 6q25.1 | *CCDC170** | 5,98E-11 | **6,36E-07** | -6.41 | 3,99E-07 | T1D |
| 26 | rs10872673 | 6q25.1 | *CCDC170** | 3,21E-12 | **6,36E-07** | -6.82 | 2,85E-07 | LDL |
| 26 | rs1856859 | 6q25.1 | *CCDC170** | 4,37E-08 | **1,69E-04** | 5.36 | 1,38E-04 | LDL |
| 26 | rs9479072 | 6q25.1 | *CCDC170** | 1,08E-13 | **6,36E-07** | -7.28 | 2,85E-07 | LDL |
| 26 | rs4869738 | 6q25.1 | *CCDC170** | 1,76E-06 | **3,47E-03** | -4.68 | 3,33E-03 | T1D |
| 26 | rs9479075 | 6q25.1 | *CCDC170** | 1,52E-15 | **6,36E-07** | -7.81 | 2,70E-07 | LDL |
| 26 | rs1871859 | 6q25.1 | *CCDC170** | 2,19E-09 | **1,06E-05** | 5.86 | 1,27E-05 | LDL |
| 26 | rs9478223 | 6q25.1 | *CCDC170** | 2,41E-06 | **4,96E-03** | 4.62 | 4,18E-03 | T2D |
| 26 | rs4869741 | 6q25.1 | *CCDC170** | 1,14E-14 | **6,36E-07** | 7.56 | 2,57E-07 | LDL |
| 26 | rs4869742* | 6q25.1 | *CCDC170** | 1,04E-14 | **6,36E-07** | 7.57 | 2,55E-07 | LDL |
| 26 | rs6925996 | 6q25.1 | *CCDC170** | 7,19E-13 | **6,36E-07** | 7.03 | 2,65E-07 | LDL |
| 26 | rs9383930 | 6q25.1 | *CCDC170** | 2,16E-09 | **1,06E-05** | 5.86 | 8,74E-06 | HDL |
| 26 | rs9397066 | 6q25.1 | *CCDC170** | 3,44E-09 | **1,62E-05** | 5.79 | 1,53E-05 | SBP |
| 26 | rs1340874 | 6q25.1 | *CCDC170** | 1,20E-08 | **5,87E-05** | 5.58 | 4,57E-05 | HDL |
| 26 | rs6929137 | 6q25.1 | *CCDC170** | 4,55E-12 | **6,36E-07** | 6.78 | 3,83E-07 | SBP |
| 26 | rs3734804 | 6q25.1 | *CCDC170** | 3,09E-14 | **6,36E-07** | 7.44 | 4,10E-07 | T2D |
| 26 | rs6904261 | 6q25.1 | *CCDC170** | 2,42E-09 | **1,31E-05** | 5.84 | 9,98E-06 | SBP |
| 26 | rs3734806 | 6q25.1 | *CCDC170** | 7,49E-12 | **6,36E-07** | 6.71 | 4,12E-07 | SBP |
| 26 | rs10872676 | 6q25.1 | *CCDC170** | 8,10E-14 | **6,36E-07** | 7.31 | 1,36E-07 | HDL |
| 26 | rs7752591 | 6q25.1 | *CCDC170** | 3,04E-13 | **6,36E-07** | 7.14 | 4,13E-07 | T2D |
| 26 | rs865898 | 6q25.1 | *CCDC170** | 4,33E-12 | **6,36E-07** | 6.78 | 4,13E-07 | T2D |
| 26 | rs712219 | 6q24-q27 | *ESR1* | 5,48E-13 | **6,36E-07** | 7.06 | 3,42E-07 | SBP |
| 26 | rs851970 | 6q24-q27 | *ESR1* | 9,63E-13 | **6,36E-07** | 6.99 | 3,05E-07 | SBP |
| 26 | rs980281 | 6q24-q27 | *ESR1* | 2,90E-07 | **9,66E-04** | 5.02 | 1,09E-03 | T1D |
| 26 | rs6557164 | 6q24-q27 | *ESR1* | 7,58E-06 | 1,20E-02 | 4.38 | 7,14E-03 | DBP |
| 26 | rs851993 | 6q24-q27 | *ESR1* | 1,62E-12 | **6,36E-07** | 6.92 | 4,31E-07 | T1D |
| 26 | rs3020333 | 6q24-q27 | *ESR1* | 3,49E-15 | **6,36E-07** | -7.71 | 1,31E-07 | HDL |
| 26 | rs2982570 | 6q24-q27 | *ESR1* | 2,23E-14 | **6,36E-07** | -7.48 | 1,30E-07 | HDL |
| 26 | rs851984 | 6q24-q27 | *ESR1* | 9,86E-13 | **6,36E-07** | -6.99 | 1,32E-07 | HDL |
| 26 | rs851983 | 6q24-q27 | *ESR1* | 1,29E-12 | **6,36E-07** | -6.95 | 1,34E-07 | HDL |
| 26 | rs851980 | 6q24-q27 | *ESR1* | 7,97E-08 | **3,06E-04** | -5.26 | 1,13E-04 | HDL |
| 26 | rs6899458 | 6q24-q27 | *ESR1* | 7,32E-06 | 1,20E-02 | -4.39 | 3,11E-03 | HDL |
| 26 | rs2982554 | 6q24-q27 | *ESR1* | 2,57E-11 | **6,36E-07** | -6.53 | 1,35E-07 | HDL |
| 26 | rs1999805 | 6q24-q27 | *ESR1* | 4,37E-11 | **6,36E-07** | -6.46 | 1,52E-07 | HDL |
| 26 | rs1124674 | 6q24-q27 | *ESR1* | 1,35E-08 | **5,87E-05** | -5.56 | 3,77E-05 | HDL |
| 26 | rs2504070 | 6q24-q27 | *ESR1* | 7,07E-06 | 1,00E-02 | -4.4 | 6,13E-03 | SBP |
| 26 | rs1890010 | 6q24-q27 | *ESR1* | 3,42E-08 | **1,37E-04** | -5.4 | 1,05E-04 | SBP |
| 26 | rs2504069 | 6q24-q27 | *ESR1* | 3,63E-08 | **1,69E-04** | -5.39 | 1,05E-04 | SBP |
| 26 | rs2504063 | 6q24-q27 | *ESR1* | 8,35E-09 | **3,81E-05** | -5.64 | 2,95E-05 | HDL |
| 27 | rs6583337 | 7p22.3 | *FAM20C* | 3,30E-06 | **5,91E-03** | 4.56 | 3,38E-03 | LDL |
| 28 | rs1721385 | 7p14.1 | *EPDR1* | 1,65E-06 | **3,47E-03** | -4.69 | 2,50E-03 | DBP |
| 28 | rs1717731 | 7p14.1 | *EPDR1* | 1,73E-06 | **3,47E-03** | -4.68 | 2,50E-03 | DBP |
| 28 | rs1524058 | 7p14-p13 | *STARD3NL** | 2,55E-06 | **4,96E-03** | -4.61 | 2,55E-03 | SBP |
| 28 | rs4576333 | 7p14-p13 | *STARD3NL** | 1,66E-06 | **3,47E-03** | -4.69 | 1,81E-03 | DBP |
| 29 | rs2282930 | 7p12.2 | *GRB10* | 5,20E-06 | **8,40E-03** | 4.46 | 7,20E-03 | TG |
| 30 | rs12154661 | 7q21.3 | *C7orf76* | 3,10E-06 | **5,91E-03** | 4.57 | 6,28E-03 | T1D |
| 31 | rs2724034 | 7q21.3 | *C7orf76* | 5,26E-06 | **8,40E-03** | 4.46 | 9,94E-03 | T2D |
| 32 | rs3094750 | 7q21.3 | *C7orf76* | 7,26E-12 | **6,36E-07** | 6.71 | 4,61E-07 | HDL |
| 32 | rs3113040 | 7q21.3 | *C7orf76* | 1,06E-11 | **6,36E-07** | 6.66 | 4,05E-07 | HDL |
| 32 | rs10953178 | 7q21.3 | *C7orf76* | 3,75E-11 | **6,36E-07** | -6.48 | 3,53E-07 | HDL |
| 32 | rs7781370 | 7q21.3 | *C7orf76* | 7,49E-21 | **6,36E-07** | -9.17 | 4,74E-07 | LDL |
| 32 | rs4132567 | 7q21.3 | *C7orf76* | 5,06E-06 | **8,40E-03** | -4.47 | 9,34E-03 | HDL |
| 32 | rs4427101 | 7q21.3 | *C7orf76* | 6,71E-21 | **6,36E-07** | -9.18 | 5,23E-07 | HDL |
| 32 | rs4296976 | 7q21.3 | *C7orf76* | 1,94E-08 | **8,99E-05** | -5.5 | 1,10E-04 | HDL |
| 32 | rs4566970 | 7q21.3 | *C7orf76* | 1,94E-08 | **8,99E-05** | -5.5 | 1,24E-04 | HDL |
| 32 | rs10464592 | 7q21.3 | *SHFM1* | 4,28E-10 | **2,35E-06** | 6.11 | 4,07E-06 | SBP |
| 32 | rs2272224 | 7q21.3 | *SHFM1* | 4,85E-07 | **1,42E-03** | -4.93 | 1,12E-03 | DBP |
| 32 | rs13310130 | 7q21.3 | *SHFM1* | 4,33E-09 | **2,00E-05** | 5.75 | 2,12E-05 | DBP |
| 32 | rs10499928 | 7q21.3 | *SHFM1* | 7,88E-06 | 1,20E-02 | 4.38 | 7,89E-03 | SBP |
| 33 | rs2908007 | 7q31 | *WNT16** | 1,60E-07 | **5,37E-04** | -5.13 | 6,17E-04 | HDL |
| 33 | rs3779381 | 7q31 | *WNT16** | 5,10E-13 | **6,36E-07** | -7.07 | 2,41E-07 | SBP |
| 33 | rs2908004 | 7q31 | *WNT16** | 3,79E-11 | **6,36E-07** | -6.48 | 4,47E-07 | SBP |
| 33 | rs3801382 | 7q22.1-q31.1 | *FAM3C* | 2,05E-14 | **6,36E-07** | -7.49 | 2,41E-07 | SBP |
| 33 | rs917727 | 7q22.1-q31.1 | *FAM3C* | 1,04E-14 | **6,36E-07** | -7.57 | 2,41E-07 | SBP |
| 33 | rs7776725 | 7q22.1-q31.1 | *FAM3C* | 1,59E-14 | **6,36E-07** | -7.52 | 2,41E-07 | SBP |
| 34 | rs7812088* | 7q36.1 | *ABCF2* | 7,25E-07 | **2,03E-03** | -4.85 | 1,44E-03 | HDL |
| 34 | rs7781265 | 7q35-q36 | *SMARCD3* | 8,16E-07 | **2,03E-03** | -4.83 | 1,61E-03 | HDL |
| 35 | rs1670357 | 7q36 | *PTPRN2/MIR595* | 1,73E-06 | **3,47E-03** | -4.68 | 1,90E-03 | SBP |
| 35 | rs1670346 | 7q36 | *PTPRN2/MIR595* | 1,73E-06 | **3,47E-03** | -4.68 | 1,80E-03 | SBP |
| 35 | rs1733125 | 7q36 | *PTPRN2/MIR595* | 1,93E-06 | **4,15E-03** | -4.66 | 2,13E-03 | SBP |
| 36 | rs13255886 | 8q13.3 | *LACTB2* | 2,42E-07 | **7,93E-04** | 5.06 | 6,22E-04 | DBP |
| 36 | rs7017914* | 8q13.3 | *XKR9* | 2,03E-07 | **6,52E-04** | 5.09 | 5,85E-04 | SBP |
| 36 | rs1596566 | 8q13.3 | *XKR9* | 2,42E-07 | **7,93E-04** | 5.06 | 6,22E-04 | DBP |
| 36 | rs6472551 | 8q13.3 | *XKR9* | 2,64E-07 | **7,93E-04** | 5.04 | 6,99E-04 | SBP |
| 37 | rs980299 | 8q13.3 | *EYA1* | 1,18E-07 | **4,45E-04** | 5.19 | 3,39E-04 | HDL |
| 38 | rs13272568 | 8q21.11 | *PKIA* | 1,29E-06 | **2,90E-03** | 4.74 | 2,52E-03 | SBP |
| 39 | rs16891598 | 8q24 | *TNFRSF11B** | 1,92E-06 | **4,15E-03** | 4.66 | 3,38E-03 | SBP |
| 39 | rs6651219 | 8q24 | *TNFRSF11B** | 2,07E-06 | **4,15E-03** | 4.65 | 3,38E-03 | SBP |
| 39 | rs16891617 | 8q24 | *TNFRSF11B** | 3,29E-06 | **5,91E-03** | 4.56 | 3,28E-03 | SBP |
| 39 | rs4407910 | 8q24 | *TNFRSF11B** | 3,63E-12 | **6,36E-07** | -6.81 | 3,26E-07 | T1D |
| 39 | rs4355801 | 8q24 | *TNFRSF11B** | 1,95E-12 | **6,36E-07** | -6.89 | 3,26E-07 | T1D |
| 39 | rs4876868 | 8q24 | *TNFRSF11B** | 2,82E-08 | **1,37E-04** | -5.44 | 1,91E-04 | HDL |
| 39 | rs11573871 | 8q24 | *TNFRSF11B** | 2,59E-06 | **4,96E-03** | -4.6 | 3,48E-03 | HDL |
| 39 | rs3102735 | 8q24 | *TNFRSF11B** | 2,94E-10 | **1,89E-06** | 6.17 | 2,83E-06 | SBP |
| 39 | rs12386806 | 8q24 | *TNFRSF11B** | 2,48E-10 | **1,52E-06** | 6.2 | 2,68E-06 | T2D |
| 39 | rs10505348 | 8q24 | *TNFRSF11B** | 5,84E-15 | **6,36E-07** | 7.65 | 3,26E-07 | T1D |
| 39 | rs1385499 | 8q24 | *TNFRSF11B** | 8,95E-16 | **6,36E-07** | 7.87 | 4,13E-07 | T2D |
| 39 | rs1564860 | 8q24 | *TNFRSF11B** | 9,57E-17 | **6,36E-07** | 8.14 | 4,81E-07 | T2D |
| 39 | rs1825511 | 8q24 | *TNFRSF11B** | 1,57E-08 | **7,27E-05** | 5.54 | 1,27E-04 | LDL |
| 39 | rs6469794 | 8q24 | *TNFRSF11B** | 3,96E-12 | **6,36E-07** | 6.8 | 3,26E-07 | T1D |
| 39 | rs7013203 | 8q24 | *TNFRSF11B** | 4,33E-12 | **6,36E-07** | 6.78 | 3,26E-07 | T1D |
| 39 | rs1586274 | 8q23-q24.1 | *COLEC10* | 1,87E-14 | **6,36E-07** | 7.5 | 5,27E-07 | T2D |
| 39 | rs2326193 | 8q23-q24.1 | *COLEC10* | 4,33E-12 | **6,36E-07** | 6.78 | 3,26E-07 | T1D |
| 39 | rs7016585 | 8q23-q24.1 | *COLEC10* | 2,87E-11 | **6,36E-07** | 6.52 | 3,26E-07 | T1D |
| 39 | rs1385509 | 8q23-q24.1 | *COLEC10* | 1,12E-06 | **2,43E-03** | 4.77 | 1,50E-03 | HDL |
| 40 | rs567960 | 9q31 | *KLF4* | 6,82E-06 | 1,00E-02 | 4.41 | 5,84E-03 | DBP |
| 40 | rs665556 | 9q31 | *KLF4* | 6,68E-06 | 1,00E-02 | 4.41 | 5,84E-03 | DBP |
| 41 | rs7466269 | 9q34.11 | *FUBP3** | 2,26E-08 | **1,11E-04** | 5.48 | 5,25E-05 | HDL |
| 42 | rs12262178 | 10q11.2 | *MBL2** | 4,63E-06 | **8,40E-03** | 4.49 | 6,56E-03 | DBP |
| 42 | rs12262251 | 10q11.2 | *MBL2** | 5,18E-06 | **8,40E-03** | 4.46 | 6,42E-03 | DBP |
| 42 | rs11003047 | 10q11.2 | *MBL2** | 4,76E-06 | **8,40E-03** | 4.48 | 6,24E-03 | DBP |
| 43 | rs1385162 | 11p15.3 | *SOX6** | 1,08E-15 | **6,36E-07** | 7.85 | 4,57E-07 | T2D |
| 43 | rs7117858 | 11p15.3 | *SOX6** | 2,95E-17 | **6,36E-07** | 8.27 | 4,37E-07 | T2D |
| 43 | rs9787942 | 11p15.3 | *SOX6** | 6,45E-17 | **6,36E-07** | 8.18 | 4,22E-07 | T2D |
| 43 | rs4757353 | 11p15.3 | *SOX6** | 1,18E-16 | **6,36E-07** | 8.11 | 4,22E-07 | T2D |
| 43 | rs10766280 | 11p15.3 | *SOX6** | 1,59E-16 | **6,36E-07** | 8.08 | 4,10E-07 | T2D |
| 43 | rs7933516 | 11p15.3 | *SOX6** | 3,37E-15 | **6,36E-07** | 7.71 | 4,21E-07 | T2D |
| 43 | rs16931831 | 11p15.3 | *SOX6** | 1,19E-16 | **6,36E-07** | -8.11 | 4,37E-07 | T2D |
| 44 | rs10832519 | 11p15.3 | *SOX6** | 4,28E-07 | **1,17E-03** | -4.95 | 6,97E-04 | HDL |
| 44 | rs11023718 | 11p15.3 | *SOX6** | 3,19E-07 | **9,66E-04** | -5.01 | 5,84E-04 | HDL |
| 45 | rs1949481 | 11p15.3 | *SOX6** | 3,10E-07 | **9,66E-04** | 5.01 | 7,17E-04 | DBP |
| 46 | rs12418348 | 11p15.3 | *SOX6** | 1,66E-06 | **3,47E-03** | 4.69 | 2,48E-03 | HDL |
| 46 | rs7924783 | 11p15.3 | *SOX6** | 1,66E-06 | **3,47E-03** | 4.69 | 2,15E-03 | HDL |
| 47 | rs2021807 | 11p14.1 | *DCDC5* | 5,03E-07 | **1,42E-03** | -4.92 | 1,56E-03 | DBP |
| 47 | rs911268 | 11p14.1 | *DCDC5* | 4,74E-07 | **1,42E-03** | -4.93 | 1,59E-03 | LDL |
| 47 | rs10767877 | 11p14.1 | *DCDC5* | 4,62E-07 | **1,42E-03** | 4.94 | 1,39E-03 | DBP |
| 47 | rs1028643 | 11p14.1 | *DCDC5* | 5,22E-07 | **1,42E-03** | 4.91 | 1,39E-03 | DBP |
| 47 | rs273567 | 11p14.1 | *DCDC5* | 5,96E-07 | **1,70E-03** | 4.89 | 1,83E-03 | DBP |
| 47 | rs273608 | 11p14.1 | *DCDC5* | 8,55E-07 | **2,03E-03** | 4.82 | 2,05E-03 | HDL |
| 48 | rs7932354* | 11p11.2 | *ARHGAP1** | 1,15E-08 | **5,87E-05** | 5.59 | 2,02E-05 | HDL |
| 48 | rs6485690 | 11p11.2 | *SNORD67* | 1,49E-07 | **5,37E-04** | 5.15 | 1,59E-04 | HDL |
| 49 | rs600231 | 11q13.1 | *MALAT1* | 7,75E-06 | 1,20E-02 | -4.38 | 7,60E-03 | SBP |
| 50 | rs608343 | 11q13.4 | *LRP5** | 3,84E-07 | **1,17E-03** | 4.97 | 1,12E-03 | SBP |
| 51 | rs7304170 | 12p11.22 | *KLHL42* | 1,04E-06 | **2,43E-03** | -4.78 | 2,96E-03 | T2D |
| 51 | rs258415 | 12p11.22 | *KLHL42* | 3,55E-08 | **1,69E-04** | -5.4 | 1,43E-04 | SBP |
| 52 | rs2016266* | 12q13.13 | *SP7** | 2,74E-07 | **7,93E-04** | 5.03 | 5,41E-04 | HDL |
| 52 | rs7310771 | 12q13 | *ATF7* | 7,44E-07 | **2,03E-03** | 4.85 | 8,24E-04 | LDL |
| 52 | rs10783588 | 12q13 | *ATF7* | 8,99E-07 | **2,43E-03** | 4.81 | 1,03E-03 | HDL |
| 52 | rs7970141 | 12q13 | *ATF7* | 7,49E-07 | **2,03E-03** | 4.85 | 1,10E-03 | HDL |
| 53 | rs11614913 | 12q13.13 | *MIR196A2* | 4,20E-08 | **1,69E-04** | 5.37 | 1,25E-04 | SBP |
| 54 | rs10746070 | 12q23.3 | *RIC8B* | 2,14E-06 | **4,15E-03** | -4.64 | 3,13E-03 | HDL |
| 54 | rs759603 | 12q23.3 | *RIC8B* | 2,14E-06 | **4,15E-03** | -4.64 | 3,13E-03 | HDL |
| 54 | rs6539289 | 12q23.3 | *RIC8B* | 2,27E-06 | **4,96E-03** | -4.63 | 3,31E-03 | HDL |
| 54 | rs1444581 | 12q23.3 | *RIC8B* | 2,27E-06 | **4,96E-03** | -4.63 | 3,46E-03 | T2D |
| 55 | rs912100 | 13q | *AKAP11** | 1,86E-05 | 2,46E-02 | 4.19 | 7,10E-03 | HDL |
| 55 | rs9533090* | 13q | *AKAP11** | 3,96E-11 | **6,36E-07** | 6.47 | 6,17E-07 | T2D |
| 55 | rs9594738 | 13q | *AKAP11** | 5,17E-11 | **6,36E-07** | 6.43 | 7,28E-07 | T2D |
| 55 | rs17638544 | 13q | *AKAP11** | 2,81E-06 | **4,96E-03** | -4.59 | 3,18E-03 | DBP |
| 55 | rs10507508 | 13q | *AKAP11** | 2,74E-06 | **4,96E-03** | -4.59 | 2,61E-03 | DBP |
| 56 | rs1286147 | 14q31-q32.1 | *RPS6KA5** | 7,47E-09 | **3,81E-05** | -5.66 | 5,16E-05 | T2D |
| 57 | rs3783394 | 14q32.3 | *MARK3** | 7,96E-07 | **2,03E-03** | 4.83 | 1,20E-03 | DBP |
| 57 | rs6575984 | 14q32.3 | *MARK3** | 8,99E-07 | **2,43E-03** | -4.81 | 9,64E-04 | HDL |
| 57 | rs11623869* | 14q32.3 | *MARK3** | 7,49E-07 | **2,03E-03** | 4.85 | 1,20E-03 | DBP |
| 57 | rs7152202 | 14q32.3 | *MARK3** | 7,49E-07 | **2,03E-03** | 4.85 | 1,20E-03 | DBP |
| 57 | rs7158144 | 14q32.3 | *MARK3** | 7,49E-07 | **2,03E-03** | 4.85 | 1,20E-03 | DBP |
| 57 | rs7158822 | 14q32.3 | *MARK3** | 7,49E-07 | **2,03E-03** | 4.85 | 1,20E-03 | DBP |
| 58 | rs4646 | 15q21 | *CYP19A1* | 5,53E-06 | **8,40E-03** | -4.45 | 4,90E-03 | SBP |
| 58 | rs7175531 | 15q21 | *CYP19A1* | 2,30E-06 | **4,96E-03** | -4.63 | 4,82E-03 | HDL |
| 58 | rs4775936 | 15q21 | *MIR4713* | 3,41E-06 | **5,91E-03** | -4.55 | 4,77E-03 | T1D |
| 58 | rs10851498 | 15q21 | *MIR4713* | 2,73E-06 | **4,96E-03** | -4.59 | 4,45E-03 | TG |
| 59 | rs9921222* | 16p13.3 | *AXIN1*/LUC7L* | 1,37E-07 | **4,45E-04** | 5.16 | 6,90E-04 | DBP |
| 60 | rs13336428* | 16p13.3 | *PTX4* | 1,60E-07 | **5,37E-04** | 5.13 | 8,55E-04 | T1D |
| 61 | rs3198697 | 16p13.11 | *PDXDC1* | 1,01E-05 | 1,44E-02 | 4.32 | 5,00E-03 | HDL |
| 62 | rs1566045* | 16q12.1 | *SALL1* | 1,76E-12 | **6,36E-07** | -6.91 | 3,26E-07 | T1D |
| 63 | rs10048146* | 16q24 | *FOXL1* | 6,95E-08 | **2,52E-04** | 5.28 | 2,92E-04 | HDL |
| 64 | rs4790881* | 17p13.3 | *SMG6** | 8,24E-09 | **3,81E-05** | -5.64 | 2,93E-05 | SBP |
| 64 | rs8077194 | 17p13.3 | *SMG6** | 1,22E-08 | **5,87E-05** | -5.58 | 3,63E-05 | SBP |
| 65 | rs1877632 | 17q12-q21 | *SOST** | 2,98E-09 | **1,62E-05** | -5.81 | 1,10E-05 | HDL |
| 66 | rs227580 | 17q21.31 | *C17orf53** | 1,95E-11 | **6,36E-07** | -6.57 | 2,41E-07 | SBP |
| 66 | rs227584* | 17q21.31 | *C17orf53** | 1,33E-11 | **6,36E-07** | -6.63 | 2,41E-07 | SBP |
| 66 | rs730228 | 17q21.31 | *C17orf53** | 2,11E-11 | **6,36E-07** | -6.56 | 3,53E-07 | HDL |
| 66 | rs7207464 | 17q21.31 | *ASB16* | 1,43E-11 | **6,36E-07** | -6.62 | 2,41E-07 | SBP |
| 66 | rs721769 | 17q21.31 | *ASB16-AS1* | 2,11E-11 | **6,36E-07** | -6.56 | 2,41E-07 | SBP |
| 67 | rs199533 | 17q21 | *NSF* | 5,77E-06 | 1,00E-02 | 4.44 | 5,84E-03 | DBP |
| 67 | rs199529 | 17q21 | *NSF* | 2,39E-06 | **4,96E-03** | 4.62 | 2,55E-03 | SBP |
| 68 | rs10491193 | 17q24.3 | *SOX9* | 9,72E-06 | 1,44E-02 | -4.33 | 8,54E-03 | HDL |
| 68 | rs12709255 | 17q24.3 | *SOX9* | 2,50E-08 | **1,11E-04** | 5.46 | 1,86E-04 | SBP |
| 68 | rs7217932* | 17q24.3 | *SOX9* | 1,88E-08 | **8,99E-05** | 5.51 | 1,26E-04 | T2D |
| 68 | rs11650567 | 17q24.3 | *SOX9* | 2,17E-08 | **8,99E-05** | 5.48 | 1,52E-04 | SBP |
| 69 | rs4796995* | 18p11.21 | *FAM210A** | 1,96E-06 | **4,15E-03** | 4.66 | 2,26E-03 | SBP |
| 70 | rs2957153 | 18q22.1 | *TNFRSF11A** | 2,54E-06 | **4,96E-03** | -4.61 | 3,07E-03 | DBP |
| 71 | rs11659752 | 18q23 | *NFATC1* | 4,79E-06 | **8,40E-03** | -4.48 | 8,20E-03 | LDL |
| 71 | rs8090312 | 18q23 | *NFATC1* | 4,54E-06 | **8,40E-03** | -4.49 | 6,40E-03 | T1D |
| 71 | rs11660128 | 18q23 | *NFATC1* | 3,42E-06 | **5,91E-03** | -4.55 | 7,17E-03 | TG |
| 72 | rs3760891 | 19q13.12 | *GPATCH1** | 2,78E-06 | **4,96E-03** | -4.59 | 4,75E-03 | LDL |
| 72 | rs2287679 | 19q13.12 | *GPATCH1** | 2,63E-06 | **4,96E-03** | -4.6 | 4,46E-03 | LDL |
| 72 | rs10416265 | 19q13.12 | *GPATCH1** | 2,47E-06 | **4,96E-03** | -4.61 | 4,07E-03 | T1D |
| 73 | rs6514116 | 20p12.1-p11.23 | *JAG1** | 5,29E-08 | **2,07E-04** | 5.33 | 3,06E-04 | SBP |
| 73 | rs6040061 | 20p12.1-p11.23 | *JAG1** | 5,20E-08 | **2,07E-04** | 5.33 | 2,71E-04 | T1D |
| 74 | rs2267004 | 22q11.2 | *RTDR1/GNAZ* | 3,21E-06 | **5,91E-03** | 4.56 | 7,17E-03 | TG |
| 74 | rs1051875 | 22q11.2 | *RTDR1/GNAZ* | 4,57E-06 | **8,40E-03** | 4.49 | 5,95E-03 | TG |
| 74 | rs756632 | 22q11.2 | *RTDR1/GNAZ* | 3,39E-06 | **5,91E-03** | -4.55 | 4,75E-03 | HDL |
| 74 | rs13055979 | 22q11.2 | *RTDR1/GNAZ* | 6,70E-06 | 1,00E-02 | 4.41 | 5,84E-03 | DBP |
| 74 | rs4820539 | 22q11.2 | *RAB36* | 3,06E-06 | **5,91E-03** | 4.57 | 7,44E-03 | HDL |

Independent complex or single gene loci (LD-r^2^ < 0.2) with SNP(s) with a conditional FDR (condFDR) < 0.01 in bone mineral density (BMD, femoral neck) given the association in other phenotypes. We defined the most significant BMD SNP in each LD block based on the minimum condFDR (min cond FDR) for each phenotype. The second phenotype which provides the minimal FDR signal (Driving phenotype) is listed. All loci with SNPs with condFDR < 0.01 were used to define the number of the loci. The following abbreviations were used: type 1 diabetes (T1D), type 2 diabetes (T2D), systolic blood pressure (SBP), diastolic blood pressure (DBP), high density lipoprotein (HDL), low density lipoprotein (LDL), triglycerides (TG), waist hip ratio (WHR), chromosome location (Map Loc.). Shaded r values represent nominally significant (p<0.05) Pearson correlations (age and BMI adjusted FN BMD vs. Affymetrix signal values). SNPs and Genes previously reported to associate with BMD are marked with stars (*). BMD FDR values < 0.01 are in bold. Wald stats: z-score transformed from p values.

| **S3 Table. Identified loci containing novel SNPs or genes associated with lumbar spine BMD** | | | | | | | | |
| --- | --- | --- | --- | --- | --- | --- | --- | --- |
| **Locus#** | **SNP** | **Map loc.** | **Gene symbol** | **BMD**  **p-value** | **BMD FDR** | **Wald stats** | **min cond FDR** | **Driving phenotype** |
| 1 | rs12079653 | 1p36.23 | *RERE* | 2,56E-05 | 2,55E-02 | -4.11 | 9,54E-03 | LDL |
| 2 | rs12136689 | 1p36.23 | *RERE* | 9,75E-06 | 1,19E-02 | 4.32 | 6,88E-03 | DBP |
| 7 | rs1889830 | 1p32-p31 | *MACF1* | 1,18E-05 | 1,44E-02 | 4.28 | 4,91E-03 | HDL |
| 7 | rs2275187 | 1p32-p31 | *MACF1* | 1,01E-05 | 1,19E-02 | 4.31 | 4,91E-03 | HDL |
| 8 | rs2566784 | 1p31.3 | *WLS/GNG12-AS1* | 6,67E-08 | **1,72E-04** | -5.27 | 1,62E-04 | SBP |
| 10 | rs7554551 | 1p31.3 | *WLS/MIR1262/GNG12-AS1* | 1,07E-15 | **5,10E-07** | -7.83 | 3,30E-07 | SBP |
| 11 | rs2273368 | 1p13 | *WNT2B* | 1,22E-05 | 1,44E-02 | -4.27 | 4,30E-03 | DBP |
| 11 | rs17030613 | 1p13.2 | *CAPZA1* | 1,14E-05 | 1,44E-02 | -4.28 | 4,30E-03 | DBP |
| 12 | rs12120297 | 1q41 | *SUSD4* | 1,73E-06 | **2,47E-03** | 4.67 | 2,23E-03 | LDL |
| 13 | rs780110 | 2p23.3 | *IFT172* | 3,83E-05 | 3,64E-02 | -4.02 | 7,67E-03 | TG |
| 14 | rs4389358 | 2p16.2 | *C2orf73* | 2,26E-05 | 2,55E-02 | 4.14 | 6,60E-03 | HDL |
| 16 | rs17049689 | 2p16.1 | *FANCL* | 1,40E-05 | 1,44E-02 | -4.24 | 5,79E-03 | DBP |
| 19 | rs7636399 | 3p23 | *SUSD5* | 4,11E-06 | **5,45E-03** | 4.5 | 4,68E-03 | LDL |
| 19 | rs9861369 | 3p23 | *SUSD5* | 5,13E-06 | **6,62E-03** | 4.45 | 3,40E-03 | DBP |
| 19 | rs7630970 | 3p23 | *SUSD5* | 4,93E-06 | **6,62E-03** | 4.46 | 3,11E-03 | DBP |
| 21 | rs6599179 | 3p22.1 | *ULK4* | 6,83E-06 | **8,05E-03** | 4.39 | 3,90E-03 | WHR |
| 21 | rs900569 | 3p22.1 | *ULK4* | 1,81E-05 | 2,11E-02 | 4.18 | 7,60E-03 | DBP |
| 21 | rs9856088 | 3p22.1 | *ULK4* | 5,94E-06 | **8,05E-03** | 4.42 | 3,18E-03 | WHR |
| 21 | rs1717017 | 3p22.1 | *ULK4* | 8,06E-06 | **9,79E-03** | 4.36 | 3,90E-03 | WHR |
| 21 | rs1716655 | 3p22.1 | *ULK4* | 9,12E-06 | 1,19E-02 | -4.33 | 2,95E-03 | DBP |
| 21 | rs9838537 | 3p22.1 | *ULK4* | 2,73E-06 | **3,69E-03** | -4.58 | 1,30E-03 | WHR |
| 22 | rs11720264 | 3p21.31 | *BSN-AS2* | 1,29E-05 | 1,44E-02 | -4.26 | 5,12E-03 | WHR |
| 22 | rs11919311 | 3p21.31 | *BSN* | 3,51E-06 | **4,49E-03** | -4.53 | 1,88E-03 | SBP |
| 22 | rs1060962 | 3p21.31 | *BSN* | 4,41E-06 | **5,45E-03** | -4.48 | 2,33E-03 | SBP |
| 22 | rs4855881 | 3p21 | *APEH* | 3,93E-06 | **5,45E-03** | -4.5 | 1,88E-03 | SBP |
| 24 | rs6787138 | 3q27 | *ATP11B* | 8,66E-06 | **9,79E-03** | -4.34 | 3,90E-03 | WHR |
| 25 | rs6599389 | 4p16.3 | *TMEM175* | 1,30E-05 | 1,44E-02 | 4.25 | 4,28E-03 | WHR |
| 27 | rs11729056 | 4q13.3 | *AREG* | 1,33E-05 | 1,44E-02 | -4.25 | 5,18E-03 | DBP |
| 28 | rs11942753 | 4q21.3 | *DSPP* | 1,31E-05 | 1,44E-02 | 4.25 | 5,83E-03 | WHR |
| 30 | rs4862810 | 4q35.2 | *ZFP42* | 6,97E-06 | **8,05E-03** | -4.39 | 6,68E-03 | T2D |
| 31 | rs3822469 | 5p13 | *SLC1A3* | 8,23E-06 | **9,79E-03** | -4.35 | 6,14E-03 | DBP |
| 36 | rs6465508 | 7q21.3 | *C7orf76* | 3,17E-16 | **5,10E-07** | -7.97 | 2,51E-07 | LDL |
| 36 | rs10808100 | 7q21.3 | *C7orf76* | 3,48E-16 | **5,10E-07** | -7.96 | 2,74E-07 | LDL |
| 36 | rs7781370 | 7q21.3 | *C7orf76* | 3,81E-16 | **5,10E-07** | -7.95 | 1,91E-07 | LDL |
| 36 | rs4370463 | 7q21.3 | *C7orf76* | 3,81E-16 | **5,10E-07** | -7.95 | 2,30E-07 | LDL |
| 36 | rs10085588 | 7q21.3 | *C7orf76* | 2,54E-15 | **5,10E-07** | -7.72 | 2,51E-07 | LDL |
| 36 | rs6971293 | 7q21.3 | *C7orf76* | 3,48E-16 | **5,10E-07** | -7.96 | 2,51E-07 | LDL |
| 36 | rs2272224 | 7q21.3 | *SHFM1* | 1,15E-08 | **4,23E-05** | -5.57 | 3,73E-05 | T1D |
| 36 | rs2922927 | 7q21.3 | *SHFM1* | 1,31E-06 | **2,00E-03** | -4.72 | 1,27E-03 | LDL |
| 36 | rs7786855 | 7q21.3 | *SHFM1* | 2,47E-06 | **3,69E-03** | 4.6 | 2,26E-03 | DBP |
| 38 | rs10274324 | 7q31.31 | *CPED1* | 2,43E-06 | **3,69E-03** | -4.6 | 1,25E-03 | WHR |
| 38 | rs798917 | 7q31.31 | *CPED1* | 2,05E-06 | **3,02E-03** | -4.63 | 1,25E-03 | WHR |
| 38 | rs798915 | 7q31.31 | *CPED1* | 1,94E-06 | **3,02E-03** | -4.64 | 1,25E-03 | WHR |
| 38 | rs2691032 | 7q31.31 | *CPED1* | 1,74E-06 | **2,47E-03** | -4.67 | 1,25E-03 | WHR |
| 38 | rs2536150 | 7q31.31 | *CPED1* | 2,16E-06 | **3,02E-03** | 4.62 | 2,09E-03 | LDL |
| 41 | rs980695 | 8q23-q24.1 | *COLEC10* | 2,07E-06 | **3,02E-03** | -4.63 | 3,54E-03 | LDL |
| 42 | rs10956415 | 8q24 | *MIR1208* | 5,05E-06 | **6,62E-03** | -4.45 | 6,49E-03 | WHR |
| 43 | rs592203 | 9p24.1-p23 | *PTPRD/SNORD27* | 1,93E-05 | **2,11E-02** | 4.17 | 8,12E-03 | SBP |
| 44 | rs2388855 | 10p15 | *KLF6* | 3,56E-06 | **5,45E-03** | -4.52 | 3,80E-03 | LDL |
| 47 | rs4350264 | 10q21.3-q22.1 | *SLC25A16* | 1,01E-05 | 1,19E-02 | 4.31 | 3,98E-03 | LDL |
| 49 | rs11602954 | 11p15.5 | *BET1L* | 1,38E-07 | **2,99E-04** | -5.14 | 2,06E-04 | T1D |
| 53 | rs4514364 | 11p14-p13 | *LGR4* | 3,02E-06 | **4,49E-03** | 4.56 | 1,51E-03 | SBP |
| 53 | rs10767646 | 11p14.1 | *BDNF-AS* | 3,42E-06 | **4,49E-03** | 4.53 | 2,43E-03 | HDL |
| 55 | rs10160701 | 11p11.2 | *AMBRA1/HARBI1* | 5,47E-05 | 4,32E-02 | -3.94 | 9,82E-03 | HDL |
| 55 | rs2306029 | 11p11.2 | *LRP4-AS1/LRP4* | 1,31E-06 | **2,00E-03** | 4.72 | 7,96E-04 | SBP |
| 56 | rs7119750 | 11q13 | *RELA* | 1,08E-06 | **1,61E-03** | 4.76 | 6,39E-04 | SBP |
| 56 | rs7101916 | 11q13 | *RELA* | 5,54E-07 | **8,53E-04** | 4.89 | 3,29E-04 | SBP |
| 56 | rs12421691 | 11q13 | *KAT5* | 6,29E-07 | **1,05E-03** | 4.86 | 4,09E-04 | SBP |
| 58 | rs549932 | 11q14.2 | *TMEM135* | 1,12E-05 | 1,44E-02 | 4.29 | 6,04E-03 | T1D |
| 60 | rs3730071 | 12q12-q13 | *ADCY6/MIR4701* | 2,08E-06 | **3,02E-03** | 4.63 | 1,25E-03 | WHR |
| 61 | rs7311091 | 12q13 | *DDN* | 4,76E-06 | **6,62E-03** | -4.46 | 3,30E-03 | DBP |
| 64 | rs11614913 | 12q13.13 | *MIR196A2* | 1,20E-11 | **5,10E-07** | 6.62 | 1,70E-07 | WHR |
| 64 | rs3803042 | 12q13.13 | *MIR196A2* | 1,20E-11 | **5,10E-07** | 6.62 | 1,70E-07 | WHR |
| 64 | rs894737 | 12q13.13 | *HOXC6/HOXC4/HOXC5* | 2,08E-11 | **5,10E-07** | 6.54 | 1,70E-07 | WHR |
| 64 | rs754133 | 12q13.13 | *HOXC6/HOXC4/HOXC5* | 4,55E-11 | **5,10E-07** | 6.43 | 2,10E-07 | WHR |
| 66 | rs1047796 | 12q21.31 | *MLXIP* | 2,45E-05 | 2,55E-02 | 4.12 | 9,23E-03 | DBP |
| 71 | rs12438366 | 15q21-q22 | *SMAD3* | 1,03E-05 | 1,19E-02 | -4.3 | 7,68E-03 | DBP |
| 72 | rs7173826 | 15q22.33-q23 | *AAGAB* | 1,55E-05 | 1,75E-02 | -4.22 | 6,27E-03 | DBP |
| 79 | rs7350980 | 17q21.31 | *KANSL1* | 6,31E-08 | **1,72E-04** | 5.28 | 1,10E-04 | T1D |
| 79 | rs7221390 | 17q21.31 | *KANSL1* | 6,31E-08 | **1,72E-04** | 5.28 | 1,10E-04 | T1D |
| 79 | rs9303525 | 17q21.31 | *KANSL1* | 5,48E-08 | **1,42E-04** | 5.3 | 1,40E-04 | SBP |
| 79 | rs2696689 | 17q21.31 | *KANSL1-AS1* | 1,70E-07 | **3,53E-04** | 5.1 | 1,48E-04 | WHR |
| 80 | rs4794031 | 17q21.32 | *ZNF652* | 8,59E-06 | **9,79E-03** | 4.34 | 4,30E-03 | DBP |
| 80 | rs3179840 | 17q21.32 | *ZNF652* | 1,26E-05 | 1,44E-02 | 4.26 | 4,91E-03 | HDL |
| 80 | rs2906093 | 17q21.32 | *ZNF652* | 1,60E-05 | 1,75E-02 | 4.21 | 6,10E-03 | LDL |
| 81 | rs7226305 | 17q22 | *KIF2B* | 7,24E-07 | **1,30E-03** | -4.84 | 4,49E-04 | DBP |
| 81 | rs17730919 | 17q22 | *KIF2B* | 3,33E-06 | **4,49E-03** | 4.54 | 2,55E-03 | DBP |
| 82 | rs12601958 | 17q24.2 | *CEP112* | 5,42E-05 | 4,32E-02 | -3.94 | 8,74E-03 | TG |
| 83 | rs622924 | 18q21.33 | *PIGN* | 2,83E-06 | **4,49E-03** | -4.57 | 2,91E-03 | T2D |
| 83 | rs583339 | 18q21.33 | *PIGN* | 3,02E-06 | **4,49E-03** | -4.56 | 2,91E-03 | T2D |
| 87 | rs7227107 | 18q23 | *NFATC1* | 7,21E-06 | **9,79E-03** | -4.38 | 4,16E-03 | LDL |
| 89 | rs3760846 | 19q13.32 | *ERCC1* | 6,12E-06 | **8,05E-03** | 4.41 | 2,64E-03 | LDL |
| 90 | rs4813035 | 20p12 | *SLX4IP* | 2,35E-05 | 2,55E-02 | -4.13 | 7,60E-03 | DBP |
| 94 | rs17404303 | 20q13 | *BMP7* | 5,43E-06 | **6,62E-03** | 4.44 | 3,25E-03 | T1D |
| 95 | rs4817775 | 21 | *CBR3-AS1* | 8,17E-07 | **1,30E-03** | 4.81 | 5,12E-04 | SBP |
| Independent complex or single gene loci (LD-r2 < 0.2) with SNP(s) with a conditional FDR (condFDR) < 0.01 in bone mineral density (BMD, lumbar spine) given the association in other phenotypes. We defined the most significant BMD SNP in each LD block based on the minimum condFDR (min condFDR) for each phenotype. The second phenotype which provides the minimal FDR signal (driving phenotype) is listed. All loci with SNPs with condFDR < 0.01 were used to define the number of the loci. The following abbreviations were used: type 1 diabetes (T1D), type 2 diabetes (T2D), systolic blood pressure (SBP), diastolic blood pressure (DBP), high density lipoprotein (HDL), low density lipoprotein (LDL), triglycerides (TG), waist hip ratio (WHR), chromosome location (Map Loc.). Shaded r values represent nominally significant (p<0.05) Pearson correlations (age and BMI adjusted LS BMD vs Affymetrix signal values). Wald stats: z-score transformed from p values. | | | | | | | | |

| **S4 Table. Identified loci containing known SNPs or genes associated with lumbar spine BMD** | | | | | | | | |
| --- | --- | --- | --- | --- | --- | --- | --- | --- |
| **Locus#** | **SNP** | **Map loc.** | **Gene symbol** | **BMD**  **p-value** | **BMD FDR** | **Wald**  **stats** | **min cond FDR** | **Driving phenotype** |
| 3 | rs7521902* | 1p36.23-p35.1 | *WNT4** | 5,49E-07 | **8,53E-04** | 4.89 | 1,20E-03 | SBP |
| 3 | rs1014985 | 1p36.23-p35.1 | *WNT4** | 2,27E-07 | **4,89E-04** | -5.05 | 5,36E-04 | WHR |
| 3 | rs2982285 | 1p36.23-p35.1 | *WNT4** | 3,28E-06 | **4,49E-03** | -4.54 | 4,19E-03 | WHR |
| 3 | rs12030840 | 1p36.23-p35.1 | *WNT4** | 2,19E-09 | **7,83E-06** | 5.84 | 1,39E-05 | T2D |
| 3 | rs932371 | 1p36.23-p35.1 | *WNT4** | 1,92E-09 | **7,83E-06** | 5.86 | 1,39E-05 | T2D |
| 3 | rs2473236 | 1p36.23-p35.1 | *WNT4** | 4,89E-06 | **6,62E-03** | -4.46 | 3,97E-03 | WHR |
| 4 | rs4598465 | 1p36 | *ZBTB40** | 1,02E-06 | **1,61E-03** | 4.77 | 7,37E-04 | WHR |
| 4 | rs10917209 | 1p36 | *ZBTB40** | 1,39E-06 | **2,00E-03** | 4.71 | 1,37E-03 | LDL |
| 4 | rs10917214 | 1p36 | *ZBTB40** | 2,79E-07 | **4,89E-04** | -5.01 | 5,35E-04 | LDL |
| 4 | rs4655048 | 1p36 | *ZBTB40** | 6,94E-08 | **1,72E-04** | 5.26 | 2,49E-04 | HDL |
| 4 | rs1316342 | 1p36 | *ZBTB40** | 1,28E-06 | **2,00E-03** | 4.73 | 1,81E-03 | LDL |
| 4 | rs10753536 | 1p36 | *ZBTB40** | 1,93E-07 | **4,15E-04** | -5.08 | 2,13E-04 | WHR |
| 4 | rs12029258 | 1p36 | *ZBTB40** | 3,46E-07 | **5,82E-04** | 4.97 | 3,28E-04 | SBP |
| 4 | rs11810369 | 1p36 | *ZBTB40** | 2,08E-13 | **5,10E-07** | -7.17 | 2,50E-07 | HDL |
| 5 | rs12723796 | 1p36 | *ZBTB40** | 3,77E-17 | **5,10E-07** | -8.22 | 3,58E-07 | T2D |
| 5 | rs12568930 | 1p36 | *ZBTB40** | 1,26E-21 | **5,10E-07** | -9.32 | 3,55E-07 | TG |
| 5 | rs10493013 | 1p36 | *ZBTB40** | 9,65E-22 | **5,10E-07** | -9.35 | 3,56E-07 | LDL |
| 5 | rs7543680 | 1p36 | *ZBTB40** | 1,72E-11 | **5,10E-07** | -6.57 | 1,44E-07 | HDL |
| 5 | rs4394609 | 1p36 | *ZBTB40** | 6,32E-14 | **5,10E-07** | -7.32 | 3,29E-07 | TG |
| 5 | rs10799749 | 1p36 | *ZBTB40** | 1,06E-07 | **2,51E-04** | 5.19 | 1,80E-04 | SBP |
| 6 | rs4655059 | 1p36 | *ZBTB40** | 9,97E-08 | **2,51E-04** | 5.2 | 1,69E-04 | SBP |
| 9 | rs1367447 | 1p31.3 | *WLS/GNG12-AS1* | 7,60E-15 | **5,10E-07** | -7.59 | 3,30E-07 | SBP |
| 9 | rs1430742 | 1p31.3 | *WLS/GNG12-AS1* | 3,29E-19 | **5,10E-07** | -8.74 | 3,30E-07 | SBP |
| 9 | rs2566755 | 1p31.3 | *WLS/GNG12-AS1* | 3,02E-19 | **5,10E-07** | -8.75 | 3,30E-07 | SBP |
| 9 | rs2195682 | 1p31.3 | *WLS/GNG12-AS1* | 1,21E-20 | **5,10E-07** | 9.09 | 3,18E-07 | TG |
| 9 | rs4233320 | 1p31.3 | *WLS/GNG12-AS1* | 4,45E-20 | **5,10E-07** | 8.96 | 3,18E-07 | TG |
| 9 | rs891528 | 1p31.3 | *WLS/GNG12-AS1* | 4,88E-13 | **5,10E-07** | -7.05 | 3,66E-07 | DBP |
| 9 | rs944082 | 1p31.3 | *WLS/GNG12-AS1* | 3,22E-16 | **5,10E-07** | -7.97 | 3,98E-07 | T2D |
| 9 | rs12407028* | 1p31.3 | *WLS/GNG12-AS1* | 8,39E-21 | **5,10E-07** | 9.13 | 3,38E-07 | TG |
| 9 | rs12568456 | 1p31.3 | *WLS/MIR1262/GNG12-AS1* | 6,67E-17 | **5,10E-07** | -8.15 | 3,29E-07 | DBP |
| 15 | rs6734097 | 2p21 | *SPTBN1** | 2,74E-12 | **5,10E-07** | 6.82 | 3,64E-07 | TG |
| 15 | rs7607093 | 2p21 | *SPTBN1** | 3,21E-12 | **5,10E-07** | 6.8 | 4,36E-07 | TG |
| 15 | rs6752877 | 2p21 | *SPTBN1** | 2,00E-12 | **5,10E-07** | 6.87 | 3,97E-07 | TG |
| 15 | rs11898505 | 2p21 | *SPTBN1** | 4,06E-12 | **5,10E-07** | 6.77 | 4,36E-07 | TG |
| 17 | rs1878526* | 2q14.1 | *INSIG2** | 4,33E-06 | **5,45E-03** | -4.48 | 3,15E-03 | LDL |
| 17 | rs4594452 | 2q14.1 | *INSIG2** | 4,80E-06 | **6,62E-03** | -4.46 | 6,13E-03 | TG |
| 17 | rs12621455 | 2q14.1 | *INSIG2** | 5,43E-06 | **6,62E-03** | -4.44 | 4,93E-03 | T2D |
| 17 | rs7587150 | 2q14.1 | *INSIG2** | 6,56E-06 | **8,05E-03** | -4.4 | 6,30E-03 | T2D |
| 17 | rs4073566 | 2q14.1 | *INSIG2** | 5,10E-06 | **6,62E-03** | 4.45 | 5,74E-03 | TG |
| 18 | rs6710388 | 2q24-q31 | *GALNT3** | 8,01E-09 | **2,80E-05** | 5.63 | 2,57E-05 | SBP |
| 18 | rs1346004* | 2q24-q31 | *GALNT3** | 5,61E-09 | **1,84E-05** | 5.69 | 1,85E-05 | SBP |
| 20 | rs416486 | 3p21 | *CTNNB1** | 2,29E-11 | **5,10E-07** | -6.53 | 3,26E-07 | LDL |
| 20 | rs11717807 | 3p21 | *CTNNB1** | 2,29E-11 | **5,10E-07** | -6.53 | 3,26E-07 | LDL |
| 20 | rs422623 | 3p21 | *CTNNB1** | 1,95E-11 | **5,10E-07** | -6.55 | 3,26E-07 | LDL |
| 20 | rs385905 | 3p21 | *CTNNB1** | 2,49E-11 | **5,10E-07** | -6.51 | 2,74E-07 | LDL |
| 20 | rs428510 | 3p21 | *CTNNB1** | 2,29E-11 | **5,10E-07** | -6.53 | 3,26E-07 | LDL |
| 20 | rs368006 | 3p21 | *CTNNB1** | 1,78E-11 | **5,10E-07** | 6.56 | 2,30E-07 | LDL |
| 23 | rs7621699 | 3q25.31 | *LEKR1** | 9,23E-06 | **1,19E-02** | 4.33 | 3,83E-03 | WHR |
| 23 | rs16826948 | 3q25.31 | *LEKR1** | 1,11E-05 | **1,19E-02** | 4.29 | 5,65E-03 | WHR |
| 26 | rs3755955* | 4p16.3 | *IDUA** | 6,62E-08 | **1,72E-04** | 5.27 | 1,79E-04 | WHR |
| 29 | rs2904180 | 4q21.1 | *MEPE** | 3,87E-09 | **1,48E-05** | 5.75 | 1,81E-05 | SBP |
| 29 | rs13117929 | 4q21.1 | *MEPE** | 4,39E-09 | **1,48E-05** | 5.73 | 1,81E-05 | SBP |
| 29 | rs6532023* | 4q21.1 | *MEPE** | 5,44E-12 | **5,10E-07** | 6.73 | 2,10E-07 | LDL |
| 29 | rs1471399 | 4q21.1 | *MEPE** | 5,44E-12 | **5,10E-07** | 6.73 | 1,73E-07 | LDL |
| 29 | rs1471403 | 4q21.1 | *MEPE** | 5,44E-12 | **5,10E-07** | 6.73 | 2,10E-07 | LDL |
| 32 | rs11755164* | 6p21.1-p12.3 | *SUPT3H** | 1,80E-07 | **4,15E-04** | -5.09 | 4,15E-04 | DBP |
| 32 | rs12526711 | 6p21.1-p12.3 | *SUPT3H** | 6,39E-07 | **1,05E-03** | 4.86 | 4,76E-04 | WHR |
| 32 | rs3799986 | 6p21.1-p12.3 | *SUPT3H** | 1,47E-06 | **2,47E-03** | -4.7 | 6,39E-04 | WHR |
| 33 | rs9479055 | 6q25.1 | *CCDC170** | 5,31E-12 | **5,10E-07** | -6.73 | 1,04E-07 | LDL |
| 33 | rs11753987 | 6q25.1 | *CCDC170** | 1,02E-11 | **5,10E-07** | -6.64 | 1,04E-07 | LDL |
| 33 | rs10872673 | 6q25.1 | *CCDC170** | 2,82E-13 | **5,10E-07** | -7.13 | 1,04E-07 | LDL |
| 33 | rs1856859 | 6q25.1 | *CCDC170** | 7,96E-16 | **5,10E-07** | 7.86 | 9,71E-08 | LDL |
| 33 | rs9371537 | 6q25.1 | *CCDC170** | 1,80E-15 | **5,10E-07** | 7.76 | 9,54E-08 | LDL |
| 33 | rs7761420 | 6q25.1 | *CCDC170** | 1,00E-16 | **5,10E-07** | -8.1 | 1,04E-07 | LDL |
| 33 | rs9397425 | 6q25.1 | *CCDC170** | 1,91E-15 | **5,10E-07** | 7.76 | 9,47E-08 | LDL |
| 33 | rs11759804 | 6q25.1 | *CCDC170** | 1,80E-15 | **5,10E-07** | 7.76 | 9,62E-08 | LDL |
| 33 | rs7753676 | 6q25.1 | *CCDC170** | 1,22E-16 | **5,10E-07** | -8.08 | 9,71E-08 | LDL |
| 33 | rs9479072 | 6q25.1 | *CCDC170** | 1,78E-15 | **5,10E-07** | -7.76 | 1,04E-07 | LDL |
| 33 | rs1871859 | 6q25.1 | *CCDC170** | 1,64E-18 | **5,10E-07** | 8.57 | 9,47E-08 | LDL |
| 33 | rs9478223 | 6q25.1 | *CCDC170** | 4,26E-07 | **7,00E-04** | 4.94 | 5,45E-04 | T2D |
| 33 | rs6925996 | 6q25.1 | *CCDC170** | 7,38E-20 | **5,10E-07** | 8.9 | 9,54E-08 | LDL |
| 33 | rs9383930 | 6q25.1 | *CCDC170** | 3,39E-10 | **1,51E-06** | 6.13 | 1,55E-06 | SBP |
| 33 | rs9397065 | 6q25.1 | *CCDC170** | 4,72E-10 | **2,30E-06** | 6.08 | 1,85E-06 | SBP |
| 33 | rs1340874 | 6q25.1 | *CCDC170** | 7,39E-10 | **3,45E-06** | 6.01 | 3,18E-06 | HDL |
| 33 | rs11155800 | 6q25.1 | *CCDC170** | 1,10E-11 | **5,10E-07** | 6.63 | 3,00E-07 | TG |
| 33 | rs6904261 | 6q25.1 | *CCDC170** | 2,54E-15 | **5,10E-07** | 7.72 | 3,18E-07 | TG |
| 33 | rs6932603 | 6q25.1 | *CCDC170** | 1,59E-18 | **5,10E-07** | 8.57 | 3,73E-07 | T2D |
| 33 | rs10872676 | 6q25.1 | *CCDC170** | 1,04E-17 | **5,10E-07** | 8.36 | 1,32E-07 | HDL |
| 33 | rs7751941* | 6q25.1 | *CCDC170** | 5,27E-16 | **5,10E-07** | 7.91 | 3,30E-07 | SBP |
| 33 | rs7752591 | 6q25.1 | *CCDC170** | 1,75E-17 | **5,10E-07** | 8.3 | 3,38E-07 | T2D |
| 33 | rs6913578 | 6q25.1 | *CCDC170** | 1,38E-16 | **5,10E-07** | 8.07 | 3,37E-07 | DBP |
| 33 | rs865898 | 6q25.1 | *CCDC170** | 2,76E-13 | **5,10E-07** | 7.13 | 3,11E-07 | TG |
| 33 | rs712219 | 6q24-q27 | *ESR1* | 2,19E-12 | **5,10E-07** | 6.85 | 3,11E-07 | T1D |
| 33 | rs851970 | 6q24-q27 | *ESR1* | 4,63E-12 | **5,10E-07** | 6.75 | 3,37E-07 | T2D |
| 33 | rs6557164 | 6q24-q27 | *ESR1* | 3,97E-09 | **1,48E-05** | 5.74 | 1,26E-05 | SBP |
| 33 | rs3020333 | 6q24-q27 | *ESR1* | 1,59E-14 | **5,10E-07** | -7.5 | 1,40E-07 | HDL |
| 33 | rs3020334 | 6q24-q27 | *ESR1* | 5,51E-15 | **5,10E-07** | -7.63 | 1,32E-07 | HDL |
| 33 | rs851982 | 6q24-q27 | *ESR1* | 1,02E-11 | **5,10E-07** | -6.64 | 1,53E-07 | HDL |
| 33 | rs851980 | 6q24-q27 | *ESR1* | 1,41E-07 | **2,99E-04** | -5.14 | 3,44E-04 | HDL |
| 33 | rs2982554 | 6q24-q27 | *ESR1* | 7,84E-14 | **5,10E-07** | -7.29 | 1,58E-07 | HDL |
| 33 | rs3020349 | 6q24-q27 | *ESR1* | 6,60E-14 | **5,10E-07** | -7.32 | 1,34E-07 | HDL |
| 33 | rs2152750 | 6q24-q27 | *ESR1* | 1,25E-14 | **5,10E-07** | -7.53 | 1,58E-07 | HDL |
| 33 | rs1124674 | 6q24-q27 | *ESR1* | 1,03E-11 | **5,10E-07** | -6.64 | 1,64E-07 | HDL |
| 33 | rs2504070 | 6q24-q27 | *ESR1* | 1,55E-09 | **6,35E-06** | -5.89 | 6,09E-06 | SBP |
| 33 | rs1890010 | 6q24-q27 | *ESR1* | 1,99E-11 | **5,10E-07** | -6.55 | 2,83E-07 | HDL |
| 33 | rs2504069 | 6q24-q27 | *ESR1* | 1,99E-11 | **5,10E-07** | -6.55 | 2,50E-07 | HDL |
| 33 | rs2504063 | 6q24-q27 | *ESR1* | 3,36E-12 | **5,10E-07** | -6.79 | 1,98E-07 | HDL |
| 34 | rs10226308* | 7p15.2 | *NME8** | 1,45E-06 | **2,47E-03** | -4.7 | 1,69E-03 | LDL |
| 34 | rs10256195 | 7p15.2 | *NME8** | 1,45E-06 | **2,47E-03** | -4.7 | 1,29E-03 | LDL |
| 34 | rs10276139 | 7p15.2 | *NME8** | 1,45E-06 | **2,47E-03** | -4.7 | 1,69E-03 | LDL |
| 34 | rs17236800 | 7p14.1 | *SFRP4* | 1,45E-06 | **2,47E-03** | -4.7 | 1,29E-03 | LDL |
| 34 | rs10264106 | 7p14.1 | *SFRP4* | 1,67E-06 | **2,47E-03** | -4.67 | 1,13E-03 | LDL |
| 35 | rs2722281 | 7p14.1 | *EPDR1* | 1,44E-06 | **2,47E-03** | 4.7 | 8,75E-04 | SBP |
| 35 | rs2722286 | 7p14.1 | *EPDR1* | 1,50E-06 | **2,47E-03** | 4.7 | 9,55E-04 | SBP |
| 35 | rs1717739 | 7p14.1 | *EPDR1* | 8,19E-09 | **2,80E-05** | -5.63 | 4,58E-05 | LDL |
| 35 | rs1721396 | 7p14.1 | *EPDR1* | 1,57E-08 | **5,18E-05** | -5.52 | 6,94E-05 | DBP |
| 35 | rs1357651 | 7p14.1 | *EPDR1* | 1,81E-16 | **5,10E-07** | -8.04 | 3,58E-07 | T2D |
| 35 | rs1721385 | 7p14.1 | *EPDR1* | 9,67E-09 | **3,45E-05** | -5.6 | 4,62E-05 | LDL |
| 35 | rs1717731 | 7p14.1 | *EPDR1* | 9,67E-09 | **3,45E-05** | -5.6 | 4,62E-05 | LDL |
| 35 | rs1403987 | 7p14-p13 | *STARD3NL** | 2,19E-16 | **5,10E-07** | -8.01 | 3,76E-07 | T2D |
| 35 | rs6959212* | 7p14-p13 | *STARD3NL** | 1,18E-17 | **5,10E-07** | -8.35 | 3,00E-07 | TG |
| 35 | rs1524058 | 7p14-p13 | *STARD3NL** | 4,26E-17 | **5,10E-07** | -8.2 | 3,30E-07 | SBP |
| 37 | rs6952113 | 7q31.31 | *CPED1* | 1,56E-06 | **2,47E-03** | 4.69 | 1,57E-03 | SBP |
| 37 | rs13245690* | 7q31.31 | *CPED1* | 4,08E-07 | **7,00E-04** | 4.94 | 4,97E-04 | T2D |
| 39 | rs3779381 | 7q31 | *WNT16** | 8,74E-14 | **5,10E-07** | -7.28 | 2,74E-07 | LDL |
| 39 | rs718766 | 7q22.1-q31.1 | *FAM3C* | 5,29E-17 | **5,10E-07** | -8.18 | 2,51E-07 | LDL |
| 39 | rs7776725 | 7q22.1-q31.1 | *FAM3C* | 6,30E-17 | **5,10E-07** | -8.16 | 1,91E-07 | LDL |
| 40 | rs3134036 | 8q24 | *SAMD12-AS1* | 7,45E-08 | **2,09E-04** | -5.25 | 2,18E-04 | DBP |
| 40 | rs3133582 | 8q24 | *SAMD12-AS1* | 7,39E-07 | **1,30E-03** | -4.83 | 4,09E-04 | WHR |
| 40 | rs3133585 | 8q24 | *TNFRSF11B** | 2,46E-11 | **5,10E-07** | -6.52 | 2,36E-07 | T1D |
| 40 | rs3134086 | 8q24 | *TNFRSF11B** | 7,48E-07 | **1,30E-03** | -4.83 | 5,57E-04 | WHR |
| 40 | rs16891598 | 8q24 | *TNFRSF11B** | 1,22E-08 | **4,23E-05** | 5.56 | 4,83E-05 | SBP |
| 40 | rs6651219 | 8q24 | *TNFRSF11B** | 1,43E-08 | **5,18E-05** | 5.53 | 5,96E-05 | SBP |
| 40 | rs4876868 | 8q24 | *TNFRSF11B** | 6,26E-06 | **8,05E-03** | -4.41 | 9,01E-03 | T2D |
| 40 | rs11573885 | 8q24 | *TNFRSF11B** | 1,26E-12 | **5,10E-07** | -6.93 | 3,00E-07 | TG |
| 40 | rs1032129 | 8q24 | *TNFRSF11B** | 3,05E-06 | **4,49E-03** | 4.56 | 1,87E-03 | SBP |
| 40 | rs11573829 | 8q24 | *TNFRSF11B** | 5,24E-20 | **5,10E-07** | 8.94 | 3,35E-07 | T2D |
| 40 | rs10505346 | 8q24 | *TNFRSF11B** | 6,95E-09 | **2,27E-05** | 5.65 | 3,33E-05 | SBP |
| 40 | rs3102735 | 8q24 | *TNFRSF11B** | 7,09E-06 | **9,79E-03** | 4.38 | 7,38E-03 | SBP |
| 40 | rs1385499 | 8q24 | *TNFRSF11B** | 1,73E-16 | **5,10E-07** | 8.04 | 3,46E-07 | T2D |
| 40 | rs1564860 | 8q24 | *TNFRSF11B** | 1,90E-14 | **5,10E-07** | 7.47 | 2,98E-07 | LDL |
| 40 | rs7839059 | 8q24 | *TNFRSF11B** | 2,59E-17 | **5,10E-07** | 8.26 | 3,73E-07 | T2D |
| 40 | rs1825511 | 8q24 | *TNFRSF11B** | 1,09E-09 | **4,22E-06** | 5.95 | 7,73E-06 | TG |
| 40 | rs6469804 | 8q23-q24.1 | *COLEC10* | 7,67E-19 | **5,10E-07** | 8.65 | 2,36E-07 | T1D |
| 40 | rs10955924 | 8q23-q24.1 | *COLEC10* | 9,61E-20 | **5,10E-07** | 8.87 | 2,36E-07 | T1D |
| 45 | rs4568902 | 10p12.1 | *MPP7** | 1,26E-09 | **5,17E-06** | -5.93 | 1,22E-05 | TG |
| 45 | rs3905706* | 10p12.1 | *MPP7** | 1,19E-09 | **5,17E-06** | -5.94 | 8,03E-06 | WHR |
| 45 | rs7088552 | 10p12.1 | *MPP7** | 1,63E-09 | **6,35E-06** | -5.89 | 1,08E-05 | T1D |
| 46 | rs1373004* | 10q11.2 | *MBL2** | 2,52E-08 | **7,77E-05** | -5.44 | 1,13E-04 | SBP |
| 48 | rs7071206* | 10q22 | *KCNMA1** | 4,33E-13 | **5,10E-07** | -7.07 | 3,38E-07 | T2D |
| 48 | rs1877998 | 10q22 | *KCNMA1** | 1,16E-11 | **5,10E-07** | -6.62 | 3,06E-07 | TG |
| 48 | rs10509391 | 10q22 | *KCNMA1** | 1,60E-11 | **5,10E-07** | -6.58 | 3,00E-07 | TG |
| 50 | rs9787942 | 11p15.3 | *SOX6** | 1,03E-05 | 1,19E-02 | 4.31 | 8,08E-03 | T2D |
| 51 | rs11023718 | 11p15.3 | *SOX6** | 1,11E-05 | 1,19E-02 | -4.29 | 4,40E-03 | SBP |
| 52 | rs10219384 | 11p15.3 | *SOX6** | 7,42E-06 | **9,79E-03** | 4.37 | 3,57E-03 | DBP |
| 52 | rs297366 | 11p15.3 | *SOX6** | 7,85E-06 | **9,79E-03** | 4.36 | 3,57E-03 | DBP |
| 52 | rs297365 | 11p15.3 | *SOX6** | 7,85E-06 | **9,79E-03** | 4.36 | 3,57E-03 | DBP |
| 52 | rs2351964 | 11p15.3 | *SOX6** | 7,85E-06 | **9,79E-03** | 4.36 | 3,57E-03 | DBP |
| 54 | rs2021807 | 11p14.1 | *DCDC5** | 5,30E-08 | **1,42E-04** | -5.31 | 1,64E-04 | WHR |
| 54 | rs911268 | 11p14.1 | *DCDC5** | 5,30E-08 | **1,42E-04** | -5.31 | 1,60E-04 | WHR |
| 54 | rs507969 | 11p14.1 | *DCDC5** | 4,78E-08 | **1,42E-04** | 5.33 | 1,70E-04 | WHR |
| 54 | rs163883 | 11p14.1 | *DCDC5** | 5,42E-08 | **1,42E-04** | 5.31 | 1,92E-04 | WHR |
| 54 | rs163879* | 11p14.1 | *DCDC5** | 4,22E-08 | **1,16E-04** | 5.35 | 2,11E-04 | TG |
| 57 | rs3736228* | 11q13.4 | *LRP5** | 5,10E-12 | **5,10E-07** | 6.74 | 2,74E-07 | LDL |
| 57 | rs11228262 | 11q13 | *PPP6R3* | 3,83E-11 | **5,10E-07** | 6.45 | 3,82E-07 | SBP |
| 57 | rs11228292 | 11q13 | *PPP6R3* | 1,20E-10 | **6,36E-07** | 6.28 | 6,89E-07 | SBP |
| 57 | rs4930238 | 11q13.2 | *GAL* | 3,76E-07 | **7,00E-04** | -4.96 | 5,75E-04 | SBP |
| 57 | rs7935394 | 11q13.2 | *GAL* | 1,06E-06 | **1,61E-03** | -4.76 | 1,15E-03 | WHR |
| 57 | rs2510387 | 11q13.2 | *GAL* | 1,23E-05 | 1,44E-02 | -4.27 | 5,65E-03 | WHR |
| 59 | rs4283041 | 12p13 | *LINC00942* | 1,13E-07 | **2,99E-04** | -5.18 | 3,12E-04 | DBP |
| 59 | rs2887571* | 12p13 | *LINC00942* | 1,10E-07 | **2,51E-04** | -5.18 | 3,15E-04 | DBP |
| 62 | rs1054442 | 12q13 | *DDN* | 2,79E-07 | **4,89E-04** | -5.01 | 6,67E-04 | TG |
| 62 | rs11168850 | 12q13.12 | *RHEBL1* | 1,23E-06 | **2,00E-03** | -4.73 | 2,24E-03 | T2D |
| 62 | rs6580699 | 12q13.1 | *DHH** | 1,39E-06 | **2,00E-03** | -4.71 | 1,84E-03 | T1D |
| 63 | rs10747666 | 12q13 | *AAAS* | 2,76E-11 | **5,10E-07** | 6.5 | 2,83E-07 | HDL |
| 63 | rs2016266* | 12q13.13 | *SP7** | 4,81E-12 | **5,10E-07** | 6.74 | 2,22E-07 | HDL |
| 65 | rs10778517 | 12q23.3 | *TMEM263(C12orf23)** | 2,08E-06 | **3,02E-03** | -4.63 | 2,46E-03 | T2D |
| 65 | rs1053051* | 12q23.3 | *TMEM263(C12orf23)** | 1,39E-06 | **2,00E-03** | -4.71 | 3,47E-03 | LDL |
| 67 | rs9590679 | 13q14.11 | *DGKH* | 8,28E-06 | **9,79E-03** | 4.35 | 5,49E-03 | DBP |
| 67 | rs12861586 | 13q | *AKAP11** | 1,14E-06 | **2,00E-03** | -4.75 | 8,98E-04 | DBP |
| 67 | rs7995240 | 13q | *AKAP11** | 2,33E-12 | **5,10E-07** | -6.84 | 1,53E-07 | HDL |
| 67 | rs7998154 | 13q | *AKAP11** | 2,05E-07 | **4,15E-04** | 5.07 | 4,82E-04 | LDL |
| 67 | rs238270 | 13q | *AKAP11** | 5,23E-11 | **5,10E-07** | -6.41 | 2,23E-07 | HDL |
| 67 | rs7338012 | 13q | *AKAP11** | 9,17E-08 | **2,51E-04** | 5.21 | 3,64E-04 | DBP |
| 67 | rs7992970 | 13q | *AKAP11** | 1,02E-12 | **5,10E-07** | -6.96 | 3,98E-07 | T2D |
| 67 | rs7988075 | 13q | *AKAP11** | 8,83E-13 | **5,10E-07** | -6.98 | 3,98E-07 | T2D |
| 67 | rs1359201 | 13q | *AKAP11** | 1,31E-11 | **5,10E-07** | -6.6 | 2,30E-07 | LDL |
| 67 | rs1475249 | 13q | *AKAP11** | 1,59E-13 | **5,10E-07** | -7.2 | 3,76E-07 | T2D |
| 67 | rs11840862 | 13q | *AKAP11** | 1,35E-24 | **5,10E-07** | 9.99 | 4,32E-07 | LDL |
| 67 | rs17638544 | 13q | *AKAP11** | 1,09E-13 | **5,10E-07** | -7.25 | 3,37E-07 | DBP |
| 67 | rs7317323 | 13q | *AKAP11** | 1,40E-11 | **5,10E-07** | -6.6 | 3,37E-07 | DBP |
| 67 | rs9533093 | 13q | *AKAP11** | 5,27E-12 | **5,10E-07** | -6.73 | 2,30E-07 | LDL |
| 67 | rs17457561 | 13q | *AKAP11** | 9,09E-17 | **5,10E-07** | 8.12 | 2,36E-07 | T1D |
| 67 | rs7987211 | 13q | *AKAP11** | 1,29E-13 | **5,10E-07** | -7.23 | 2,51E-07 | LDL |
| 67 | rs7992415 | 13q | *AKAP11** | 1,29E-13 | **5,10E-07** | -7.23 | 3,29E-07 | TG |
| 67 | rs7994531 | 13q | *AKAP11** | 5,76E-12 | **5,10E-07** | -6.72 | 2,10E-07 | LDL |
| 67 | rs7326472 | 13q | *AKAP11** | 2,78E-15 | **5,10E-07** | -7.71 | 3,06E-07 | TG |
| 67 | rs9566973 | 13q | *AKAP11** | 1,15E-11 | **5,10E-07** | -6.62 | 2,10E-07 | LDL |
| 67 | rs9533099 | 13q | *AKAP11** | 2,58E-11 | **5,10E-07** | -6.51 | 1,91E-07 | LDL |
| 67 | rs9594759 | 13q14 | *TNFSF11* | 9,84E-16 | **5,10E-07** | 7.84 | 2,51E-07 | LDL |
| 67 | rs2062305 | 13q14 | *TNFSF11* | 1,22E-16 | **5,10E-07** | 8.08 | 3,21E-07 | HDL |
| 67 | rs6561055 | 13q14 | *TNFSF11* | 1,03E-12 | **5,10E-07** | -6.95 | 3,00E-07 | TG |
| 67 | rs9533128 | 13q14 | *TNFSF11* | 6,73E-10 | **2,82E-06** | 6.02 | 3,17E-06 | T1D |
| 70 | rs1286083* | 14q31-q32.1 | *RPS6KA5** | 5,32E-12 | **5,10E-07** | -6.73 | 4,25E-07 | T2D |
| 70 | rs1286079 | 14q31-q32.1 | *RPS6KA5** | 6,07E-12 | **5,10E-07** | -6.71 | 4,25E-07 | T2D |
| 70 | rs1286077 | 14q31-q32.1 | *RPS6KA5** | 6,47E-12 | **5,10E-07** | -6.7 | 4,55E-07 | T1D |
| 70 | rs1286153 | 14q31-q32.1 | *RPS6KA5** | 5,68E-12 | **5,10E-07** | -6.72 | 4,25E-07 | T2D |
| 70 | rs1286150 | 14q31-q32.1 | *RPS6KA5** | 5,68E-12 | **5,10E-07** | -6.72 | 5,76E-07 | TG |
| 70 | rs1286147 | 14q31-q32.1 | *RPS6KA5** | 5,32E-12 | **5,10E-07** | -6.73 | 4,25E-07 | T2D |
| 73 | rs9921222* | 16p13.3 | *AXIN1/LUC7L* | 9,85E-09 | **3,45E-05** | 5.6 | 4,66E-05 | WHR |
| 74 | rs4985155* | 16p13.11 | *PDXDC1* | 4,66E-07 | **8,53E-04** | -4.92 | 5,70E-04 | LDL |
| 74 | rs2740 | 16p13.11 | *PDXDC1/NTAN1* | 1,31E-06 | **2,00E-03** | -4.72 | 1,75E-03 | TG |
| 74 | rs16966952 | 16p13.11 | *PDXDC1/NTAN1* | 5,84E-07 | **1,05E-03** | -4.88 | 7,00E-04 | WHR |
| 75 | rs1564981* | 16q12-q13 | *CYLD** | 6,95E-09 | **2,27E-05** | 5.65 | 2,49E-05 | T1D |
| 75 | rs1872678 | 16q12-q13 | *CYLD** | 6,95E-09 | **2,27E-05** | 5.65 | 2,49E-05 | T1D |
| 76 | rs299946 | 16q24 | *FOXL1** | 4,25E-06 | **5,45E-03** | -4.49 | 3,68E-03 | WHR |
| 76 | rs10048146* | 16q24 | *FOXL1** | 1,34E-08 | **4,23E-05** | 5.54 | 5,78E-05 | HDL |
| 77 | rs1983490 | 17q21.31 | *MEOX1* | 2,33E-08 | **7,77E-05** | -5.45 | 1,02E-04 | SBP |
| 77 | rs1828720 | 17q12-q21 | *SOST** | 5,67E-09 | **2,27E-05** | -5.69 | 2,60E-05 | TG |
| 77 | rs4792909* | 17q12-q21 | *SOST** | 3,76E-09 | **1,48E-05** | -5.75 | 2,29E-05 | SBP |
| 78 | rs227584* | 17q21.31 | *C17orf53** | 2,48E-07 | **4,89E-04** | -5.04 | 2,04E-04 | SBP |
| 78 | rs7207464 | 17q21.31 | *ASB16* | 1,75E-07 | **3,53E-04** | -5.1 | 1,81E-04 | SBP |
| 84 | rs2980980 | 18q21.33 | *KIAA1468* | 6,49E-07 | **1,05E-03** | -4.86 | 1,54E-03 | TG |
| 84 | rs17720953 | 18q22.1 | *TNFRSF11A** | 2,56E-05 | 2,55E-02 | 4.11 | 8,38E-03 | WHR |
| 84 | rs8083511 | 18q22.1 | *TNFRSF11A** | 3,83E-07 | **7,00E-04** | -4.96 | 6,35E-04 | T2D |
| 85 | rs8089829 | 18q22.1 | *TNFRSF11A** | 4,23E-06 | **5,45E-03** | -4.49 | 3,21E-03 | WHR |
| 86 | rs884205* | 18q22.1 | *TNFRSF11A** | 2,02E-09 | **7,83E-06** | -5.85 | 1,50E-05 | T2D |
| 86 | rs2957128 | 18q22.1 | *TNFRSF11A** | 1,13E-08 | **4,23E-05** | -5.57 | 6,44E-05 | TG |
| 88 | rs13343954 | 19q13.12 | *RHPN2* | 7,64E-06 | **9,79E-03** | -4.37 | 7,88E-03 | TG |
| 88 | rs9304844 | 19q13.12 | *GPATCH1** | 4,48E-09 | **1,84E-05** | -5.72 | 1,50E-05 | LDL |
| 88 | rs7247748 | 19q13.12 | *GPATCH1** | 4,48E-09 | **1,84E-05** | -5.72 | 1,50E-05 | LDL |
| 88 | rs3760893 | 19q13.12 | *GPATCH1** | 4,48E-09 | **1,84E-05** | -5.72 | 1,50E-05 | LDL |
| 88 | rs10416218* | 19q13.12 | *GPATCH1** | 3,94E-09 | **1,48E-05** | -5.75 | 1,50E-05 | LDL |
| 88 | rs2287679 | 19q13.12 | *GPATCH1** | 4,48E-09 | **1,84E-05** | -5.72 | 1,50E-05 | LDL |
| 91 | rs10485741 | 20p12.1-p11.23 | *JAG1** | 6,40E-06 | **8,05E-03** | 4.4 | 4,00E-03 | T1D |
| 91 | rs3790159 | 20p12.1-p11.23 | *JAG1** | 1,79E-11 | **5,10E-07** | 6.56 | 4,91E-07 | T2D |
| 91 | rs3790160* | 20p12.1-p11.23 | *JAG1** | 1,79E-11 | **5,10E-07** | 6.56 | 4,91E-07 | T2D |
| 91 | rs17457340 | 20p12.1-p11.23 | *JAG1** | 1,43E-06 | **2,47E-03** | 4.7 | 1,62E-03 | DBP |
| 91 | rs6040061 | 20p12.1-p11.23 | *JAG1** | 1,80E-11 | **5,10E-07** | 6.56 | 3,31E-07 | T1D |
| 92 | rs1108850 | 20p12.1-p11.23 | *JAG1** | 3,10E-06 | **4,49E-03** | 4.55 | 1,95E-03 | WHR |
| 92 | rs6040286 | 20p12.1-p11.23 | *JAG1** | 6,39E-06 | **8,05E-03** | -4.4 | 2,95E-03 | DBP |
| 92 | rs6104690 | 20p12.1-p11.23 | *JAG1** | 5,70E-06 | **8,05E-03** | -4.43 | 2,95E-03 | DBP |
| 93 | rs6040357 | 20p12.1-p11.23 | *JAG1** | 3,24E-06 | **4,49E-03** | 4.54 | 3,61E-03 | TG |
| Independent complex or single gene loci (LD-r2 < 0.2) with SNP(s) with a conditional FDR (condFDR) < 0.01 in bone mineral density (BMD, femoral neck) given the association in other phenotypes. We defined the most significant BMD associated SNPs in each LD block based on the minimum condFDR (min condFDR) for each phenotype. The second phenotype which provides the minimal FDR signal (Driving phenotype) is listed. All loci with SNPs with condFDR < 0.01 were used to define the number of the loci. The following abbreviations were used: type 1 diabetes (T1D), type 2 diabetes (T2D), systolic blood pressure (SBP), diastolic blood pressure (DBP), high density lipoprotein (HDL), low density lipoprotein (LDL), triglycerides (TG), waist hip ratio (WHR), chromosome location (Map Loc.). Shaded r values represent nominally significant (p<0.05) Pearson correlations (age and BMI adjusted LS BMD vs Affymetrix signal values). NA: not applicable (undetected). SNPs and Genes previously reported to associate with BMD are marked with stars (*). Wald stats: z-score transformed from p values. | | | | | | | | |

### S5 Table. Gene titles and gene ontology function terms of genes associated with LS an FN BMD loci at FDR <0.01

| **Gene Symbol** | **Gene Title** | **GO molecular function term** |
| --- | --- | --- |
| *AAAS* | achalasia, adrenocortical insufficiency, alacrimia | --- |
| *AAGAB* | alpha- and gamma-adaptin binding protein |  |
| *ABCF2* | ATP-binding cassette, sub-family F (GCN20), member 2 | nucleotide binding; transporter activity;  ATP binding; ATPase activity |
| *ADCY6* | adenylate cyclase 6 | receptor binding; protein binding; ATP binding; calcium- and calmodulin-responsive adenylate cyclase activity;phosphorus-oxygen lyase activity; protein kinase binding; metal ion binding |
| *AKAP11* | A kinase (PRKA) anchor protein 11 | protein phosphatase 1 binding ; protein complex scaffold; protein kinase A catalytic subunit binding;  protein kinase A regulatory subunit binding |
| *AMBRA1* | autophagy/beclin-1 regulator 1 | --- |
| *ANAPC1* | anaphase promoting complex subunit 1 | --- |
| *APEH* | acylaminoacyl-peptide hydrolase |  |
| *AREG* | Amphiregulin | cytokine activity; growth factor activity |
| *ARHGAP1* | Rho GTPase activating protein 1 | SH3/SH2 adaptor activity; Rho GTPase activator activity;  protein binding; GTP binding; SH3 domain binding; Rac GTPase activator activity |
| *ASB16* | ankyrin repeat and SOCS box containing 16 | protein binding |
| *ASB16-AS1* | ASB16 antisense RNA 1 | --- |
| *ATF7* | activating transcription factor 7 | sequence-specific DNA binding transcription factor activity; transcription factor binding; zinc ion binding; protein dimerization activity; mitogen-activated protein kinase binding |
| *ATP11B* | ATPase, class VI, type 11B |  |
| *AXIN1* | axin 1 | p53 binding; signal transducer activity; GTPase activator activity; beta-catenin binding; protein C-terminus binding; protein kinase binding; protein domain specific binding; ubiquitin protein ligase binding; protein complex scaffold; protein homodimerization activity; SMAD binding; armadillo repeat domain binding; I-SMAD binding; R-SMAD binding |
| *BDNF-AS* | brain-derived neurotrophic factor antisense RNA |  |
| *BET1L* | blocked early in transport 1 homolog (S. cerevisiae)-like | SNAP receptor activity |
| *BMP7* | bone morphogenetic protein 7 | cytokine activity; protein binding; growth factor activity |
| *BSN* | bassoon (presynaptic cytomatrix protein) | metal ion binding |
| *BSN-AS2* | BSN antisense RNA 2 |  |
| *C12orf23* | chromosome 12 open reading frame 23 | --- |
| *C17orf53* | chromosome 17 open reading frame 53 | --- |
| *C2orf73* | chromosome 2 open reading frame 73 | --- |
| *C7orf76* | chromosome 7 open reading frame 76 | --- |
| *CAPZA1* | Capping protein (actin filament) muscle Z-line, alpha 1 | actin binding |
| *CBR3-AS1* |  |  |
| *CCDC170* | coiled-coil domain containing 170 | --- |
| *CDK15* | cyclin-dependent kinase 15 | nucleotide binding; protein kinase activity; protein serine/threonine kinase activity; cyclin-dependent protein kinase activity; ATP binding ; kinase activity; transferase activity; transferase activity, transferring phosphorus-containing groups; metal ion binding |
| *CDKAL1* | CDK5 regulatory subunit associated protein 1-like 1 | catalytic activity; transferase activity; metal ion binding; 4 iron, 4 sulfur cluster binding |
| *CENPW* | centromere protein W |  |
| *CEP112* | centrosomal protein 112kDa |  |
| *COL11A1* | collagen, type XI, alpha 1 | extracellular matrix structural constituent; protein binding, bridging |
| *COLEC10* | collectin sub-family member 10 (C-type lectin) | mannose binding ; carbohydrate binding |
| *CPED1* | cadherin-like and PC-esterase domain containing 1 | --- |
| *CTNNB1* | catenin (cadherin-associated protein), beta 1, 88kDa | chromatin binding; sequence-specific DNA binding transcription factor activity; transcription coactivator activity; signal transducer activity; structural molecule activity; protein binding; protein C-terminus binding ;transcription factor binding; enzyme binding; kinase binding ; protein kinase binding; protein phosphatase binding ;estrogen receptor binding; ionotropic glutamate receptor binding; RPTP-like protein binding ; ion channel binding; alpha-catenin binding; cadherin binding ; SMAD binding; androgen receptor binding ;  I-SMAD binding; R-SMAD binding |
| *CYLD* | cylindromatosis (turban tumor syndrome) | ubiquitin thiolesterase activity; cysteine-type peptidase activity; zinc ion binding; protein kinase binding; proline-rich region binding |
| *CYP19A1* | cytochrome P450, family 19, subfamily A, polypeptide 1 | monooxygenase activity; iron ion binding; electron carrier activity; oxidoreductase activity, acting on paired donors, with incorporation or reduction of molecular oxygen, reduced flavin or flavoprotein as one donor, and incorporation of one atom of oxygen; oxidoreductase activity, acting on paired donors, with incorporation or reduction of molecular oxygen, reduced flavin or flavoprotein as one donor, and incorporation of one atom of oxygen; oxygen binding; heme binding ; aromatase activity |
| *DCDC5* | doublecortin domain containing 5 | --- |
| *DDN* | dendrin | protein binding |
| *DDR2* | Discoidin domain receptor tyrosine kinase 2 | transmembrane receptor protein tyrosine kinase activity; collagen binding; ATP binding |
| *DGKH* | diacylglycerol kinase, eta |  |
| *DHH* | desert hedgehog | patched binding; calcium ion binding; protein binding; peptidase activity; zinc ion binding; hydrolase activity |
| *DNM3* | dynamin 3 | GTPase activity; protein binding; GTP binding; phospholipid binding; hydrolase activity |
| *DSPP* | dentin sialophosphoprotei | extracellular matrix structural constituent; calcium ion binding; collagen binding |
| *EPDR1* | ependymin related protein 1 (zebrafish) | calcium ion binding |
| *ERCC1* | excision repair cross-complementing rodent repair deficiency, complementation group 1 (includes overlapping antisense sequence) | single-stranded DNA specific endodeoxyribonuclease activity; DNA binding; damaged DNA binding; protein domain specific binding; structure-specific DNA binding |
| *ESR1* | estrogen receptor 1 | chromatin binding; sequence-specific DNA binding transcription factor activity; steroid hormone receptor activity; ligand-activated sequence-specific DNA binding RNA polymerase II transcription factor activity; steroid binding; beta-catenin binding; zinc ion binding; lipid binding; enzyme binding; nitric-oxide synthase regulator activity; estrogen receptor activity; type 1 metabotropic glutamate receptor binding; protein complex binding; estrogen response element binding; estrogen-activated sequence-specific DNA binding RNA polymerase II transcription factor activity; hormone binding; identical protein binding |
| *EYA1* | eyes absent homolog 1 (Drosophila) | phosphoprotein phosphatase activity; protein tyrosine phosphatase activity; protein binding; metal ion binding |
| *FAM20C* | Family with sequence similarity 20, member C |  |
| *FAM210A* | family with sequence similarity 210, member A | --- |
| *FAM3C* | family with sequence similarity 3, member C | cytokine activity |
| *FUBP3* | far upstream element (FUSE) binding protein 3 | DNA binding; RNA binding |
| *GAL* | galanin prepropeptide | neuropeptide hormone activity |
| *GALNT3* | UDP-N-acetyl-alpha-D-galactosamine:polypeptide N-acetylgalactosaminyltransferase 3 (GalNAc-T3) | polypeptide N-acetylgalactosaminyltransferase activity; calcium ion binding; transferase activity, transferring glycosyl groups; manganese ion binding; carbohydrate binding |
| *GNAZ* | guanine nucleotide binding protein (G protein), alpha z polypeptide |  |
| *GNG12-AS1* | GNG12 antisense RNA 1 |  |
| *GPATCH1* | G patch domain containing 1 | nucleic acid binding |
| *GRB10* | growth factor receptor-bound protein 10 | SH3/SH2 adaptor activity; insulin receptor binding; phospholipid binding |
| *HARBI1* | harbinger transposase derived 1 |  |
| *HOXC4* | homeobox C4 | sequence-specific DNA binding transcription factor activity; transcription corepressor activity; HMG box domain binding |
| *HOXC5* | homeobox C5 |
| *HOXC6* | homeobox C6 |
| *IBSP* | integrin-binding sialoprotein | --- |
| *IDUA* | Iduronidase, alpha-L- | L-iduronidase activity; hydrolase activity, hydrolyzing O-glycosyl compounds; cation binding |
| *IFT172* | intraflagellar transport 172 homolog (Chlamydomonas) | --- |
| *INSIG2* | insulin induced gene 2 | transcription factor binding |
| *JAG1* | Jagged 1 | Notch binding ; structural molecule activity; calcium ion binding ; growth factor activity |
| *KANSL1* | KAT8 regulatory NSL complex subunit 1 | protein binding; histone acetyltransferase activity (H4-K5 specific); histone acetyltransferase activity (H4-K8 specific); histone acetyltransferase activity (H4-K16 specific) |
| *KANSL1-AS1* | KANSL1 antisense RNA 1 |  |
| *KAT5* | K(lysine) acetyltransferase 5 | transcription coactivator activity; histone acetyltransferase activity; protein binding;metal ion binding; androgen receptor binding; repressing transcription factor binding |
| *KCNMA1* | potassium large conductance calcium-activated channel, subfamily M, alpha member 1 | nucleotide binding; actin binding; voltage-gated potassium channel activity; large conductance calcium-activated potassium channel activity |
| *KIAA1468* | KIAA1468 | --- |
| *KIAA2018* | KIAA2018 | DNA binding; mannosyl-oligosaccharide 1,2-alpha-mannosidase activity; calcium ion binding |
| *KIF2B* | kinesin family member 2B | microtubule motor activity; ATP binding |
| *KLF4* | Kruppel-like factor 4 (gut) | RNA polymerase II core promoter proximal region sequence-specific DNA binding transcription factor activity involved in positive regulation of transcription; zinc ion binding; phosphatidylinositol 3-kinase regulator activity; sequence-specific DNA binding |
| *KLF6* | Kruppel-like factor 6 |
| *KLHL42* | kelch-like family member 42 | --- |
| *LACTB2* | lactamase, beta 2 | hydrolase activity; metal ion binding |
| *LEKR1* | leucine, glutamate and lysine rich 1 | --- |
| *LGR4* | Leucine-rich repeat containing G protein-coupled receptor 4 | signal transducer activity; G-protein coupled receptor activity; protein-hormone receptor activity |
| *LINC00942* | long intergenic non-protein coding RNA 942 |  |
| *LRP4* | low density lipoprotein receptor-related protein 4 |  |
| *LRP4-AS1* | LRP4 antisense RNA 1 |  |
| *LRP5* | Low density lipoprotein receptor-related protein 5 | receptor activity; protein binding; coreceptor activity; Wnt-protein binding; toxin transporter activity; Wnt-activated receptor activity |
| *LUC7L* | LUC7-like (S. cerevisiae) |  |
| *MACF1* | microtubule-actin crosslinking factor 1 | actin binding; calcium ion binding;microtubule binding;ATPase activity |
| *MALAT1* | metastasis associated lung adenocarcinoma transcript 1 (non-protein coding) |  |
| *MARK3* | MAP/microtubule affinity-regulating kinase 3 | nucleotide binding; protein serine/threonine kinase activity; protein binding; ATP binding; kinase activity; transferase activity, transferring phosphorus-containing groups |
| *MBL2* | mannose-binding lectin (protein C) 2, soluble | receptor binding; protein binding; mannose binding; eukaryotic cell surface binding; calcium-dependent protein binding; bacterial cell surface binding |
| *MEF2C* | myocyte enhancer factor 2C | sequence-specific DNA binding RNA polymerase II transcription factor activity; histone deacetylase; protein heterodimerization activity, HMG box domain binding |
| *MEF2D* | myocyte enhancer factor 2D | sequence-specific DNA binding RNA polymerase II transcription factor activity; enzyme binding; activating transcription factor binding; protein homodimerization activity; histone deacetylase binding; protein heterodimerization activity |
| *MEPE* | matrix extracellular phosphoglycoprotein | extracellular matrix structural constituent ; protein binding |
| *METTL21A* | methyltransferase like 21A | methyltransferase activity |
| *MIR1208* | microRNA 1208 |  |
| *MIR4713* | microRNA 4713 |  |
| *MIR3120* | microRNA 3120 |  |
| *MIR1262* | microRNA 1262 |  |
| *MIR595* | microRNA 595 |  |
| *MIR4701* | microRNA 4701 |  |
| *MIR196A2* | microRNA 196A2 | --- |
| *MLXIP* | MLX interacting protein | DNA binding |
| *MPP7* | membrane protein, palmitoylated 7 (MAGUK p55 subfamily member 7) | protein domain specific binding; protein complex scaffold; signaling adaptor activity; protein heterodimerization activity |
| *NAB1* | NGFI-A binding protein 1 (EGR1 binding protein 1) | transcription factor binding |
| *NFATC1* | nuclear factor of activated T-cells, cytoplasmic, calcineurin-dependent 1 | RNA polymerase II transcription factor binding; RNA polymerase II distal enhancer sequence-specific DNA binding transcription factor activity involved in positive regulation of transcription; FK506 binding; mitogen-activated protein kinase p38 binding |
| *NME8* | NME/NM23 nucleoside diphosphate kinase 8 | nucleoside diphosphate kinase activity; ATP binding; kinase activity; transferase activity; metal ion binding |
| *NSF* | vesicle-fusing ATPase-like; N-ethylmaleimide-sensitive factor | protein binding; ATP binding; protein C-terminus binding; ATPase activity; nucleoside-triphosphatase activity; Rab GTPase binding; syntaxin binding; PDZ domain binding; protein complex binding; metal ion binding |
| *NTAN1* | N-terminal asparagine amidase |  |
| *PDXDC1* | pyridoxal-dependent decarboxylase domain containing 1 | carboxy-lyase activity; pyridoxal phosphate binding |
| *PKDCC* | protein kinase domain containing, cytoplasmic |  |
| *PIGN* | phosphatidylinositol glycan anchor biosynthesis, class N | catalytic activity; transferase activity |
| *PKIA* | protein kinase (cAMP-dependent, catalytic) inhibitor alpha | cAMP-dependent protein kinase inhibitor activity |
| *PKDCC* | protein kinase domain containing, cytoplasmic | --- |
| *PPP1CB* | Protein phosphatase 1, catalytic subunit, beta isozyme | protein serine/threonine phosphatase activity; protein binding; protein kinase binding; metal ion binding ; myosin-light-chain-phosphatase activity |
| *PPP6R3* | protein phosphatase 6, regulatory subunit 3 | protein phosphatase binding |
| *PTPRD* | protein tyrosine phosphatase, receptor type, D | protein tyrosine phosphatase activity; transmembrane receptor protein tyrosine phosphatase activity; receptor binding; cell adhesion molecule binding |
| *PTPRN2* | protein tyrosine phosphatase, receptor type, N polypeptide 2 | phosphoprotein phosphatase activity; receptor activity;  transmembrane receptor protein tyrosine phosphatase activity |
| *PTX4* | pentraxin 4, long | [metal ion binding](http://amigo.geneontology.org/cgi-bin/amigo/term_details?term=GO:0046872&session_id=8873amigo1366622851&) |
| *RAB36* | RAB36, member RAS oncogene family |  |
| *RAB9BP1* | RAB9B, member RAS oncogene family pseudogene 1 | --- |
| *RELA* | V-rel reticuloendotheliosis viral oncogene homolog A (avian) | chromatin binding; RNA polymerase II distal enhancer sequence-specific DNA binding transcription factor activity;ankyrin repeat binding |
| *RERE* | arginine-glutamic acid dipeptide (RE) repeats | sequence-specific DNA binding transcription factor activity; protein binding; poly-glutamine tract binding; zinc ion binding |
| *RHEBL1* | Ras homolog enriched in brain like 1 |  |
| *RHPN2* | rhophilin, Rho GTPase binding protein 2 | --- |
| *RIC8B* | resistance to inhibitors of cholinesterase 8 homolog B (C. elegans) | G-protein alpha-subunit binding; guanyl-nucleotide exchange factor activity |
| *RPS6KA5* | ribosomal protein S6 kinase, 90kDa, polypeptide 5 | nucleotide binding; magnesium ion binding; protein serine/threonine kinase activity; protein binding; ATP binding; transferase activity, transferring phosphorus-containing groups |
| *RTDR1* | rhabdoid tumor deletion region gene 1 | --- |
| *SALL1* | sal-like 1 (Drosophila) | chromatin binding; sequence-specific DNA binding transcription factor activity; histone deacetylase activity; protein binding; beta-catenin binding; zinc ion binding |
| *SAMD12-AS* | sterile alpha motif domain containing 12 antisense RNA 1 |  |
| *SEMA3D* | sema domain, immunoglobulin domain (Ig), short basic domain, secreted, (semaphorin) 3D | receptor activity |
| *SFRP4* | secreted frizzled-related protein 4 | Wnt-protein binding; PDZ domain binding; Wnt-activated receptor activity |
| *SHFM1* | split hand/foot malformation (ectrodactyly) type 1 | protein binding; peptidase activity |
| *SLC1A3* | solute carrier family 1 (glial high affinity glutamate transporter), member 3 | high-affinity glutamate transmembrane transporter activity; glutamate binding;sodium:dicarboxylate symporter activity |
| *SLC25A16* | Solute carrier family 25 (mitochondrial carrier; Graves disease autoantigen), member 16 | solute:solute antiporter activity |
| *SLX4IP* | SLX4 interacting protein |  |
| *SMAD3* | SMAD family member 3 | core promoter proximal region sequence-specific DNA binding; protein binding transcription factor activity; RNA polymerase II activating transcription factor binding; chromatin binding; transforming growth factor beta receptor binding; protein binding; collagen binding; beta-catenin binding; zinc ion binding; protein kinase binding; phosphatase binding; transforming growth factor beta receptor, pathway-specific cytoplasmic mediator activity; chromatin DNA binding; ubiquitin protein ligase binding; protein homodimerization activity; ubiquitin binding; sequence-specific DNA binding; sequence-specific DNA binding; transcription regulatory region DNA binding; SMAD binding; metal ion binding; co-SMAD binding; R-SMAD binding |
| *SMARCD3* | SWI/SNF related, matrix associated, actin dependent regulator of chromatin, subfamily d, member 3 | receptor binding; transcription factor binding; ligand-dependent nuclear receptor transcription coactivator activity |
| *SMG6* | smg-6 homolog, nonsense mediated mRNA decay factor (C. elegans) | DNA binding; endoribonuclease activity; protein binding; telomeric DNA binding ; metal ion binding |
| *SNORD67* | small nucleolar RNA, C/D box 67 |  |
| *SNORD27* | small nucleolar RNA, C/D box 27 |  |
| *SOST* | Sclerostin | transcription factor binding; heparin binding |
| *SOX6* | SRY (sex determining region Y)-box 6 | sequence-specific DNA binding transcription factor activity; protein heterodimerization activity |
| *SP7* | Sp7 transcription factor | DNA binding; zinc ion binding; DEAD/H-box RNA helicase binding; DEAD/H-box RNA helicase binding |
| *SPP1* | secreted phosphoprotein 1 |  |
| *SPTBN1* | spectrin, beta, non-erythrocytic 1 | actin binding; structural constituent of cytoskeleton; protein binding; calmodulin binding; phospholipid binding; ankyrin binding |
| *STARD3NL* | STARD3 N-terminal like | --- |
| *SUPT3H* | suppressor of Ty 3 homolog (S. cerevisiae) | DNA binding; transcription coactivator activity; histone acetyltransferase activity |
| *SUSD4* | sushi domain containing 4 | --- |
| *SUSD5* | sushi domain containing 5 | hyaluronic acid binding |
| *TESK2* | testis-specific kinase 2 | nucleotide binding; protein serine/threonine/tyrosine kinase activity; protein tyrosine kinase activity; ATP binding; transferase activity, transferring phosphorus-containing groups; metal ion binding |
| *TMEM135* | transmembrane protein 135 | --- |
| *TMEM175* | transmembrane protein 175 | --- |
| *TMEM194B* | transmembrane protein 194B | --- |
| *TNFRSF11A* | tumor necrosis factor receptor superfamily, member 11a, NFKB activator | transmembrane signaling receptor activity; tumor necrosis factor-activated receptor activity; protein binding; cytokine binding; metal ion binding |
| *TNFRSF11B* | tumor necrosis factor receptor superfamily, member 11b | receptor activity; cytokine activity |
| *TNFSF11* | tumor necrosis factor (ligand) superfamily, member 11 | receptor activity; cytokine activity ; tumor necrosis factor receptor superfamily binding |
| *ULK4* | unc-51-like kinase 4 (C. elegans) | protein serine/threonine kinase activity; ATP binding; transferase activity, transferring phosphorus-containing groups |
| *WLS* | wntless homolog | [Wnt-protein binding](http://amigo.geneontology.org/cgi-bin/amigo/term_details?term=GO:0017147&session_id=6401amigo1366622685&) |
| *WNT16* | wingless-type MMTV integration site family, member 16 | receptor binding; frizzled binding |
| *WNT2B* | wingless-type MMTV integration site family, member 2B | receptor binding; frizzled-2 binding |
| *WNT4* | wingless-type MMTV integration site family, member 4 | G-protein coupled receptor binding; transcription corepressor activity; frizzled binding; extracellular matrix structural constituent; receptor agonist activity |
| *XKR9* | XK, Kell blood group complex subunit-related family, member 9 | --- |
| *ZBTB40* | zinc finger and BTB domain containing 40 | DNA binding; zinc ion binding |
| *ZFP42* | zinc finger protein 42 homolog (mouse) | sequence-specific DNA binding transcription factor activity; zinc ion binding |
| *ZNF652* | Zinc finger protein 652 | DNA binding; protein binding; zinc ion binding |

### 
