# Supplementary material for: Genetic Sharing with Cardiovascular Disease Risk Factors and Diabetes Reveals Novel Bone Mineral Density Loci
Source: PLoS One. 2015 Dec 22;10(12):e0144531. doi: 10.1371/journal.pone.0144531 (PMC4687843; doi:10.1371/journal.pone.0144531)
Supplement: S1 Table — (DOCX) [file pone.0144531.s009.docx]

| **S1 Table. Summary data from all GWAS used in the current study** | | | | | |
| --- | --- | --- | --- | --- | --- |
| **Disease/Trait** | | **N** | **# SNPs** | **Overlap (%)** | **Reference** |
| Bone Mineral Density (BMD) |  | 32,961 | 2,500,000 | - | Estrada, K. et al. Genome-wide meta-analysis identifies 56 bone mineral density loci and reveals 14 loci associated with risk of fracture. Nat Genet 2012;44:491-501. |
| Type 2 Diabetes (T2D) |  | 22,044 | 2,426,886 | 19.4 | Lango Allen, H. et al. Hundreds of variants clustered in genomic loci and biological pathways affect human height. Nature 467, 832-8 (2010). |
| Type 1 Diabetes (T1D) |  | 16,559 | 841,622 | 0 | Barrett, J.C. et al .Genome-wide association study and meta-analysis find that over 40 loci affect risk of type 1 diabetes. Nat Genet 2009, 41, 703-7. |
| Systolic Blood Pressure (SBP) |  | 203,056 | 2,382,073 | 7.9 | Ehret GB, Munroe PB, Rice KM, et al. Genetic variants in novel pathways influence blood pressure and cardiovascular disease risk. Nature 2011;478:103-9. |
| Diastolic Blood Pressure (DBP) |  | 203,056 | 2,382,073 | 7.9 |  |
| High Density Lipoprotein (HDL) |  | 96,598 | 2,508,370 | 10.8 | Teslovich TM, Musunuru K, Smith AV, et al. Biological, clinical and population relevance of 95 loci for blood lipids. Nature 2010;466:707-13. |
| Low Density Lipoprotein (LDL) |  | 99,900 | 2,508,375 | 10.5 |  |
| Triglycerides (TG) |  | 96,568 | 2,508,369 | 10.8 |  |
| Waist to hip ratio  (WHR) |  | 77,167 | 2,376,820 | 21.0 | Heid IM, Jackson AU, Randall JC, et al. Meta-analysis identifies 13 new loci associated with waist-hip ratio and reveals sexual dimorphism in the genetic basis of fat distribution. Nat Genet 2010;42:949-60. |
| For more details, see also <http://www.genome.gov/gwastudies> | | | | | |
