# Supplementary material for: Genetic Sharing with Cardiovascular Disease Risk Factors and Diabetes Reveals Novel Bone Mineral Density Loci
Source: PLoS One. 2015 Dec 22;10(12):e0144531. doi: 10.1371/journal.pone.0144531 (PMC4687843; doi:10.1371/journal.pone.0144531)
Supplement: S2 Table — (DOCX) [file pone.0144531.s010.docx]

| **S2 Table. All identified loci associated with femoral neck BMD** | | | | | | | | |
| --- | --- | --- | --- | --- | --- | --- | --- | --- |
| **locus#** | **SNP** | **Map loc.** | **Gene Symbol** | **BMD**  **p-value** | **BMD FDR** | **Wald**  **stats** | **Min cond FDR** | **Driving phenotype** |
| 1 | rs10779702 | 1p36.23 | *RERE* | 7,78E-08 | **3,06E-04** | -5.26 | 1,60E-04 | HDL |
| 1 | rs894875 | 1p36.23 | *RERE* | 2,04E-08 | **8,99E-05** | -5.49 | 8,36E-05 | SBP |
| 1 | rs6678140 | 1p36.23 | *RERE* | 2,04E-08 | **8,99E-05** | -5.49 | 7,99E-05 | SBP |
| 2 | rs7521902* | 1p36.23-p35.1 | *WNT4** | 5,77E-08 | **2,52E-04** | 5.31 | 3,51E-04 | DBP |
| 3 | rs6690148 | 1p36 | *ZBTB40** | 5,14E-07 | **1,42E-03** | 4.92 | 1,32E-03 | HDL |
| 3 | rs10917209 | 1p36 | *ZBTB40** | 3,40E-07 | **9,66E-04** | 4.99 | 1,12E-03 | HDL |
| 3 | rs10917214 | 1p36 | *ZBTB40** | 3,60E-07 | **1,17E-03** | -4.98 | 1,41E-03 | LDL |
| 3 | rs12742784 | 1p36 | *ZBTB40** | 3,43E-17 | **6,36E-07** | -8.26 | 5,32E-07 | T2D |
| 3 | rs12029258 | 1p36 | *ZBTB40** | 6,96E-06 | 1,00E-02 | 4.4 | 6,39E-03 | SBP |
| 3 | rs12048810 | 1p36 | *ZBTB40** | 6,68E-06 | 1,00E-02 | 4.41 | 6,87E-03 | SBP |
| 4 | rs11810751 | 1p36 | *ZBTB40** | 2,20E-20 | **6,36E-07** | -9.06 | 2,67E-07 | HDL |
| 4 | rs12723796 | 1p36 | *ZBTB40** | 3,13E-16 | **6,36E-07** | -8 | 4,81E-07 | T2D |
| 4 | rs12568930 | 1p36 | *ZBTB40** | 5,89E-24 | **6,36E-07** | -9.89 | 5,32E-07 | T2D |
| 4 | rs11576345 | 1p36 | *ZBTB40** | 3,29E-07 | **9,66E-04** | -5 | 9,49E-04 | LDL |
| 4 | rs1320601 | 1p36 | *ZBTB40** | 3,39E-07 | **9,66E-04** | -5 | 8,30E-04 | LDL |
| 4 | rs7543680 | 1p36 | *ZBTB40** | 4,66E-16 | **6,36E-07** | -7.95 | 1,65E-07 | HDL |
| 4 | rs4394609 | 1p36 | *ZBTB40** | 1,38E-15 | **6,36E-07** | -7.82 | 5,06E-07 | T2D |
| 4 | rs4409621 | 1p36 | *ZBTB40** | 1,48E-15 | **6,36E-07** | -7.81 | 2,05E-07 | HDL |
| 5 | rs4655059 | 1p36 | *ZBTB40** | 2,55E-08 | **1,11E-04** | 5.46 | 8,41E-05 | SBP |
| 6 | rs12137389 | 1p32 | *TESK2* | 1,88E-06 | **4,15E-03** | 4.67 | 4,01E-03 | HDL |
| 7 | rs1430742 | 1p31.3 | *WLS/GNG12-AS1* | 1,01E-13 | **6,36E-07** | -7.29 | 2,41E-07 | SBP |
| 7 | rs2566755 | 1p31.3 | *WLS/GNG12-AS1* | 1,01E-13 | **6,36E-07** | -7.29 | 2,41E-07 | SBP |
| 7 | rs12407028* | 1p31.3 | *WLS/GNG12-AS1* | 7,26E-09 | **3,81E-05** | 5.67 | 4,50E-05 | SBP |
| 7 | rs2772300 | 1p31.3 | *WLS/GNG12-AS1* | 9,12E-10 | **5,58E-06** | -6 | 7,26E-06 | DBP |
| 8 | rs7554551 | 1p31.3 | *WLS/MIR1262/GNG12-AS1* | 3,69E-13 | **6,36E-07** | -7.12 | 2,41E-07 | SBP |
| 9 | rs11809524 | 1p21 | *COL11A1* | 8,21E-07 | **2,03E-03** | -4.83 | 1,34E-03 | SBP |
| 10 | rs681398 | 1q24.1 | *DNM3*/MIR3120* | 8,85E-06 | 1,20E-02 | -4.35 | 9,78E-03 | TG |
| 10 | rs479336* | 1q24.1 | *DNM3*/MIR3120* | 5,69E-08 | **2,52E-04** | -5.32 | 1,79E-04 | HDL |
| 11 | rs9309664 | 2p23 | *PPP1CB* | 7,55E-06 | 1,20E-02 | 4.39 | 8,22E-03 | HDL |
| 12 | rs7584262* | 2p21 | *PKDCC** | 7,84E-08 | **3,06E-04** | -5.26 | 4,88E-04 | LDL |
| 12 | rs2165239 | 2p21 | *PKDCC** | 2,66E-07 | **7,93E-04** | -5.04 | 9,04E-04 | T1D |
| 13 | rs17040773* | 2q12.1 | *ANAPC1** | 2,67E-06 | **4,96E-03** | 4.6 | 3,76E-03 | DBP |
| 14 | rs6710518 | 2q24-q31 | *GALNT3** | 8,70E-11 | **6,36E-07** | 6.35 | 6,18E-07 | SBP |
| 14 | rs1346004* | 2q24-q31 | *GALNT3** | 7,36E-11 | **6,36E-07** | 6.38 | 5,88E-07 | SBP |
| 15 | rs11675051 | 2q32.2 | *TMEM194B* | 1,46E-06 | **3,47E-03** | -4.72 | 1,56E-03 | SBP |
| 15 | rs3934784 | 2q32.2 | *TMEM194B* | 1,94E-06 | **4,15E-03** | -4.66 | 1,80E-03 | SBP |
| 15 | rs13005335 | 2q32.3-q33 | *NAB1* | 1,54E-06 | **3,47E-03** | -4.71 | 1,56E-03 | SBP |
| 16 | rs12995369 | 2q33.2 | *CDK15* | 1,07E-07 | **3,69E-04** | -5.2 | 2,80E-04 | SBP |
| 17 | rs7594560 | 2q33.3 | *METTL21A* | 3,42E-06 | **5,91E-03** | 4.55 | 3,74E-03 | HDL |
| 18 | rs416486 | 3p21 | *CTNNB1** | 4,57E-16 | **6,36E-07** | -7.96 | 2,41E-07 | SBP |
| 18 | rs1915925 | 3p21 | *CTNNB1** | 7,81E-06 | 1,20E-02 | 4.38 | 7,14E-03 | DBP |
| 18 | rs368006 | 3p21 | *CTNNB1** | 1,27E-14 | **6,36E-07** | 7.55 | 2,41E-07 | SBP |
| 18 | rs398993 | 3p21 | *CTNNB1** | 1,39E-14 | **6,36E-07** | 7.54 | 2,41E-07 | SBP |
| 18 | rs423170 | 3p21 | *CTNNB1** | 3,87E-16 | **6,36E-07** | 7.97 | 2,41E-07 | SBP |
| 18 | rs2024219 | 3p21 | *CTNNB1** | 4,63E-14 | **6,36E-07** | -7.39 | 2,41E-07 | SBP |
| 18 | rs7642431 | 3p21 | *CTNNB1** | 1,92E-12 | **6,36E-07** | -6.89 | 2,41E-07 | SBP |
| 18 | rs6599143 | 3p21 | *CTNNB1** | 8,53E-13 | **6,36E-07** | -7 | 2,41E-07 | SBP |
| 19 | rs11718013 | 3q13.31 | *KIAA2018** | 7,65E-06 | 1,20E-02 | -4.38 | 7,98E-03 | T1D |
| 19 | rs1026364* | 3q13.2 | *KIAA2018** | 1,22E-06 | **2,90E-03** | -4.75 | 2,52E-03 | SBP |
| 19 | rs12493635 | 3q13.2 | *KIAA2018** | 1,22E-06 | **2,90E-03** | -4.75 | 2,52E-03 | SBP |
| 20 | rs3755955* | 4p16.3 | *IDUA* | 2,21E-07 | **6,52E-04** | 5.07 | 6,22E-04 | DBP |
| 21 | rs1054627 | 4q21.1 | *IBSP* | 4,19E-10 | **2,35E-06** | 6.12 | 5,08E-06 | T2D |
| 21 | rs1471399 | 4q21.1 | *MEPE** | 1,17E-09 | **6,92E-06** | 5.96 | 6,62E-06 | LDL |
| 21 | rs1471403 | 4q21.1 | *MEPE** | 1,01E-09 | **5,58E-06** | 5.98 | 7,90E-06 | LDL |
| 21 | rs13130558 | 6p12 | *SPP1* | 1,21E-09 | **6,92E-06** | 5.95 | 1,00E-05 | SBP |
| 22 | rs17558396 | 5q14.3 | *MEF2C** | 4,10E-07 | **1,17E-03** | -4.96 | 7,69E-04 | SBP |
| 22 | rs700592 | 5q14.3 | *MEF2C** | 6,05E-07 | **1,70E-03** | 4.89 | 1,05E-03 | SBP |
| 22 | rs11958401 | 5q14.3 | *MEF2C** | 3,90E-06 | **7,05E-03** | 4.52 | 3,81E-03 | SBP |
| 22 | rs11952384 | 5q14.3 | *MEF2C** | 4,53E-09 | **2,48E-05** | 5.74 | 3,43E-05 | T2D |
| 22 | rs214137 | 5q14.3 | *MEF2C** | 3,48E-09 | **1,62E-05** | -5.79 | 3,43E-05 | T2D |
| 22 | rs169952 | 5q14.3 | *MEF2C** | 1,33E-11 | **6,36E-07** | -6.63 | 5,58E-07 | T2D |
| 22 | rs188515 | 5q14.3 | *MEF2C** | 1,55E-11 | **6,36E-07** | -6.6 | 5,58E-07 | T2D |
| 22 | rs6876387 | 5q14.3 | *MEF2C** | 1,08E-19 | **6,36E-07** | 8.89 | 5,06E-07 | T2D |
| 22 | rs6894139 | 5q14.3 | *MEF2C** | 2,99E-28 | **6,36E-07** | 10.79 | 5,51E-07 | LDL |
| 22 | rs1158464 | 5q14.3 | *MEF2C** | 3,56E-11 | **6,36E-07** | 6.48 | 2,41E-07 | SBP |
| 22 | rs10037512 | 5q14.3 | *MEF2C** | 4,27E-29 | **6,36E-07** | 10.97 | 5,87E-07 | T2D |
| 22 | rs1283614 | 5q14.3 | *MEF2C** | 3,28E-06 | **5,91E-03** | -4.56 | 3,10E-03 | SBP |
| 22 | rs1864180 | 5q14.3 | *MEF2C** | 2,05E-14 | **6,36E-07** | 7.49 | 4,21E-07 | T2D |
| 22 | rs10474292 | 5q14.3 | *MEF2C** | 2,75E-09 | **1,31E-05** | 5.82 | 1,43E-05 | SBP |
| 22 | rs13183402 | 5q14.3 | *MEF2C** | 3,85E-06 | **7,05E-03** | -4.52 | 5,83E-03 | SBP |
| 22 | rs7445369 | 5q14.3 | *MEF2C** | 5,79E-06 | 1,00E-02 | 4.44 | 8,66E-03 | DBP |
| 23 | rs4957742 | 5q21.2 | *RAB9BP1* | 2,98E-06 | **5,91E-03** | -4.58 | 6,27E-03 | DBP |
| 24 | rs1005886 | 6p22.2 | *CDKAL1** | 5,57E-06 | **8,40E-03** | 4.45 | 9,21E-03 | T1D |
| 24 | rs6456420 | 6p22.2 | *CDKAL1** | 5,57E-06 | **8,40E-03** | -4.45 | 9,16E-03 | TG |
| 24 | rs9466056* | 6p22.2 | *CDKAL1** | 9,01E-09 | **4,73E-05** | -5.63 | 6,89E-05 | LDL |
| 25 | rs10484759 | 6q22.32 | *CENPW* | 6,45E-06 | 1,00E-02 | 4.42 | 8,91E-03 | DBP |
| 25 | rs17563605 | 6q22.33 | *RSPO3* | 9,35E-07 | **2,43E-03** | 4.8 | 1,94E-03 | SBP |
| 25 | rs13204965* | 6q22.33 | *RSPO3* | 8,93E-07 | **2,43E-03** | 4.81 | 1,94E-03 | SBP |
| 26 | rs9479055 | 6q25.1 | *CCDC170** | 5,98E-11 | **6,36E-07** | -6.41 | 3,99E-07 | T1D |
| 26 | rs10872673 | 6q25.1 | *CCDC170** | 3,21E-12 | **6,36E-07** | -6.82 | 2,85E-07 | LDL |
| 26 | rs1856859 | 6q25.1 | *CCDC170** | 4,37E-08 | **1,69E-04** | 5.36 | 1,38E-04 | LDL |
| 26 | rs9479072 | 6q25.1 | *CCDC170** | 1,08E-13 | **6,36E-07** | -7.28 | 2,85E-07 | LDL |
| 26 | rs4869738 | 6q25.1 | *CCDC170** | 1,76E-06 | **3,47E-03** | -4.68 | 3,33E-03 | T1D |
| 26 | rs9479075 | 6q25.1 | *CCDC170** | 1,52E-15 | **6,36E-07** | -7.81 | 2,70E-07 | LDL |
| 26 | rs1871859 | 6q25.1 | *CCDC170** | 2,19E-09 | **1,06E-05** | 5.86 | 1,27E-05 | LDL |
| 26 | rs9478223 | 6q25.1 | *CCDC170** | 2,41E-06 | **4,96E-03** | 4.62 | 4,18E-03 | T2D |
| 26 | rs4869741 | 6q25.1 | *CCDC170** | 1,14E-14 | **6,36E-07** | 7.56 | 2,57E-07 | LDL |
| 26 | rs4869742* | 6q25.1 | *CCDC170** | 1,04E-14 | **6,36E-07** | 7.57 | 2,55E-07 | LDL |
| 26 | rs6925996 | 6q25.1 | *CCDC170** | 7,19E-13 | **6,36E-07** | 7.03 | 2,65E-07 | LDL |
| 26 | rs9383930 | 6q25.1 | *CCDC170** | 2,16E-09 | **1,06E-05** | 5.86 | 8,74E-06 | HDL |
| 26 | rs9397066 | 6q25.1 | *CCDC170** | 3,44E-09 | **1,62E-05** | 5.79 | 1,53E-05 | SBP |
| 26 | rs1340874 | 6q25.1 | *CCDC170** | 1,20E-08 | **5,87E-05** | 5.58 | 4,57E-05 | HDL |
| 26 | rs6929137 | 6q25.1 | *CCDC170** | 4,55E-12 | **6,36E-07** | 6.78 | 3,83E-07 | SBP |
| 26 | rs3734804 | 6q25.1 | *CCDC170** | 3,09E-14 | **6,36E-07** | 7.44 | 4,10E-07 | T2D |
| 26 | rs6904261 | 6q25.1 | *CCDC170** | 2,42E-09 | **1,31E-05** | 5.84 | 9,98E-06 | SBP |
| 26 | rs3734806 | 6q25.1 | *CCDC170** | 7,49E-12 | **6,36E-07** | 6.71 | 4,12E-07 | SBP |
| 26 | rs10872676 | 6q25.1 | *CCDC170** | 8,10E-14 | **6,36E-07** | 7.31 | 1,36E-07 | HDL |
| 26 | rs7752591 | 6q25.1 | *CCDC170** | 3,04E-13 | **6,36E-07** | 7.14 | 4,13E-07 | T2D |
| 26 | rs865898 | 6q25.1 | *CCDC170** | 4,33E-12 | **6,36E-07** | 6.78 | 4,13E-07 | T2D |
| 26 | rs712219 | 6q24-q27 | *ESR1* | 5,48E-13 | **6,36E-07** | 7.06 | 3,42E-07 | SBP |
| 26 | rs851970 | 6q24-q27 | *ESR1* | 9,63E-13 | **6,36E-07** | 6.99 | 3,05E-07 | SBP |
| 26 | rs980281 | 6q24-q27 | *ESR1* | 2,90E-07 | **9,66E-04** | 5.02 | 1,09E-03 | T1D |
| 26 | rs6557164 | 6q24-q27 | *ESR1* | 7,58E-06 | 1,20E-02 | 4.38 | 7,14E-03 | DBP |
| 26 | rs851993 | 6q24-q27 | *ESR1* | 1,62E-12 | **6,36E-07** | 6.92 | 4,31E-07 | T1D |
| 26 | rs3020333 | 6q24-q27 | *ESR1* | 3,49E-15 | **6,36E-07** | -7.71 | 1,31E-07 | HDL |
| 26 | rs2982570 | 6q24-q27 | *ESR1* | 2,23E-14 | **6,36E-07** | -7.48 | 1,30E-07 | HDL |
| 26 | rs851984 | 6q24-q27 | *ESR1* | 9,86E-13 | **6,36E-07** | -6.99 | 1,32E-07 | HDL |
| 26 | rs851983 | 6q24-q27 | *ESR1* | 1,29E-12 | **6,36E-07** | -6.95 | 1,34E-07 | HDL |
| 26 | rs851980 | 6q24-q27 | *ESR1* | 7,97E-08 | **3,06E-04** | -5.26 | 1,13E-04 | HDL |
| 26 | rs6899458 | 6q24-q27 | *ESR1* | 7,32E-06 | 1,20E-02 | -4.39 | 3,11E-03 | HDL |
| 26 | rs2982554 | 6q24-q27 | *ESR1* | 2,57E-11 | **6,36E-07** | -6.53 | 1,35E-07 | HDL |
| 26 | rs1999805 | 6q24-q27 | *ESR1* | 4,37E-11 | **6,36E-07** | -6.46 | 1,52E-07 | HDL |
| 26 | rs1124674 | 6q24-q27 | *ESR1* | 1,35E-08 | **5,87E-05** | -5.56 | 3,77E-05 | HDL |
| 26 | rs2504070 | 6q24-q27 | *ESR1* | 7,07E-06 | 1,00E-02 | -4.4 | 6,13E-03 | SBP |
| 26 | rs1890010 | 6q24-q27 | *ESR1* | 3,42E-08 | **1,37E-04** | -5.4 | 1,05E-04 | SBP |
| 26 | rs2504069 | 6q24-q27 | *ESR1* | 3,63E-08 | **1,69E-04** | -5.39 | 1,05E-04 | SBP |
| 26 | rs2504063 | 6q24-q27 | *ESR1* | 8,35E-09 | **3,81E-05** | -5.64 | 2,95E-05 | HDL |
| 27 | rs6583337 | 7p22.3 | *FAM20C* | 3,30E-06 | **5,91E-03** | 4.56 | 3,38E-03 | LDL |
| 28 | rs1721385 | 7p14.1 | *EPDR1* | 1,65E-06 | **3,47E-03** | -4.69 | 2,50E-03 | DBP |
| 28 | rs1717731 | 7p14.1 | *EPDR1* | 1,73E-06 | **3,47E-03** | -4.68 | 2,50E-03 | DBP |
| 28 | rs1524058 | 7p14-p13 | *STARD3NL** | 2,55E-06 | **4,96E-03** | -4.61 | 2,55E-03 | SBP |
| 28 | rs4576333 | 7p14-p13 | *STARD3NL** | 1,66E-06 | **3,47E-03** | -4.69 | 1,81E-03 | DBP |
| 29 | rs2282930 | 7p12.2 | *GRB10* | 5,20E-06 | **8,40E-03** | 4.46 | 7,20E-03 | TG |
| 30 | rs12154661 | 7q21.3 | *C7orf76* | 3,10E-06 | **5,91E-03** | 4.57 | 6,28E-03 | T1D |
| 31 | rs2724034 | 7q21.3 | *C7orf76* | 5,26E-06 | **8,40E-03** | 4.46 | 9,94E-03 | T2D |
| 32 | rs3094750 | 7q21.3 | *C7orf76* | 7,26E-12 | **6,36E-07** | 6.71 | 4,61E-07 | HDL |
| 32 | rs3113040 | 7q21.3 | *C7orf76* | 1,06E-11 | **6,36E-07** | 6.66 | 4,05E-07 | HDL |
| 32 | rs10953178 | 7q21.3 | *C7orf76* | 3,75E-11 | **6,36E-07** | -6.48 | 3,53E-07 | HDL |
| 32 | rs7781370 | 7q21.3 | *C7orf76* | 7,49E-21 | **6,36E-07** | -9.17 | 4,74E-07 | LDL |
| 32 | rs4132567 | 7q21.3 | *C7orf76* | 5,06E-06 | **8,40E-03** | -4.47 | 9,34E-03 | HDL |
| 32 | rs4427101 | 7q21.3 | *C7orf76* | 6,71E-21 | **6,36E-07** | -9.18 | 5,23E-07 | HDL |
| 32 | rs4296976 | 7q21.3 | *C7orf76* | 1,94E-08 | **8,99E-05** | -5.5 | 1,10E-04 | HDL |
| 32 | rs4566970 | 7q21.3 | *C7orf76* | 1,94E-08 | **8,99E-05** | -5.5 | 1,24E-04 | HDL |
| 32 | rs10464592 | 7q21.3 | *SHFM1* | 4,28E-10 | **2,35E-06** | 6.11 | 4,07E-06 | SBP |
| 32 | rs2272224 | 7q21.3 | *SHFM1* | 4,85E-07 | **1,42E-03** | -4.93 | 1,12E-03 | DBP |
| 32 | rs13310130 | 7q21.3 | *SHFM1* | 4,33E-09 | **2,00E-05** | 5.75 | 2,12E-05 | DBP |
| 32 | rs10499928 | 7q21.3 | *SHFM1* | 7,88E-06 | 1,20E-02 | 4.38 | 7,89E-03 | SBP |
| 33 | rs2908007 | 7q31 | *WNT16** | 1,60E-07 | **5,37E-04** | -5.13 | 6,17E-04 | HDL |
| 33 | rs3779381 | 7q31 | *WNT16** | 5,10E-13 | **6,36E-07** | -7.07 | 2,41E-07 | SBP |
| 33 | rs2908004 | 7q31 | *WNT16** | 3,79E-11 | **6,36E-07** | -6.48 | 4,47E-07 | SBP |
| 33 | rs3801382 | 7q22.1-q31.1 | *FAM3C* | 2,05E-14 | **6,36E-07** | -7.49 | 2,41E-07 | SBP |
| 33 | rs917727 | 7q22.1-q31.1 | *FAM3C* | 1,04E-14 | **6,36E-07** | -7.57 | 2,41E-07 | SBP |
| 33 | rs7776725 | 7q22.1-q31.1 | *FAM3C* | 1,59E-14 | **6,36E-07** | -7.52 | 2,41E-07 | SBP |
| 34 | rs7812088* | 7q36.1 | *ABCF2* | 7,25E-07 | **2,03E-03** | -4.85 | 1,44E-03 | HDL |
| 34 | rs7781265 | 7q35-q36 | *SMARCD3* | 8,16E-07 | **2,03E-03** | -4.83 | 1,61E-03 | HDL |
| 35 | rs1670357 | 7q36 | *PTPRN2/MIR595* | 1,73E-06 | **3,47E-03** | -4.68 | 1,90E-03 | SBP |
| 35 | rs1670346 | 7q36 | *PTPRN2/MIR595* | 1,73E-06 | **3,47E-03** | -4.68 | 1,80E-03 | SBP |
| 35 | rs1733125 | 7q36 | *PTPRN2/MIR595* | 1,93E-06 | **4,15E-03** | -4.66 | 2,13E-03 | SBP |
| 36 | rs13255886 | 8q13.3 | *LACTB2* | 2,42E-07 | **7,93E-04** | 5.06 | 6,22E-04 | DBP |
| 36 | rs7017914* | 8q13.3 | *XKR9* | 2,03E-07 | **6,52E-04** | 5.09 | 5,85E-04 | SBP |
| 36 | rs1596566 | 8q13.3 | *XKR9* | 2,42E-07 | **7,93E-04** | 5.06 | 6,22E-04 | DBP |
| 36 | rs6472551 | 8q13.3 | *XKR9* | 2,64E-07 | **7,93E-04** | 5.04 | 6,99E-04 | SBP |
| 37 | rs980299 | 8q13.3 | *EYA1* | 1,18E-07 | **4,45E-04** | 5.19 | 3,39E-04 | HDL |
| 38 | rs13272568 | 8q21.11 | *PKIA* | 1,29E-06 | **2,90E-03** | 4.74 | 2,52E-03 | SBP |
| 39 | rs16891598 | 8q24 | *TNFRSF11B** | 1,92E-06 | **4,15E-03** | 4.66 | 3,38E-03 | SBP |
| 39 | rs6651219 | 8q24 | *TNFRSF11B** | 2,07E-06 | **4,15E-03** | 4.65 | 3,38E-03 | SBP |
| 39 | rs16891617 | 8q24 | *TNFRSF11B** | 3,29E-06 | **5,91E-03** | 4.56 | 3,28E-03 | SBP |
| 39 | rs4407910 | 8q24 | *TNFRSF11B** | 3,63E-12 | **6,36E-07** | -6.81 | 3,26E-07 | T1D |
| 39 | rs4355801 | 8q24 | *TNFRSF11B** | 1,95E-12 | **6,36E-07** | -6.89 | 3,26E-07 | T1D |
| 39 | rs4876868 | 8q24 | *TNFRSF11B** | 2,82E-08 | **1,37E-04** | -5.44 | 1,91E-04 | HDL |
| 39 | rs11573871 | 8q24 | *TNFRSF11B** | 2,59E-06 | **4,96E-03** | -4.6 | 3,48E-03 | HDL |
| 39 | rs3102735 | 8q24 | *TNFRSF11B** | 2,94E-10 | **1,89E-06** | 6.17 | 2,83E-06 | SBP |
| 39 | rs12386806 | 8q24 | *TNFRSF11B** | 2,48E-10 | **1,52E-06** | 6.2 | 2,68E-06 | T2D |
| 39 | rs10505348 | 8q24 | *TNFRSF11B** | 5,84E-15 | **6,36E-07** | 7.65 | 3,26E-07 | T1D |
| 39 | rs1385499 | 8q24 | *TNFRSF11B** | 8,95E-16 | **6,36E-07** | 7.87 | 4,13E-07 | T2D |
| 39 | rs1564860 | 8q24 | *TNFRSF11B** | 9,57E-17 | **6,36E-07** | 8.14 | 4,81E-07 | T2D |
| 39 | rs1825511 | 8q24 | *TNFRSF11B** | 1,57E-08 | **7,27E-05** | 5.54 | 1,27E-04 | LDL |
| 39 | rs6469794 | 8q24 | *TNFRSF11B** | 3,96E-12 | **6,36E-07** | 6.8 | 3,26E-07 | T1D |
| 39 | rs7013203 | 8q24 | *TNFRSF11B** | 4,33E-12 | **6,36E-07** | 6.78 | 3,26E-07 | T1D |
| 39 | rs1586274 | 8q23-q24.1 | *COLEC10* | 1,87E-14 | **6,36E-07** | 7.5 | 5,27E-07 | T2D |
| 39 | rs2326193 | 8q23-q24.1 | *COLEC10* | 4,33E-12 | **6,36E-07** | 6.78 | 3,26E-07 | T1D |
| 39 | rs7016585 | 8q23-q24.1 | *COLEC10* | 2,87E-11 | **6,36E-07** | 6.52 | 3,26E-07 | T1D |
| 39 | rs1385509 | 8q23-q24.1 | *COLEC10* | 1,12E-06 | **2,43E-03** | 4.77 | 1,50E-03 | HDL |
| 40 | rs567960 | 9q31 | *KLF4* | 6,82E-06 | 1,00E-02 | 4.41 | 5,84E-03 | DBP |
| 40 | rs665556 | 9q31 | *KLF4* | 6,68E-06 | 1,00E-02 | 4.41 | 5,84E-03 | DBP |
| 41 | rs7466269 | 9q34.11 | *FUBP3** | 2,26E-08 | **1,11E-04** | 5.48 | 5,25E-05 | HDL |
| 42 | rs12262178 | 10q11.2 | *MBL2** | 4,63E-06 | **8,40E-03** | 4.49 | 6,56E-03 | DBP |
| 42 | rs12262251 | 10q11.2 | *MBL2** | 5,18E-06 | **8,40E-03** | 4.46 | 6,42E-03 | DBP |
| 42 | rs11003047 | 10q11.2 | *MBL2** | 4,76E-06 | **8,40E-03** | 4.48 | 6,24E-03 | DBP |
| 43 | rs1385162 | 11p15.3 | *SOX6** | 1,08E-15 | **6,36E-07** | 7.85 | 4,57E-07 | T2D |
| 43 | rs7117858 | 11p15.3 | *SOX6** | 2,95E-17 | **6,36E-07** | 8.27 | 4,37E-07 | T2D |
| 43 | rs9787942 | 11p15.3 | *SOX6** | 6,45E-17 | **6,36E-07** | 8.18 | 4,22E-07 | T2D |
| 43 | rs4757353 | 11p15.3 | *SOX6** | 1,18E-16 | **6,36E-07** | 8.11 | 4,22E-07 | T2D |
| 43 | rs10766280 | 11p15.3 | *SOX6** | 1,59E-16 | **6,36E-07** | 8.08 | 4,10E-07 | T2D |
| 43 | rs7933516 | 11p15.3 | *SOX6** | 3,37E-15 | **6,36E-07** | 7.71 | 4,21E-07 | T2D |
| 43 | rs16931831 | 11p15.3 | *SOX6** | 1,19E-16 | **6,36E-07** | -8.11 | 4,37E-07 | T2D |
| 44 | rs10832519 | 11p15.3 | *SOX6** | 4,28E-07 | **1,17E-03** | -4.95 | 6,97E-04 | HDL |
| 44 | rs11023718 | 11p15.3 | *SOX6** | 3,19E-07 | **9,66E-04** | -5.01 | 5,84E-04 | HDL |
| 45 | rs1949481 | 11p15.3 | *SOX6** | 3,10E-07 | **9,66E-04** | 5.01 | 7,17E-04 | DBP |
| 46 | rs12418348 | 11p15.3 | *SOX6** | 1,66E-06 | **3,47E-03** | 4.69 | 2,48E-03 | HDL |
| 46 | rs7924783 | 11p15.3 | *SOX6** | 1,66E-06 | **3,47E-03** | 4.69 | 2,15E-03 | HDL |
| 47 | rs2021807 | 11p14.1 | *DCDC5* | 5,03E-07 | **1,42E-03** | -4.92 | 1,56E-03 | DBP |
| 47 | rs911268 | 11p14.1 | *DCDC5* | 4,74E-07 | **1,42E-03** | -4.93 | 1,59E-03 | LDL |
| 47 | rs10767877 | 11p14.1 | *DCDC5* | 4,62E-07 | **1,42E-03** | 4.94 | 1,39E-03 | DBP |
| 47 | rs1028643 | 11p14.1 | *DCDC5* | 5,22E-07 | **1,42E-03** | 4.91 | 1,39E-03 | DBP |
| 47 | rs273567 | 11p14.1 | *DCDC5* | 5,96E-07 | **1,70E-03** | 4.89 | 1,83E-03 | DBP |
| 47 | rs273608 | 11p14.1 | *DCDC5* | 8,55E-07 | **2,03E-03** | 4.82 | 2,05E-03 | HDL |
| 48 | rs7932354* | 11p11.2 | *ARHGAP1** | 1,15E-08 | **5,87E-05** | 5.59 | 2,02E-05 | HDL |
| 48 | rs6485690 | 11p11.2 | *SNORD67* | 1,49E-07 | **5,37E-04** | 5.15 | 1,59E-04 | HDL |
| 49 | rs600231 | 11q13.1 | *MALAT1* | 7,75E-06 | 1,20E-02 | -4.38 | 7,60E-03 | SBP |
| 50 | rs608343 | 11q13.4 | *LRP5** | 3,84E-07 | **1,17E-03** | 4.97 | 1,12E-03 | SBP |
| 51 | rs7304170 | 12p11.22 | *KLHL42* | 1,04E-06 | **2,43E-03** | -4.78 | 2,96E-03 | T2D |
| 51 | rs258415 | 12p11.22 | *KLHL42* | 3,55E-08 | **1,69E-04** | -5.4 | 1,43E-04 | SBP |
| 52 | rs2016266* | 12q13.13 | *SP7** | 2,74E-07 | **7,93E-04** | 5.03 | 5,41E-04 | HDL |
| 52 | rs7310771 | 12q13 | *ATF7* | 7,44E-07 | **2,03E-03** | 4.85 | 8,24E-04 | LDL |
| 52 | rs10783588 | 12q13 | *ATF7* | 8,99E-07 | **2,43E-03** | 4.81 | 1,03E-03 | HDL |
| 52 | rs7970141 | 12q13 | *ATF7* | 7,49E-07 | **2,03E-03** | 4.85 | 1,10E-03 | HDL |
| 53 | rs11614913 | 12q13.13 | *MIR196A2* | 4,20E-08 | **1,69E-04** | 5.37 | 1,25E-04 | SBP |
| 54 | rs10746070 | 12q23.3 | *RIC8B* | 2,14E-06 | **4,15E-03** | -4.64 | 3,13E-03 | HDL |
| 54 | rs759603 | 12q23.3 | *RIC8B* | 2,14E-06 | **4,15E-03** | -4.64 | 3,13E-03 | HDL |
| 54 | rs6539289 | 12q23.3 | *RIC8B* | 2,27E-06 | **4,96E-03** | -4.63 | 3,31E-03 | HDL |
| 54 | rs1444581 | 12q23.3 | *RIC8B* | 2,27E-06 | **4,96E-03** | -4.63 | 3,46E-03 | T2D |
| 55 | rs912100 | 13q | *AKAP11** | 1,86E-05 | 2,46E-02 | 4.19 | 7,10E-03 | HDL |
| 55 | rs9533090* | 13q | *AKAP11** | 3,96E-11 | **6,36E-07** | 6.47 | 6,17E-07 | T2D |
| 55 | rs9594738 | 13q | *AKAP11** | 5,17E-11 | **6,36E-07** | 6.43 | 7,28E-07 | T2D |
| 55 | rs17638544 | 13q | *AKAP11** | 2,81E-06 | **4,96E-03** | -4.59 | 3,18E-03 | DBP |
| 55 | rs10507508 | 13q | *AKAP11** | 2,74E-06 | **4,96E-03** | -4.59 | 2,61E-03 | DBP |
| 56 | rs1286147 | 14q31-q32.1 | *RPS6KA5** | 7,47E-09 | **3,81E-05** | -5.66 | 5,16E-05 | T2D |
| 57 | rs3783394 | 14q32.3 | *MARK3** | 7,96E-07 | **2,03E-03** | 4.83 | 1,20E-03 | DBP |
| 57 | rs6575984 | 14q32.3 | *MARK3** | 8,99E-07 | **2,43E-03** | -4.81 | 9,64E-04 | HDL |
| 57 | rs11623869* | 14q32.3 | *MARK3** | 7,49E-07 | **2,03E-03** | 4.85 | 1,20E-03 | DBP |
| 57 | rs7152202 | 14q32.3 | *MARK3** | 7,49E-07 | **2,03E-03** | 4.85 | 1,20E-03 | DBP |
| 57 | rs7158144 | 14q32.3 | *MARK3** | 7,49E-07 | **2,03E-03** | 4.85 | 1,20E-03 | DBP |
| 57 | rs7158822 | 14q32.3 | *MARK3** | 7,49E-07 | **2,03E-03** | 4.85 | 1,20E-03 | DBP |
| 58 | rs4646 | 15q21 | *CYP19A1* | 5,53E-06 | **8,40E-03** | -4.45 | 4,90E-03 | SBP |
| 58 | rs7175531 | 15q21 | *CYP19A1* | 2,30E-06 | **4,96E-03** | -4.63 | 4,82E-03 | HDL |
| 58 | rs4775936 | 15q21 | *MIR4713* | 3,41E-06 | **5,91E-03** | -4.55 | 4,77E-03 | T1D |
| 58 | rs10851498 | 15q21 | *MIR4713* | 2,73E-06 | **4,96E-03** | -4.59 | 4,45E-03 | TG |
| 59 | rs9921222* | 16p13.3 | *AXIN1*/LUC7L* | 1,37E-07 | **4,45E-04** | 5.16 | 6,90E-04 | DBP |
| 60 | rs13336428* | 16p13.3 | *PTX4* | 1,60E-07 | **5,37E-04** | 5.13 | 8,55E-04 | T1D |
| 61 | rs3198697 | 16p13.11 | *PDXDC1* | 1,01E-05 | 1,44E-02 | 4.32 | 5,00E-03 | HDL |
| 62 | rs1566045* | 16q12.1 | *SALL1* | 1,76E-12 | **6,36E-07** | -6.91 | 3,26E-07 | T1D |
| 63 | rs10048146* | 16q24 | *FOXL1* | 6,95E-08 | **2,52E-04** | 5.28 | 2,92E-04 | HDL |
| 64 | rs4790881* | 17p13.3 | *SMG6** | 8,24E-09 | **3,81E-05** | -5.64 | 2,93E-05 | SBP |
| 64 | rs8077194 | 17p13.3 | *SMG6** | 1,22E-08 | **5,87E-05** | -5.58 | 3,63E-05 | SBP |
| 65 | rs1877632 | 17q12-q21 | *SOST** | 2,98E-09 | **1,62E-05** | -5.81 | 1,10E-05 | HDL |
| 66 | rs227580 | 17q21.31 | *C17orf53** | 1,95E-11 | **6,36E-07** | -6.57 | 2,41E-07 | SBP |
| 66 | rs227584* | 17q21.31 | *C17orf53** | 1,33E-11 | **6,36E-07** | -6.63 | 2,41E-07 | SBP |
| 66 | rs730228 | 17q21.31 | *C17orf53** | 2,11E-11 | **6,36E-07** | -6.56 | 3,53E-07 | HDL |
| 66 | rs7207464 | 17q21.31 | *ASB16* | 1,43E-11 | **6,36E-07** | -6.62 | 2,41E-07 | SBP |
| 66 | rs721769 | 17q21.31 | *ASB16-AS1* | 2,11E-11 | **6,36E-07** | -6.56 | 2,41E-07 | SBP |
| 67 | rs199533 | 17q21 | *NSF* | 5,77E-06 | 1,00E-02 | 4.44 | 5,84E-03 | DBP |
| 67 | rs199529 | 17q21 | *NSF* | 2,39E-06 | **4,96E-03** | 4.62 | 2,55E-03 | SBP |
| 68 | rs10491193 | 17q24.3 | *SOX9* | 9,72E-06 | 1,44E-02 | -4.33 | 8,54E-03 | HDL |
| 68 | rs12709255 | 17q24.3 | *SOX9* | 2,50E-08 | **1,11E-04** | 5.46 | 1,86E-04 | SBP |
| 68 | rs7217932* | 17q24.3 | *SOX9* | 1,88E-08 | **8,99E-05** | 5.51 | 1,26E-04 | T2D |
| 68 | rs11650567 | 17q24.3 | *SOX9* | 2,17E-08 | **8,99E-05** | 5.48 | 1,52E-04 | SBP |
| 69 | rs4796995* | 18p11.21 | *FAM210A** | 1,96E-06 | **4,15E-03** | 4.66 | 2,26E-03 | SBP |
| 70 | rs2957153 | 18q22.1 | *TNFRSF11A** | 2,54E-06 | **4,96E-03** | -4.61 | 3,07E-03 | DBP |
| 71 | rs11659752 | 18q23 | *NFATC1* | 4,79E-06 | **8,40E-03** | -4.48 | 8,20E-03 | LDL |
| 71 | rs8090312 | 18q23 | *NFATC1* | 4,54E-06 | **8,40E-03** | -4.49 | 6,40E-03 | T1D |
| 71 | rs11660128 | 18q23 | *NFATC1* | 3,42E-06 | **5,91E-03** | -4.55 | 7,17E-03 | TG |
| 72 | rs3760891 | 19q13.12 | *GPATCH1** | 2,78E-06 | **4,96E-03** | -4.59 | 4,75E-03 | LDL |
| 72 | rs2287679 | 19q13.12 | *GPATCH1** | 2,63E-06 | **4,96E-03** | -4.6 | 4,46E-03 | LDL |
| 72 | rs10416265 | 19q13.12 | *GPATCH1** | 2,47E-06 | **4,96E-03** | -4.61 | 4,07E-03 | T1D |
| 73 | rs6514116 | 20p12.1-p11.23 | *JAG1** | 5,29E-08 | **2,07E-04** | 5.33 | 3,06E-04 | SBP |
| 73 | rs6040061 | 20p12.1-p11.23 | *JAG1** | 5,20E-08 | **2,07E-04** | 5.33 | 2,71E-04 | T1D |
| 74 | rs2267004 | 22q11.2 | *RTDR1/GNAZ* | 3,21E-06 | **5,91E-03** | 4.56 | 7,17E-03 | TG |
| 74 | rs1051875 | 22q11.2 | *RTDR1/GNAZ* | 4,57E-06 | **8,40E-03** | 4.49 | 5,95E-03 | TG |
| 74 | rs756632 | 22q11.2 | *RTDR1/GNAZ* | 3,39E-06 | **5,91E-03** | -4.55 | 4,75E-03 | HDL |
| 74 | rs13055979 | 22q11.2 | *RTDR1/GNAZ* | 6,70E-06 | 1,00E-02 | 4.41 | 5,84E-03 | DBP |
| 74 | rs4820539 | 22q11.2 | *RAB36* | 3,06E-06 | **5,91E-03** | 4.57 | 7,44E-03 | HDL |
| Independent complex or single gene loci (LD-r^2^ < 0.2) with SNP(s) with a conditional FDR (condFDR) < 0.01 in bone mineral density (BMD, femoral neck) given the association in other phenotypes. We defined the most significant BMD SNP in each LD block based on the minimum condFDR (min cond FDR) for each phenotype. The second phenotype which provides the minimal FDR signal (Driving phenotype) is listed. All loci with SNPs with condFDR < 0.01 were used to define the number of the loci. The following abbreviations were used: type 1 diabetes (T1D), type 2 diabetes (T2D), systolic blood pressure (SBP), diastolic blood pressure (DBP), high density lipoprotein (HDL), low density lipoprotein (LDL), triglycerides (TG), waist hip ratio (WHR), chromosome location (Map Loc.). Shaded r values represent nominally significant (p<0.05) Pearson correlations (age and BMI adjusted FN BMD vs. Affymetrix signal values). SNPs and Genes previously reported to associate with BMD are marked with stars (*). BMD FDR values < 0.01 are in bold. Wald stats: z-score transformed from p values. | | | | | | | | |
