# Supplementary material for: Genetic Sharing with Cardiovascular Disease Risk Factors and Diabetes Reveals Novel Bone Mineral Density Loci
Source: PLoS One. 2015 Dec 22;10(12):e0144531. doi: 10.1371/journal.pone.0144531 (PMC4687843; doi:10.1371/journal.pone.0144531)
Supplement: S3 Table — (DOCX) [file pone.0144531.s011.docx]

| **S3 Table. Identified loci containing novel SNPs or genes associated with lumbar spine BMD** | | | | | | | | |
| --- | --- | --- | --- | --- | --- | --- | --- | --- |
| **Locus#** | **SNP** | **Map loc.** | **Gene symbol** | **BMD**  **p-value** | **BMD FDR** | **Wald stats** | **min cond FDR** | **Driving phenotype** |
| 1 | rs12079653 | 1p36.23 | *RERE* | 2,56E-05 | 2,55E-02 | -4.11 | 9,54E-03 | LDL |
| 2 | rs12136689 | 1p36.23 | *RERE* | 9,75E-06 | 1,19E-02 | 4.32 | 6,88E-03 | DBP |
| 7 | rs1889830 | 1p32-p31 | *MACF1* | 1,18E-05 | 1,44E-02 | 4.28 | 4,91E-03 | HDL |
| 7 | rs2275187 | 1p32-p31 | *MACF1* | 1,01E-05 | 1,19E-02 | 4.31 | 4,91E-03 | HDL |
| 8 | rs2566784 | 1p31.3 | *WLS/GNG12-AS1* | 6,67E-08 | **1,72E-04** | -5.27 | 1,62E-04 | SBP |
| 10 | rs7554551 | 1p31.3 | *WLS/MIR1262/GNG12-AS1* | 1,07E-15 | **5,10E-07** | -7.83 | 3,30E-07 | SBP |
| 11 | rs2273368 | 1p13 | *WNT2B* | 1,22E-05 | 1,44E-02 | -4.27 | 4,30E-03 | DBP |
| 11 | rs17030613 | 1p13.2 | *CAPZA1* | 1,14E-05 | 1,44E-02 | -4.28 | 4,30E-03 | DBP |
| 12 | rs12120297 | 1q41 | *SUSD4* | 1,73E-06 | **2,47E-03** | 4.67 | 2,23E-03 | LDL |
| 13 | rs780110 | 2p23.3 | *IFT172* | 3,83E-05 | 3,64E-02 | -4.02 | 7,67E-03 | TG |
| 14 | rs4389358 | 2p16.2 | *C2orf73* | 2,26E-05 | 2,55E-02 | 4.14 | 6,60E-03 | HDL |
| 16 | rs17049689 | 2p16.1 | *FANCL* | 1,40E-05 | 1,44E-02 | -4.24 | 5,79E-03 | DBP |
| 19 | rs7636399 | 3p23 | *SUSD5* | 4,11E-06 | **5,45E-03** | 4.5 | 4,68E-03 | LDL |
| 19 | rs9861369 | 3p23 | *SUSD5* | 5,13E-06 | **6,62E-03** | 4.45 | 3,40E-03 | DBP |
| 19 | rs7630970 | 3p23 | *SUSD5* | 4,93E-06 | **6,62E-03** | 4.46 | 3,11E-03 | DBP |
| 21 | rs6599179 | 3p22.1 | *ULK4* | 6,83E-06 | **8,05E-03** | 4.39 | 3,90E-03 | WHR |
| 21 | rs900569 | 3p22.1 | *ULK4* | 1,81E-05 | 2,11E-02 | 4.18 | 7,60E-03 | DBP |
| 21 | rs9856088 | 3p22.1 | *ULK4* | 5,94E-06 | **8,05E-03** | 4.42 | 3,18E-03 | WHR |
| 21 | rs1717017 | 3p22.1 | *ULK4* | 8,06E-06 | **9,79E-03** | 4.36 | 3,90E-03 | WHR |
| 21 | rs1716655 | 3p22.1 | *ULK4* | 9,12E-06 | 1,19E-02 | -4.33 | 2,95E-03 | DBP |
| 21 | rs9838537 | 3p22.1 | *ULK4* | 2,73E-06 | **3,69E-03** | -4.58 | 1,30E-03 | WHR |
| 22 | rs11720264 | 3p21.31 | *BSN-AS2* | 1,29E-05 | 1,44E-02 | -4.26 | 5,12E-03 | WHR |
| 22 | rs11919311 | 3p21.31 | *BSN* | 3,51E-06 | **4,49E-03** | -4.53 | 1,88E-03 | SBP |
| 22 | rs1060962 | 3p21.31 | *BSN* | 4,41E-06 | **5,45E-03** | -4.48 | 2,33E-03 | SBP |
| 22 | rs4855881 | 3p21 | *APEH* | 3,93E-06 | **5,45E-03** | -4.5 | 1,88E-03 | SBP |
| 24 | rs6787138 | 3q27 | *ATP11B* | 8,66E-06 | **9,79E-03** | -4.34 | 3,90E-03 | WHR |
| 25 | rs6599389 | 4p16.3 | *TMEM175* | 1,30E-05 | 1,44E-02 | 4.25 | 4,28E-03 | WHR |
| 27 | rs11729056 | 4q13.3 | *AREG* | 1,33E-05 | 1,44E-02 | -4.25 | 5,18E-03 | DBP |
| 28 | rs11942753 | 4q21.3 | *DSPP* | 1,31E-05 | 1,44E-02 | 4.25 | 5,83E-03 | WHR |
| 30 | rs4862810 | 4q35.2 | *ZFP42* | 6,97E-06 | **8,05E-03** | -4.39 | 6,68E-03 | T2D |
| 31 | rs3822469 | 5p13 | *SLC1A3* | 8,23E-06 | **9,79E-03** | -4.35 | 6,14E-03 | DBP |
| 36 | rs6465508 | 7q21.3 | *C7orf76* | 3,17E-16 | **5,10E-07** | -7.97 | 2,51E-07 | LDL |
| 36 | rs10808100 | 7q21.3 | *C7orf76* | 3,48E-16 | **5,10E-07** | -7.96 | 2,74E-07 | LDL |
| 36 | rs7781370 | 7q21.3 | *C7orf76* | 3,81E-16 | **5,10E-07** | -7.95 | 1,91E-07 | LDL |
| 36 | rs4370463 | 7q21.3 | *C7orf76* | 3,81E-16 | **5,10E-07** | -7.95 | 2,30E-07 | LDL |
| 36 | rs10085588 | 7q21.3 | *C7orf76* | 2,54E-15 | **5,10E-07** | -7.72 | 2,51E-07 | LDL |
| 36 | rs6971293 | 7q21.3 | *C7orf76* | 3,48E-16 | **5,10E-07** | -7.96 | 2,51E-07 | LDL |
| 36 | rs2272224 | 7q21.3 | *SHFM1* | 1,15E-08 | **4,23E-05** | -5.57 | 3,73E-05 | T1D |
| 36 | rs2922927 | 7q21.3 | *SHFM1* | 1,31E-06 | **2,00E-03** | -4.72 | 1,27E-03 | LDL |
| 36 | rs7786855 | 7q21.3 | *SHFM1* | 2,47E-06 | **3,69E-03** | 4.6 | 2,26E-03 | DBP |
| 38 | rs10274324 | 7q31.31 | *CPED1* | 2,43E-06 | **3,69E-03** | -4.6 | 1,25E-03 | WHR |
| 38 | rs798917 | 7q31.31 | *CPED1* | 2,05E-06 | **3,02E-03** | -4.63 | 1,25E-03 | WHR |
| 38 | rs798915 | 7q31.31 | *CPED1* | 1,94E-06 | **3,02E-03** | -4.64 | 1,25E-03 | WHR |
| 38 | rs2691032 | 7q31.31 | *CPED1* | 1,74E-06 | **2,47E-03** | -4.67 | 1,25E-03 | WHR |
| 38 | rs2536150 | 7q31.31 | *CPED1* | 2,16E-06 | **3,02E-03** | 4.62 | 2,09E-03 | LDL |
| 41 | rs980695 | 8q23-q24.1 | *COLEC10* | 2,07E-06 | **3,02E-03** | -4.63 | 3,54E-03 | LDL |
| 42 | rs10956415 | 8q24 | *MIR1208* | 5,05E-06 | **6,62E-03** | -4.45 | 6,49E-03 | WHR |
| 43 | rs592203 | 9p24.1-p23 | *PTPRD/SNORD27* | 1,93E-05 | **2,11E-02** | 4.17 | 8,12E-03 | SBP |
| 44 | rs2388855 | 10p15 | *KLF6* | 3,56E-06 | **5,45E-03** | -4.52 | 3,80E-03 | LDL |
| 47 | rs4350264 | 10q21.3-q22.1 | *SLC25A16* | 1,01E-05 | 1,19E-02 | 4.31 | 3,98E-03 | LDL |
| 49 | rs11602954 | 11p15.5 | *BET1L* | 1,38E-07 | **2,99E-04** | -5.14 | 2,06E-04 | T1D |
| 53 | rs4514364 | 11p14-p13 | *LGR4* | 3,02E-06 | **4,49E-03** | 4.56 | 1,51E-03 | SBP |
| 53 | rs10767646 | 11p14.1 | *BDNF-AS* | 3,42E-06 | **4,49E-03** | 4.53 | 2,43E-03 | HDL |
| 55 | rs10160701 | 11p11.2 | *AMBRA1/HARBI1* | 5,47E-05 | 4,32E-02 | -3.94 | 9,82E-03 | HDL |
| 55 | rs2306029 | 11p11.2 | *LRP4-AS1/LRP4* | 1,31E-06 | **2,00E-03** | 4.72 | 7,96E-04 | SBP |
| 56 | rs7119750 | 11q13 | *RELA* | 1,08E-06 | **1,61E-03** | 4.76 | 6,39E-04 | SBP |
| 56 | rs7101916 | 11q13 | *RELA* | 5,54E-07 | **8,53E-04** | 4.89 | 3,29E-04 | SBP |
| 56 | rs12421691 | 11q13 | *KAT5* | 6,29E-07 | **1,05E-03** | 4.86 | 4,09E-04 | SBP |
| 58 | rs549932 | 11q14.2 | *TMEM135* | 1,12E-05 | 1,44E-02 | 4.29 | 6,04E-03 | T1D |
| 60 | rs3730071 | 12q12-q13 | *ADCY6/MIR4701* | 2,08E-06 | **3,02E-03** | 4.63 | 1,25E-03 | WHR |
| 61 | rs7311091 | 12q13 | *DDN* | 4,76E-06 | **6,62E-03** | -4.46 | 3,30E-03 | DBP |
| 64 | rs11614913 | 12q13.13 | *MIR196A2* | 1,20E-11 | **5,10E-07** | 6.62 | 1,70E-07 | WHR |
| 64 | rs3803042 | 12q13.13 | *MIR196A2* | 1,20E-11 | **5,10E-07** | 6.62 | 1,70E-07 | WHR |
| 64 | rs894737 | 12q13.13 | *HOXC6/HOXC4/HOXC5* | 2,08E-11 | **5,10E-07** | 6.54 | 1,70E-07 | WHR |
| 64 | rs754133 | 12q13.13 | *HOXC6/HOXC4/HOXC5* | 4,55E-11 | **5,10E-07** | 6.43 | 2,10E-07 | WHR |
| 66 | rs1047796 | 12q21.31 | *MLXIP* | 2,45E-05 | 2,55E-02 | 4.12 | 9,23E-03 | DBP |
| 71 | rs12438366 | 15q21-q22 | *SMAD3* | 1,03E-05 | 1,19E-02 | -4.3 | 7,68E-03 | DBP |
| 72 | rs7173826 | 15q22.33-q23 | *AAGAB* | 1,55E-05 | 1,75E-02 | -4.22 | 6,27E-03 | DBP |
| 79 | rs7350980 | 17q21.31 | *KANSL1* | 6,31E-08 | **1,72E-04** | 5.28 | 1,10E-04 | T1D |
| 79 | rs7221390 | 17q21.31 | *KANSL1* | 6,31E-08 | **1,72E-04** | 5.28 | 1,10E-04 | T1D |
| 79 | rs9303525 | 17q21.31 | *KANSL1* | 5,48E-08 | **1,42E-04** | 5.3 | 1,40E-04 | SBP |
| 79 | rs2696689 | 17q21.31 | *KANSL1-AS1* | 1,70E-07 | **3,53E-04** | 5.1 | 1,48E-04 | WHR |
| 80 | rs4794031 | 17q21.32 | *ZNF652* | 8,59E-06 | **9,79E-03** | 4.34 | 4,30E-03 | DBP |
| 80 | rs3179840 | 17q21.32 | *ZNF652* | 1,26E-05 | 1,44E-02 | 4.26 | 4,91E-03 | HDL |
| 80 | rs2906093 | 17q21.32 | *ZNF652* | 1,60E-05 | 1,75E-02 | 4.21 | 6,10E-03 | LDL |
| 81 | rs7226305 | 17q22 | *KIF2B* | 7,24E-07 | **1,30E-03** | -4.84 | 4,49E-04 | DBP |
| 81 | rs17730919 | 17q22 | *KIF2B* | 3,33E-06 | **4,49E-03** | 4.54 | 2,55E-03 | DBP |
| 82 | rs12601958 | 17q24.2 | *CEP112* | 5,42E-05 | 4,32E-02 | -3.94 | 8,74E-03 | TG |
| 83 | rs622924 | 18q21.33 | *PIGN* | 2,83E-06 | **4,49E-03** | -4.57 | 2,91E-03 | T2D |
| 83 | rs583339 | 18q21.33 | *PIGN* | 3,02E-06 | **4,49E-03** | -4.56 | 2,91E-03 | T2D |
| 87 | rs7227107 | 18q23 | *NFATC1* | 7,21E-06 | **9,79E-03** | -4.38 | 4,16E-03 | LDL |
| 89 | rs3760846 | 19q13.32 | *ERCC1* | 6,12E-06 | **8,05E-03** | 4.41 | 2,64E-03 | LDL |
| 90 | rs4813035 | 20p12 | *SLX4IP* | 2,35E-05 | 2,55E-02 | -4.13 | 7,60E-03 | DBP |
| 94 | rs17404303 | 20q13 | *BMP7* | 5,43E-06 | **6,62E-03** | 4.44 | 3,25E-03 | T1D |
| 95 | rs4817775 | 21 | *CBR3-AS1* | 8,17E-07 | **1,30E-03** | 4.81 | 5,12E-04 | SBP |
| Independent complex or single gene loci (LD-r2 < 0.2) with SNP(s) with a conditional FDR (condFDR) < 0.01 in bone mineral density (BMD, lumbar spine) given the association in other phenotypes. We defined the most significant BMD SNP in each LD block based on the minimum condFDR (min condFDR) for each phenotype. The second phenotype which provides the minimal FDR signal (driving phenotype) is listed. All loci with SNPs with condFDR < 0.01 were used to define the number of the loci. The following abbreviations were used: type 1 diabetes (T1D), type 2 diabetes (T2D), systolic blood pressure (SBP), diastolic blood pressure (DBP), high density lipoprotein (HDL), low density lipoprotein (LDL), triglycerides (TG), waist hip ratio (WHR), chromosome location (Map Loc.). Shaded r values represent nominally significant (p<0.05) Pearson correlations (age and BMI adjusted LS BMD vs Affymetrix signal values). Wald stats: z-score transformed from p values. | | | | | | | | |
