# Supplementary material for: Genetic Sharing with Cardiovascular Disease Risk Factors and Diabetes Reveals Novel Bone Mineral Density Loci
Source: PLoS One. 2015 Dec 22;10(12):e0144531. doi: 10.1371/journal.pone.0144531 (PMC4687843; doi:10.1371/journal.pone.0144531)
Supplement: S4 Table — (DOCX) [file pone.0144531.s012.docx]

| **S4 Table. Identified loci containing known SNPs or genes associated with lumbar spine BMD** | | | | | | | | |
| --- | --- | --- | --- | --- | --- | --- | --- | --- |
| **Locus#** | **SNP** | **Map loc.** | **Gene symbol** | **BMD**  **p-value** | **BMD FDR** | **Wald**  **stats** | **min cond FDR** | **Driving phenotype** |
| 3 | rs7521902* | 1p36.23-p35.1 | *WNT4** | 5,49E-07 | **8,53E-04** | 4.89 | 1,20E-03 | SBP |
| 3 | rs1014985 | 1p36.23-p35.1 | *WNT4** | 2,27E-07 | **4,89E-04** | -5.05 | 5,36E-04 | WHR |
| 3 | rs2982285 | 1p36.23-p35.1 | *WNT4** | 3,28E-06 | **4,49E-03** | -4.54 | 4,19E-03 | WHR |
| 3 | rs12030840 | 1p36.23-p35.1 | *WNT4** | 2,19E-09 | **7,83E-06** | 5.84 | 1,39E-05 | T2D |
| 3 | rs932371 | 1p36.23-p35.1 | *WNT4** | 1,92E-09 | **7,83E-06** | 5.86 | 1,39E-05 | T2D |
| 3 | rs2473236 | 1p36.23-p35.1 | *WNT4** | 4,89E-06 | **6,62E-03** | -4.46 | 3,97E-03 | WHR |
| 4 | rs4598465 | 1p36 | *ZBTB40** | 1,02E-06 | **1,61E-03** | 4.77 | 7,37E-04 | WHR |
| 4 | rs10917209 | 1p36 | *ZBTB40** | 1,39E-06 | **2,00E-03** | 4.71 | 1,37E-03 | LDL |
| 4 | rs10917214 | 1p36 | *ZBTB40** | 2,79E-07 | **4,89E-04** | -5.01 | 5,35E-04 | LDL |
| 4 | rs4655048 | 1p36 | *ZBTB40** | 6,94E-08 | **1,72E-04** | 5.26 | 2,49E-04 | HDL |
| 4 | rs1316342 | 1p36 | *ZBTB40** | 1,28E-06 | **2,00E-03** | 4.73 | 1,81E-03 | LDL |
| 4 | rs10753536 | 1p36 | *ZBTB40** | 1,93E-07 | **4,15E-04** | -5.08 | 2,13E-04 | WHR |
| 4 | rs12029258 | 1p36 | *ZBTB40** | 3,46E-07 | **5,82E-04** | 4.97 | 3,28E-04 | SBP |
| 4 | rs11810369 | 1p36 | *ZBTB40** | 2,08E-13 | **5,10E-07** | -7.17 | 2,50E-07 | HDL |
| 5 | rs12723796 | 1p36 | *ZBTB40** | 3,77E-17 | **5,10E-07** | -8.22 | 3,58E-07 | T2D |
| 5 | rs12568930 | 1p36 | *ZBTB40** | 1,26E-21 | **5,10E-07** | -9.32 | 3,55E-07 | TG |
| 5 | rs10493013 | 1p36 | *ZBTB40** | 9,65E-22 | **5,10E-07** | -9.35 | 3,56E-07 | LDL |
| 5 | rs7543680 | 1p36 | *ZBTB40** | 1,72E-11 | **5,10E-07** | -6.57 | 1,44E-07 | HDL |
| 5 | rs4394609 | 1p36 | *ZBTB40** | 6,32E-14 | **5,10E-07** | -7.32 | 3,29E-07 | TG |
| 5 | rs10799749 | 1p36 | *ZBTB40** | 1,06E-07 | **2,51E-04** | 5.19 | 1,80E-04 | SBP |
| 6 | rs4655059 | 1p36 | *ZBTB40** | 9,97E-08 | **2,51E-04** | 5.2 | 1,69E-04 | SBP |
| 9 | rs1367447 | 1p31.3 | *WLS/GNG12-AS1* | 7,60E-15 | **5,10E-07** | -7.59 | 3,30E-07 | SBP |
| 9 | rs1430742 | 1p31.3 | *WLS/GNG12-AS1* | 3,29E-19 | **5,10E-07** | -8.74 | 3,30E-07 | SBP |
| 9 | rs2566755 | 1p31.3 | *WLS/GNG12-AS1* | 3,02E-19 | **5,10E-07** | -8.75 | 3,30E-07 | SBP |
| 9 | rs2195682 | 1p31.3 | *WLS/GNG12-AS1* | 1,21E-20 | **5,10E-07** | 9.09 | 3,18E-07 | TG |
| 9 | rs4233320 | 1p31.3 | *WLS/GNG12-AS1* | 4,45E-20 | **5,10E-07** | 8.96 | 3,18E-07 | TG |
| 9 | rs891528 | 1p31.3 | *WLS/GNG12-AS1* | 4,88E-13 | **5,10E-07** | -7.05 | 3,66E-07 | DBP |
| 9 | rs944082 | 1p31.3 | *WLS/GNG12-AS1* | 3,22E-16 | **5,10E-07** | -7.97 | 3,98E-07 | T2D |
| 9 | rs12407028* | 1p31.3 | *WLS/GNG12-AS1* | 8,39E-21 | **5,10E-07** | 9.13 | 3,38E-07 | TG |
| 9 | rs12568456 | 1p31.3 | *WLS/MIR1262/GNG12-AS1* | 6,67E-17 | **5,10E-07** | -8.15 | 3,29E-07 | DBP |
| 15 | rs6734097 | 2p21 | *SPTBN1** | 2,74E-12 | **5,10E-07** | 6.82 | 3,64E-07 | TG |
| 15 | rs7607093 | 2p21 | *SPTBN1** | 3,21E-12 | **5,10E-07** | 6.8 | 4,36E-07 | TG |
| 15 | rs6752877 | 2p21 | *SPTBN1** | 2,00E-12 | **5,10E-07** | 6.87 | 3,97E-07 | TG |
| 15 | rs11898505 | 2p21 | *SPTBN1** | 4,06E-12 | **5,10E-07** | 6.77 | 4,36E-07 | TG |
| 17 | rs1878526* | 2q14.1 | *INSIG2** | 4,33E-06 | **5,45E-03** | -4.48 | 3,15E-03 | LDL |
| 17 | rs4594452 | 2q14.1 | *INSIG2** | 4,80E-06 | **6,62E-03** | -4.46 | 6,13E-03 | TG |
| 17 | rs12621455 | 2q14.1 | *INSIG2** | 5,43E-06 | **6,62E-03** | -4.44 | 4,93E-03 | T2D |
| 17 | rs7587150 | 2q14.1 | *INSIG2** | 6,56E-06 | **8,05E-03** | -4.4 | 6,30E-03 | T2D |
| 17 | rs4073566 | 2q14.1 | *INSIG2** | 5,10E-06 | **6,62E-03** | 4.45 | 5,74E-03 | TG |
| 18 | rs6710388 | 2q24-q31 | *GALNT3** | 8,01E-09 | **2,80E-05** | 5.63 | 2,57E-05 | SBP |
| 18 | rs1346004* | 2q24-q31 | *GALNT3** | 5,61E-09 | **1,84E-05** | 5.69 | 1,85E-05 | SBP |
| 20 | rs416486 | 3p21 | *CTNNB1** | 2,29E-11 | **5,10E-07** | -6.53 | 3,26E-07 | LDL |
| 20 | rs11717807 | 3p21 | *CTNNB1** | 2,29E-11 | **5,10E-07** | -6.53 | 3,26E-07 | LDL |
| 20 | rs422623 | 3p21 | *CTNNB1** | 1,95E-11 | **5,10E-07** | -6.55 | 3,26E-07 | LDL |
| 20 | rs385905 | 3p21 | *CTNNB1** | 2,49E-11 | **5,10E-07** | -6.51 | 2,74E-07 | LDL |
| 20 | rs428510 | 3p21 | *CTNNB1** | 2,29E-11 | **5,10E-07** | -6.53 | 3,26E-07 | LDL |
| 20 | rs368006 | 3p21 | *CTNNB1** | 1,78E-11 | **5,10E-07** | 6.56 | 2,30E-07 | LDL |
| 23 | rs7621699 | 3q25.31 | *LEKR1** | 9,23E-06 | **1,19E-02** | 4.33 | 3,83E-03 | WHR |
| 23 | rs16826948 | 3q25.31 | *LEKR1** | 1,11E-05 | **1,19E-02** | 4.29 | 5,65E-03 | WHR |
| 26 | rs3755955* | 4p16.3 | *IDUA** | 6,62E-08 | **1,72E-04** | 5.27 | 1,79E-04 | WHR |
| 29 | rs2904180 | 4q21.1 | *MEPE** | 3,87E-09 | **1,48E-05** | 5.75 | 1,81E-05 | SBP |
| 29 | rs13117929 | 4q21.1 | *MEPE** | 4,39E-09 | **1,48E-05** | 5.73 | 1,81E-05 | SBP |
| 29 | rs6532023* | 4q21.1 | *MEPE** | 5,44E-12 | **5,10E-07** | 6.73 | 2,10E-07 | LDL |
| 29 | rs1471399 | 4q21.1 | *MEPE** | 5,44E-12 | **5,10E-07** | 6.73 | 1,73E-07 | LDL |
| 29 | rs1471403 | 4q21.1 | *MEPE** | 5,44E-12 | **5,10E-07** | 6.73 | 2,10E-07 | LDL |
| 32 | rs11755164* | 6p21.1-p12.3 | *SUPT3H** | 1,80E-07 | **4,15E-04** | -5.09 | 4,15E-04 | DBP |
| 32 | rs12526711 | 6p21.1-p12.3 | *SUPT3H** | 6,39E-07 | **1,05E-03** | 4.86 | 4,76E-04 | WHR |
| 32 | rs3799986 | 6p21.1-p12.3 | *SUPT3H** | 1,47E-06 | **2,47E-03** | -4.7 | 6,39E-04 | WHR |
| 33 | rs9479055 | 6q25.1 | *CCDC170** | 5,31E-12 | **5,10E-07** | -6.73 | 1,04E-07 | LDL |
| 33 | rs11753987 | 6q25.1 | *CCDC170** | 1,02E-11 | **5,10E-07** | -6.64 | 1,04E-07 | LDL |
| 33 | rs10872673 | 6q25.1 | *CCDC170** | 2,82E-13 | **5,10E-07** | -7.13 | 1,04E-07 | LDL |
| 33 | rs1856859 | 6q25.1 | *CCDC170** | 7,96E-16 | **5,10E-07** | 7.86 | 9,71E-08 | LDL |
| 33 | rs9371537 | 6q25.1 | *CCDC170** | 1,80E-15 | **5,10E-07** | 7.76 | 9,54E-08 | LDL |
| 33 | rs7761420 | 6q25.1 | *CCDC170** | 1,00E-16 | **5,10E-07** | -8.1 | 1,04E-07 | LDL |
| 33 | rs9397425 | 6q25.1 | *CCDC170** | 1,91E-15 | **5,10E-07** | 7.76 | 9,47E-08 | LDL |
| 33 | rs11759804 | 6q25.1 | *CCDC170** | 1,80E-15 | **5,10E-07** | 7.76 | 9,62E-08 | LDL |
| 33 | rs7753676 | 6q25.1 | *CCDC170** | 1,22E-16 | **5,10E-07** | -8.08 | 9,71E-08 | LDL |
| 33 | rs9479072 | 6q25.1 | *CCDC170** | 1,78E-15 | **5,10E-07** | -7.76 | 1,04E-07 | LDL |
| 33 | rs1871859 | 6q25.1 | *CCDC170** | 1,64E-18 | **5,10E-07** | 8.57 | 9,47E-08 | LDL |
| 33 | rs9478223 | 6q25.1 | *CCDC170** | 4,26E-07 | **7,00E-04** | 4.94 | 5,45E-04 | T2D |
| 33 | rs6925996 | 6q25.1 | *CCDC170** | 7,38E-20 | **5,10E-07** | 8.9 | 9,54E-08 | LDL |
| 33 | rs9383930 | 6q25.1 | *CCDC170** | 3,39E-10 | **1,51E-06** | 6.13 | 1,55E-06 | SBP |
| 33 | rs9397065 | 6q25.1 | *CCDC170** | 4,72E-10 | **2,30E-06** | 6.08 | 1,85E-06 | SBP |
| 33 | rs1340874 | 6q25.1 | *CCDC170** | 7,39E-10 | **3,45E-06** | 6.01 | 3,18E-06 | HDL |
| 33 | rs11155800 | 6q25.1 | *CCDC170** | 1,10E-11 | **5,10E-07** | 6.63 | 3,00E-07 | TG |
| 33 | rs6904261 | 6q25.1 | *CCDC170** | 2,54E-15 | **5,10E-07** | 7.72 | 3,18E-07 | TG |
| 33 | rs6932603 | 6q25.1 | *CCDC170** | 1,59E-18 | **5,10E-07** | 8.57 | 3,73E-07 | T2D |
| 33 | rs10872676 | 6q25.1 | *CCDC170** | 1,04E-17 | **5,10E-07** | 8.36 | 1,32E-07 | HDL |
| 33 | rs7751941* | 6q25.1 | *CCDC170** | 5,27E-16 | **5,10E-07** | 7.91 | 3,30E-07 | SBP |
| 33 | rs7752591 | 6q25.1 | *CCDC170** | 1,75E-17 | **5,10E-07** | 8.3 | 3,38E-07 | T2D |
| 33 | rs6913578 | 6q25.1 | *CCDC170** | 1,38E-16 | **5,10E-07** | 8.07 | 3,37E-07 | DBP |
| 33 | rs865898 | 6q25.1 | *CCDC170** | 2,76E-13 | **5,10E-07** | 7.13 | 3,11E-07 | TG |
| 33 | rs712219 | 6q24-q27 | *ESR1* | 2,19E-12 | **5,10E-07** | 6.85 | 3,11E-07 | T1D |
| 33 | rs851970 | 6q24-q27 | *ESR1* | 4,63E-12 | **5,10E-07** | 6.75 | 3,37E-07 | T2D |
| 33 | rs6557164 | 6q24-q27 | *ESR1* | 3,97E-09 | **1,48E-05** | 5.74 | 1,26E-05 | SBP |
| 33 | rs3020333 | 6q24-q27 | *ESR1* | 1,59E-14 | **5,10E-07** | -7.5 | 1,40E-07 | HDL |
| 33 | rs3020334 | 6q24-q27 | *ESR1* | 5,51E-15 | **5,10E-07** | -7.63 | 1,32E-07 | HDL |
| 33 | rs851982 | 6q24-q27 | *ESR1* | 1,02E-11 | **5,10E-07** | -6.64 | 1,53E-07 | HDL |
| 33 | rs851980 | 6q24-q27 | *ESR1* | 1,41E-07 | **2,99E-04** | -5.14 | 3,44E-04 | HDL |
| 33 | rs2982554 | 6q24-q27 | *ESR1* | 7,84E-14 | **5,10E-07** | -7.29 | 1,58E-07 | HDL |
| 33 | rs3020349 | 6q24-q27 | *ESR1* | 6,60E-14 | **5,10E-07** | -7.32 | 1,34E-07 | HDL |
| 33 | rs2152750 | 6q24-q27 | *ESR1* | 1,25E-14 | **5,10E-07** | -7.53 | 1,58E-07 | HDL |
| 33 | rs1124674 | 6q24-q27 | *ESR1* | 1,03E-11 | **5,10E-07** | -6.64 | 1,64E-07 | HDL |
| 33 | rs2504070 | 6q24-q27 | *ESR1* | 1,55E-09 | **6,35E-06** | -5.89 | 6,09E-06 | SBP |
| 33 | rs1890010 | 6q24-q27 | *ESR1* | 1,99E-11 | **5,10E-07** | -6.55 | 2,83E-07 | HDL |
| 33 | rs2504069 | 6q24-q27 | *ESR1* | 1,99E-11 | **5,10E-07** | -6.55 | 2,50E-07 | HDL |
| 33 | rs2504063 | 6q24-q27 | *ESR1* | 3,36E-12 | **5,10E-07** | -6.79 | 1,98E-07 | HDL |
| 34 | rs10226308* | 7p15.2 | *NME8** | 1,45E-06 | **2,47E-03** | -4.7 | 1,69E-03 | LDL |
| 34 | rs10256195 | 7p15.2 | *NME8** | 1,45E-06 | **2,47E-03** | -4.7 | 1,29E-03 | LDL |
| 34 | rs10276139 | 7p15.2 | *NME8** | 1,45E-06 | **2,47E-03** | -4.7 | 1,69E-03 | LDL |
| 34 | rs17236800 | 7p14.1 | *SFRP4* | 1,45E-06 | **2,47E-03** | -4.7 | 1,29E-03 | LDL |
| 34 | rs10264106 | 7p14.1 | *SFRP4* | 1,67E-06 | **2,47E-03** | -4.67 | 1,13E-03 | LDL |
| 35 | rs2722281 | 7p14.1 | *EPDR1* | 1,44E-06 | **2,47E-03** | 4.7 | 8,75E-04 | SBP |
| 35 | rs2722286 | 7p14.1 | *EPDR1* | 1,50E-06 | **2,47E-03** | 4.7 | 9,55E-04 | SBP |
| 35 | rs1717739 | 7p14.1 | *EPDR1* | 8,19E-09 | **2,80E-05** | -5.63 | 4,58E-05 | LDL |
| 35 | rs1721396 | 7p14.1 | *EPDR1* | 1,57E-08 | **5,18E-05** | -5.52 | 6,94E-05 | DBP |
| 35 | rs1357651 | 7p14.1 | *EPDR1* | 1,81E-16 | **5,10E-07** | -8.04 | 3,58E-07 | T2D |
| 35 | rs1721385 | 7p14.1 | *EPDR1* | 9,67E-09 | **3,45E-05** | -5.6 | 4,62E-05 | LDL |
| 35 | rs1717731 | 7p14.1 | *EPDR1* | 9,67E-09 | **3,45E-05** | -5.6 | 4,62E-05 | LDL |
| 35 | rs1403987 | 7p14-p13 | *STARD3NL** | 2,19E-16 | **5,10E-07** | -8.01 | 3,76E-07 | T2D |
| 35 | rs6959212* | 7p14-p13 | *STARD3NL** | 1,18E-17 | **5,10E-07** | -8.35 | 3,00E-07 | TG |
| 35 | rs1524058 | 7p14-p13 | *STARD3NL** | 4,26E-17 | **5,10E-07** | -8.2 | 3,30E-07 | SBP |
| 37 | rs6952113 | 7q31.31 | *CPED1* | 1,56E-06 | **2,47E-03** | 4.69 | 1,57E-03 | SBP |
| 37 | rs13245690* | 7q31.31 | *CPED1* | 4,08E-07 | **7,00E-04** | 4.94 | 4,97E-04 | T2D |
| 39 | rs3779381 | 7q31 | *WNT16** | 8,74E-14 | **5,10E-07** | -7.28 | 2,74E-07 | LDL |
| 39 | rs718766 | 7q22.1-q31.1 | *FAM3C* | 5,29E-17 | **5,10E-07** | -8.18 | 2,51E-07 | LDL |
| 39 | rs7776725 | 7q22.1-q31.1 | *FAM3C* | 6,30E-17 | **5,10E-07** | -8.16 | 1,91E-07 | LDL |
| 40 | rs3134036 | 8q24 | *SAMD12-AS1* | 7,45E-08 | **2,09E-04** | -5.25 | 2,18E-04 | DBP |
| 40 | rs3133582 | 8q24 | *SAMD12-AS1* | 7,39E-07 | **1,30E-03** | -4.83 | 4,09E-04 | WHR |
| 40 | rs3133585 | 8q24 | *TNFRSF11B** | 2,46E-11 | **5,10E-07** | -6.52 | 2,36E-07 | T1D |
| 40 | rs3134086 | 8q24 | *TNFRSF11B** | 7,48E-07 | **1,30E-03** | -4.83 | 5,57E-04 | WHR |
| 40 | rs16891598 | 8q24 | *TNFRSF11B** | 1,22E-08 | **4,23E-05** | 5.56 | 4,83E-05 | SBP |
| 40 | rs6651219 | 8q24 | *TNFRSF11B** | 1,43E-08 | **5,18E-05** | 5.53 | 5,96E-05 | SBP |
| 40 | rs4876868 | 8q24 | *TNFRSF11B** | 6,26E-06 | **8,05E-03** | -4.41 | 9,01E-03 | T2D |
| 40 | rs11573885 | 8q24 | *TNFRSF11B** | 1,26E-12 | **5,10E-07** | -6.93 | 3,00E-07 | TG |
| 40 | rs1032129 | 8q24 | *TNFRSF11B** | 3,05E-06 | **4,49E-03** | 4.56 | 1,87E-03 | SBP |
| 40 | rs11573829 | 8q24 | *TNFRSF11B** | 5,24E-20 | **5,10E-07** | 8.94 | 3,35E-07 | T2D |
| 40 | rs10505346 | 8q24 | *TNFRSF11B** | 6,95E-09 | **2,27E-05** | 5.65 | 3,33E-05 | SBP |
| 40 | rs3102735 | 8q24 | *TNFRSF11B** | 7,09E-06 | **9,79E-03** | 4.38 | 7,38E-03 | SBP |
| 40 | rs1385499 | 8q24 | *TNFRSF11B** | 1,73E-16 | **5,10E-07** | 8.04 | 3,46E-07 | T2D |
| 40 | rs1564860 | 8q24 | *TNFRSF11B** | 1,90E-14 | **5,10E-07** | 7.47 | 2,98E-07 | LDL |
| 40 | rs7839059 | 8q24 | *TNFRSF11B** | 2,59E-17 | **5,10E-07** | 8.26 | 3,73E-07 | T2D |
| 40 | rs1825511 | 8q24 | *TNFRSF11B** | 1,09E-09 | **4,22E-06** | 5.95 | 7,73E-06 | TG |
| 40 | rs6469804 | 8q23-q24.1 | *COLEC10* | 7,67E-19 | **5,10E-07** | 8.65 | 2,36E-07 | T1D |
| 40 | rs10955924 | 8q23-q24.1 | *COLEC10* | 9,61E-20 | **5,10E-07** | 8.87 | 2,36E-07 | T1D |
| 45 | rs4568902 | 10p12.1 | *MPP7** | 1,26E-09 | **5,17E-06** | -5.93 | 1,22E-05 | TG |
| 45 | rs3905706* | 10p12.1 | *MPP7** | 1,19E-09 | **5,17E-06** | -5.94 | 8,03E-06 | WHR |
| 45 | rs7088552 | 10p12.1 | *MPP7** | 1,63E-09 | **6,35E-06** | -5.89 | 1,08E-05 | T1D |
| 46 | rs1373004* | 10q11.2 | *MBL2** | 2,52E-08 | **7,77E-05** | -5.44 | 1,13E-04 | SBP |
| 48 | rs7071206* | 10q22 | *KCNMA1** | 4,33E-13 | **5,10E-07** | -7.07 | 3,38E-07 | T2D |
| 48 | rs1877998 | 10q22 | *KCNMA1** | 1,16E-11 | **5,10E-07** | -6.62 | 3,06E-07 | TG |
| 48 | rs10509391 | 10q22 | *KCNMA1** | 1,60E-11 | **5,10E-07** | -6.58 | 3,00E-07 | TG |
| 50 | rs9787942 | 11p15.3 | *SOX6** | 1,03E-05 | 1,19E-02 | 4.31 | 8,08E-03 | T2D |
| 51 | rs11023718 | 11p15.3 | *SOX6** | 1,11E-05 | 1,19E-02 | -4.29 | 4,40E-03 | SBP |
| 52 | rs10219384 | 11p15.3 | *SOX6** | 7,42E-06 | **9,79E-03** | 4.37 | 3,57E-03 | DBP |
| 52 | rs297366 | 11p15.3 | *SOX6** | 7,85E-06 | **9,79E-03** | 4.36 | 3,57E-03 | DBP |
| 52 | rs297365 | 11p15.3 | *SOX6** | 7,85E-06 | **9,79E-03** | 4.36 | 3,57E-03 | DBP |
| 52 | rs2351964 | 11p15.3 | *SOX6** | 7,85E-06 | **9,79E-03** | 4.36 | 3,57E-03 | DBP |
| 54 | rs2021807 | 11p14.1 | *DCDC5** | 5,30E-08 | **1,42E-04** | -5.31 | 1,64E-04 | WHR |
| 54 | rs911268 | 11p14.1 | *DCDC5** | 5,30E-08 | **1,42E-04** | -5.31 | 1,60E-04 | WHR |
| 54 | rs507969 | 11p14.1 | *DCDC5** | 4,78E-08 | **1,42E-04** | 5.33 | 1,70E-04 | WHR |
| 54 | rs163883 | 11p14.1 | *DCDC5** | 5,42E-08 | **1,42E-04** | 5.31 | 1,92E-04 | WHR |
| 54 | rs163879* | 11p14.1 | *DCDC5** | 4,22E-08 | **1,16E-04** | 5.35 | 2,11E-04 | TG |
| 57 | rs3736228* | 11q13.4 | *LRP5** | 5,10E-12 | **5,10E-07** | 6.74 | 2,74E-07 | LDL |
| 57 | rs11228262 | 11q13 | *PPP6R3* | 3,83E-11 | **5,10E-07** | 6.45 | 3,82E-07 | SBP |
| 57 | rs11228292 | 11q13 | *PPP6R3* | 1,20E-10 | **6,36E-07** | 6.28 | 6,89E-07 | SBP |
| 57 | rs4930238 | 11q13.2 | *GAL* | 3,76E-07 | **7,00E-04** | -4.96 | 5,75E-04 | SBP |
| 57 | rs7935394 | 11q13.2 | *GAL* | 1,06E-06 | **1,61E-03** | -4.76 | 1,15E-03 | WHR |
| 57 | rs2510387 | 11q13.2 | *GAL* | 1,23E-05 | 1,44E-02 | -4.27 | 5,65E-03 | WHR |
| 59 | rs4283041 | 12p13 | *LINC00942* | 1,13E-07 | **2,99E-04** | -5.18 | 3,12E-04 | DBP |
| 59 | rs2887571* | 12p13 | *LINC00942* | 1,10E-07 | **2,51E-04** | -5.18 | 3,15E-04 | DBP |
| 62 | rs1054442 | 12q13 | *DDN* | 2,79E-07 | **4,89E-04** | -5.01 | 6,67E-04 | TG |
| 62 | rs11168850 | 12q13.12 | *RHEBL1* | 1,23E-06 | **2,00E-03** | -4.73 | 2,24E-03 | T2D |
| 62 | rs6580699 | 12q13.1 | *DHH** | 1,39E-06 | **2,00E-03** | -4.71 | 1,84E-03 | T1D |
| 63 | rs10747666 | 12q13 | *AAAS* | 2,76E-11 | **5,10E-07** | 6.5 | 2,83E-07 | HDL |
| 63 | rs2016266* | 12q13.13 | *SP7** | 4,81E-12 | **5,10E-07** | 6.74 | 2,22E-07 | HDL |
| 65 | rs10778517 | 12q23.3 | *TMEM263(C12orf23)** | 2,08E-06 | **3,02E-03** | -4.63 | 2,46E-03 | T2D |
| 65 | rs1053051* | 12q23.3 | *TMEM263(C12orf23)** | 1,39E-06 | **2,00E-03** | -4.71 | 3,47E-03 | LDL |
| 67 | rs9590679 | 13q14.11 | *DGKH* | 8,28E-06 | **9,79E-03** | 4.35 | 5,49E-03 | DBP |
| 67 | rs12861586 | 13q | *AKAP11** | 1,14E-06 | **2,00E-03** | -4.75 | 8,98E-04 | DBP |
| 67 | rs7995240 | 13q | *AKAP11** | 2,33E-12 | **5,10E-07** | -6.84 | 1,53E-07 | HDL |
| 67 | rs7998154 | 13q | *AKAP11** | 2,05E-07 | **4,15E-04** | 5.07 | 4,82E-04 | LDL |
| 67 | rs238270 | 13q | *AKAP11** | 5,23E-11 | **5,10E-07** | -6.41 | 2,23E-07 | HDL |
| 67 | rs7338012 | 13q | *AKAP11** | 9,17E-08 | **2,51E-04** | 5.21 | 3,64E-04 | DBP |
| 67 | rs7992970 | 13q | *AKAP11** | 1,02E-12 | **5,10E-07** | -6.96 | 3,98E-07 | T2D |
| 67 | rs7988075 | 13q | *AKAP11** | 8,83E-13 | **5,10E-07** | -6.98 | 3,98E-07 | T2D |
| 67 | rs1359201 | 13q | *AKAP11** | 1,31E-11 | **5,10E-07** | -6.6 | 2,30E-07 | LDL |
| 67 | rs1475249 | 13q | *AKAP11** | 1,59E-13 | **5,10E-07** | -7.2 | 3,76E-07 | T2D |
| 67 | rs11840862 | 13q | *AKAP11** | 1,35E-24 | **5,10E-07** | 9.99 | 4,32E-07 | LDL |
| 67 | rs17638544 | 13q | *AKAP11** | 1,09E-13 | **5,10E-07** | -7.25 | 3,37E-07 | DBP |
| 67 | rs7317323 | 13q | *AKAP11** | 1,40E-11 | **5,10E-07** | -6.6 | 3,37E-07 | DBP |
| 67 | rs9533093 | 13q | *AKAP11** | 5,27E-12 | **5,10E-07** | -6.73 | 2,30E-07 | LDL |
| 67 | rs17457561 | 13q | *AKAP11** | 9,09E-17 | **5,10E-07** | 8.12 | 2,36E-07 | T1D |
| 67 | rs7987211 | 13q | *AKAP11** | 1,29E-13 | **5,10E-07** | -7.23 | 2,51E-07 | LDL |
| 67 | rs7992415 | 13q | *AKAP11** | 1,29E-13 | **5,10E-07** | -7.23 | 3,29E-07 | TG |
| 67 | rs7994531 | 13q | *AKAP11** | 5,76E-12 | **5,10E-07** | -6.72 | 2,10E-07 | LDL |
| 67 | rs7326472 | 13q | *AKAP11** | 2,78E-15 | **5,10E-07** | -7.71 | 3,06E-07 | TG |
| 67 | rs9566973 | 13q | *AKAP11** | 1,15E-11 | **5,10E-07** | -6.62 | 2,10E-07 | LDL |
| 67 | rs9533099 | 13q | *AKAP11** | 2,58E-11 | **5,10E-07** | -6.51 | 1,91E-07 | LDL |
| 67 | rs9594759 | 13q14 | *TNFSF11* | 9,84E-16 | **5,10E-07** | 7.84 | 2,51E-07 | LDL |
| 67 | rs2062305 | 13q14 | *TNFSF11* | 1,22E-16 | **5,10E-07** | 8.08 | 3,21E-07 | HDL |
| 67 | rs6561055 | 13q14 | *TNFSF11* | 1,03E-12 | **5,10E-07** | -6.95 | 3,00E-07 | TG |
| 67 | rs9533128 | 13q14 | *TNFSF11* | 6,73E-10 | **2,82E-06** | 6.02 | 3,17E-06 | T1D |
| 70 | rs1286083* | 14q31-q32.1 | *RPS6KA5** | 5,32E-12 | **5,10E-07** | -6.73 | 4,25E-07 | T2D |
| 70 | rs1286079 | 14q31-q32.1 | *RPS6KA5** | 6,07E-12 | **5,10E-07** | -6.71 | 4,25E-07 | T2D |
| 70 | rs1286077 | 14q31-q32.1 | *RPS6KA5** | 6,47E-12 | **5,10E-07** | -6.7 | 4,55E-07 | T1D |
| 70 | rs1286153 | 14q31-q32.1 | *RPS6KA5** | 5,68E-12 | **5,10E-07** | -6.72 | 4,25E-07 | T2D |
| 70 | rs1286150 | 14q31-q32.1 | *RPS6KA5** | 5,68E-12 | **5,10E-07** | -6.72 | 5,76E-07 | TG |
| 70 | rs1286147 | 14q31-q32.1 | *RPS6KA5** | 5,32E-12 | **5,10E-07** | -6.73 | 4,25E-07 | T2D |
| 73 | rs9921222* | 16p13.3 | *AXIN1/LUC7L* | 9,85E-09 | **3,45E-05** | 5.6 | 4,66E-05 | WHR |
| 74 | rs4985155* | 16p13.11 | *PDXDC1* | 4,66E-07 | **8,53E-04** | -4.92 | 5,70E-04 | LDL |
| 74 | rs2740 | 16p13.11 | *PDXDC1/NTAN1* | 1,31E-06 | **2,00E-03** | -4.72 | 1,75E-03 | TG |
| 74 | rs16966952 | 16p13.11 | *PDXDC1/NTAN1* | 5,84E-07 | **1,05E-03** | -4.88 | 7,00E-04 | WHR |
| 75 | rs1564981* | 16q12-q13 | *CYLD** | 6,95E-09 | **2,27E-05** | 5.65 | 2,49E-05 | T1D |
| 75 | rs1872678 | 16q12-q13 | *CYLD** | 6,95E-09 | **2,27E-05** | 5.65 | 2,49E-05 | T1D |
| 76 | rs299946 | 16q24 | *FOXL1** | 4,25E-06 | **5,45E-03** | -4.49 | 3,68E-03 | WHR |
| 76 | rs10048146* | 16q24 | *FOXL1** | 1,34E-08 | **4,23E-05** | 5.54 | 5,78E-05 | HDL |
| 77 | rs1983490 | 17q21.31 | *MEOX1* | 2,33E-08 | **7,77E-05** | -5.45 | 1,02E-04 | SBP |
| 77 | rs1828720 | 17q12-q21 | *SOST** | 5,67E-09 | **2,27E-05** | -5.69 | 2,60E-05 | TG |
| 77 | rs4792909* | 17q12-q21 | *SOST** | 3,76E-09 | **1,48E-05** | -5.75 | 2,29E-05 | SBP |
| 78 | rs227584* | 17q21.31 | *C17orf53** | 2,48E-07 | **4,89E-04** | -5.04 | 2,04E-04 | SBP |
| 78 | rs7207464 | 17q21.31 | *ASB16* | 1,75E-07 | **3,53E-04** | -5.1 | 1,81E-04 | SBP |
| 84 | rs2980980 | 18q21.33 | *KIAA1468* | 6,49E-07 | **1,05E-03** | -4.86 | 1,54E-03 | TG |
| 84 | rs17720953 | 18q22.1 | *TNFRSF11A** | 2,56E-05 | 2,55E-02 | 4.11 | 8,38E-03 | WHR |
| 84 | rs8083511 | 18q22.1 | *TNFRSF11A** | 3,83E-07 | **7,00E-04** | -4.96 | 6,35E-04 | T2D |
| 85 | rs8089829 | 18q22.1 | *TNFRSF11A** | 4,23E-06 | **5,45E-03** | -4.49 | 3,21E-03 | WHR |
| 86 | rs884205* | 18q22.1 | *TNFRSF11A** | 2,02E-09 | **7,83E-06** | -5.85 | 1,50E-05 | T2D |
| 86 | rs2957128 | 18q22.1 | *TNFRSF11A** | 1,13E-08 | **4,23E-05** | -5.57 | 6,44E-05 | TG |
| 88 | rs13343954 | 19q13.12 | *RHPN2* | 7,64E-06 | **9,79E-03** | -4.37 | 7,88E-03 | TG |
| 88 | rs9304844 | 19q13.12 | *GPATCH1** | 4,48E-09 | **1,84E-05** | -5.72 | 1,50E-05 | LDL |
| 88 | rs7247748 | 19q13.12 | *GPATCH1** | 4,48E-09 | **1,84E-05** | -5.72 | 1,50E-05 | LDL |
| 88 | rs3760893 | 19q13.12 | *GPATCH1** | 4,48E-09 | **1,84E-05** | -5.72 | 1,50E-05 | LDL |
| 88 | rs10416218* | 19q13.12 | *GPATCH1** | 3,94E-09 | **1,48E-05** | -5.75 | 1,50E-05 | LDL |
| 88 | rs2287679 | 19q13.12 | *GPATCH1** | 4,48E-09 | **1,84E-05** | -5.72 | 1,50E-05 | LDL |
| 91 | rs10485741 | 20p12.1-p11.23 | *JAG1** | 6,40E-06 | **8,05E-03** | 4.4 | 4,00E-03 | T1D |
| 91 | rs3790159 | 20p12.1-p11.23 | *JAG1** | 1,79E-11 | **5,10E-07** | 6.56 | 4,91E-07 | T2D |
| 91 | rs3790160* | 20p12.1-p11.23 | *JAG1** | 1,79E-11 | **5,10E-07** | 6.56 | 4,91E-07 | T2D |
| 91 | rs17457340 | 20p12.1-p11.23 | *JAG1** | 1,43E-06 | **2,47E-03** | 4.7 | 1,62E-03 | DBP |
| 91 | rs6040061 | 20p12.1-p11.23 | *JAG1** | 1,80E-11 | **5,10E-07** | 6.56 | 3,31E-07 | T1D |
| 92 | rs1108850 | 20p12.1-p11.23 | *JAG1** | 3,10E-06 | **4,49E-03** | 4.55 | 1,95E-03 | WHR |
| 92 | rs6040286 | 20p12.1-p11.23 | *JAG1** | 6,39E-06 | **8,05E-03** | -4.4 | 2,95E-03 | DBP |
| 92 | rs6104690 | 20p12.1-p11.23 | *JAG1** | 5,70E-06 | **8,05E-03** | -4.43 | 2,95E-03 | DBP |
| 93 | rs6040357 | 20p12.1-p11.23 | *JAG1** | 3,24E-06 | **4,49E-03** | 4.54 | 3,61E-03 | TG |
| Independent complex or single gene loci (LD-r2 < 0.2) with SNP(s) with a conditional FDR (condFDR) < 0.01 in bone mineral density (BMD, femoral neck) given the association in other phenotypes. We defined the most significant BMD associated SNPs in each LD block based on the minimum condFDR (min condFDR) for each phenotype. The second phenotype which provides the minimal FDR signal (Driving phenotype) is listed. All loci with SNPs with condFDR < 0.01 were used to define the number of the loci. The following abbreviations were used: type 1 diabetes (T1D), type 2 diabetes (T2D), systolic blood pressure (SBP), diastolic blood pressure (DBP), high density lipoprotein (HDL), low density lipoprotein (LDL), triglycerides (TG), waist hip ratio (WHR), chromosome location (Map Loc.). Shaded r values represent nominally significant (p<0.05) Pearson correlations (age and BMI adjusted LS BMD vs Affymetrix signal values). NA: not applicable (undetected). SNPs and Genes previously reported to associate with BMD are marked with stars (*). Wald stats: z-score transformed from p values. | | | | | | | | |
