# Supplementary material for: Genetic Sharing with Cardiovascular Disease Risk Factors and Diabetes Reveals Novel Bone Mineral Density Loci
Source: PLoS One. 2015 Dec 22;10(12):e0144531. doi: 10.1371/journal.pone.0144531 (PMC4687843; doi:10.1371/journal.pone.0144531)
Supplement: S5 Table — (DOCX) [file pone.0144531.s013.docx]

| **S5 Table. Gene titles and gene ontology function terms of genes associated with LS an FN BMD loci**  **at FDR <0.01** | | |
| --- | --- | --- |
| **Gene Symbol** | **Gene Title** | **GO molecular function term** |
| *AAAS* | achalasia, adrenocortical insufficiency, alacrimia | --- |
| *AAGAB* | alpha- and gamma-adaptin binding protein |  |
| *ABCF2* | ATP-binding cassette, sub-family F (GCN20), member 2 | nucleotide binding; transporter activity;  ATP binding; ATPase activity |
| *ADCY6* | adenylate cyclase 6 | receptor binding; protein binding; ATP binding; calcium- and calmodulin-responsive adenylate cyclase activity;phosphorus-oxygen lyase activity; protein kinase binding; metal ion binding |
| *AKAP11* | A kinase (PRKA) anchor protein 11 | protein phosphatase 1 binding ; protein complex scaffold; protein kinase A catalytic subunit binding;  protein kinase A regulatory subunit binding |
| *AMBRA1* | autophagy/beclin-1 regulator 1 | --- |
| *ANAPC1* | anaphase promoting complex subunit 1 | --- |
| *APEH* | acylaminoacyl-peptide hydrolase |  |
| *AREG* | Amphiregulin | cytokine activity; growth factor activity |
| *ARHGAP1* | Rho GTPase activating protein 1 | SH3/SH2 adaptor activity; Rho GTPase activator activity;  protein binding; GTP binding; SH3 domain binding; Rac GTPase activator activity |
| *ASB16* | ankyrin repeat and SOCS box containing 16 | protein binding |
| *ASB16-AS1* | ASB16 antisense RNA 1 | --- |
| *ATF7* | activating transcription factor 7 | sequence-specific DNA binding transcription factor activity; transcription factor binding; zinc ion binding; protein dimerization activity; mitogen-activated protein kinase binding |
| *ATP11B* | ATPase, class VI, type 11B |  |
| *AXIN1* | axin 1 | p53 binding; signal transducer activity; GTPase activator activity; beta-catenin binding; protein C-terminus binding; protein kinase binding; protein domain specific binding; ubiquitin protein ligase binding; protein complex scaffold; protein homodimerization activity; SMAD binding; armadillo repeat domain binding; I-SMAD binding; R-SMAD binding |
| *BDNF-AS* | brain-derived neurotrophic factor antisense RNA |  |
| *BET1L* | blocked early in transport 1 homolog (S. cerevisiae)-like | SNAP receptor activity |
| *BMP7* | bone morphogenetic protein 7 | cytokine activity; protein binding; growth factor activity |
| *BSN* | bassoon (presynaptic cytomatrix protein) | metal ion binding |
| *BSN-AS2* | BSN antisense RNA 2 |  |
| *C12orf23* | chromosome 12 open reading frame 23 | --- |
| *C17orf53* | chromosome 17 open reading frame 53 | --- |
| *C2orf73* | chromosome 2 open reading frame 73 | --- |
| *C7orf76* | chromosome 7 open reading frame 76 | --- |
| *CAPZA1* | Capping protein (actin filament) muscle Z-line, alpha 1 | actin binding |
| *CBR3-AS1* |  |  |
| *CCDC170* | coiled-coil domain containing 170 | --- |
| *CDK15* | cyclin-dependent kinase 15 | nucleotide binding; protein kinase activity; protein serine/threonine kinase activity; cyclin-dependent protein kinase activity; ATP binding ; kinase activity; transferase activity; transferase activity, transferring phosphorus-containing groups; metal ion binding |
| *CDKAL1* | CDK5 regulatory subunit associated protein 1-like 1 | catalytic activity; transferase activity; metal ion binding; 4 iron, 4 sulfur cluster binding |
| *CENPW* | centromere protein W |  |
| *CEP112* | centrosomal protein 112kDa |  |
| *COL11A1* | collagen, type XI, alpha 1 | extracellular matrix structural constituent; protein binding, bridging |
| *COLEC10* | collectin sub-family member 10 (C-type lectin) | mannose binding ; carbohydrate binding |
| *CPED1* | cadherin-like and PC-esterase domain containing 1 | --- |
| *CTNNB1* | catenin (cadherin-associated protein), beta 1, 88kDa | chromatin binding; sequence-specific DNA binding transcription factor activity; transcription coactivator activity; signal transducer activity; structural molecule activity; protein binding; protein C-terminus binding ;transcription factor binding; enzyme binding; kinase binding ; protein kinase binding; protein phosphatase binding ;estrogen receptor binding; ionotropic glutamate receptor binding; RPTP-like protein binding ; ion channel binding; alpha-catenin binding; cadherin binding ; SMAD binding; androgen receptor binding ;  I-SMAD binding; R-SMAD binding |
| *CYLD* | cylindromatosis (turban tumor syndrome) | ubiquitin thiolesterase activity; cysteine-type peptidase activity; zinc ion binding; protein kinase binding; proline-rich region binding |
| *CYP19A1* | cytochrome P450, family 19, subfamily A, polypeptide 1 | monooxygenase activity; iron ion binding; electron carrier activity; oxidoreductase activity, acting on paired donors, with incorporation or reduction of molecular oxygen, reduced flavin or flavoprotein as one donor, and incorporation of one atom of oxygen; oxidoreductase activity, acting on paired donors, with incorporation or reduction of molecular oxygen, reduced flavin or flavoprotein as one donor, and incorporation of one atom of oxygen; oxygen binding; heme binding ; aromatase activity |
| *DCDC5* | doublecortin domain containing 5 | --- |
| *DDN* | dendrin | protein binding |
| *DDR2* | Discoidin domain receptor tyrosine kinase 2 | transmembrane receptor protein tyrosine kinase activity; collagen binding; ATP binding |
| *DGKH* | diacylglycerol kinase, eta |  |
| *DHH* | desert hedgehog | patched binding; calcium ion binding; protein binding; peptidase activity; zinc ion binding; hydrolase activity |
| *DNM3* | dynamin 3 | GTPase activity; protein binding; GTP binding; phospholipid binding; hydrolase activity |
| *DSPP* | dentin sialophosphoprotei | extracellular matrix structural constituent; calcium ion binding; collagen binding |
| *EPDR1* | ependymin related protein 1 (zebrafish) | calcium ion binding |
| *ERCC1* | excision repair cross-complementing rodent repair deficiency, complementation group 1 (includes overlapping antisense sequence) | single-stranded DNA specific endodeoxyribonuclease activity; DNA binding; damaged DNA binding; protein domain specific binding; structure-specific DNA binding |
| *ESR1* | estrogen receptor 1 | chromatin binding; sequence-specific DNA binding transcription factor activity; steroid hormone receptor activity; ligand-activated sequence-specific DNA binding RNA polymerase II transcription factor activity; steroid binding; beta-catenin binding; zinc ion binding; lipid binding; enzyme binding; nitric-oxide synthase regulator activity; estrogen receptor activity; type 1 metabotropic glutamate receptor binding; protein complex binding; estrogen response element binding; estrogen-activated sequence-specific DNA binding RNA polymerase II transcription factor activity; hormone binding; identical protein binding |
| *EYA1* | eyes absent homolog 1 (Drosophila) | phosphoprotein phosphatase activity; protein tyrosine phosphatase activity; protein binding; metal ion binding |
| *FAM20C* | Family with sequence similarity 20, member C |  |
| *FAM210A* | family with sequence similarity 210, member A | --- |
| *FAM3C* | family with sequence similarity 3, member C | cytokine activity |
| *FUBP3* | far upstream element (FUSE) binding protein 3 | DNA binding; RNA binding |
| *GAL* | galanin prepropeptide | neuropeptide hormone activity |
| *GALNT3* | UDP-N-acetyl-alpha-D-galactosamine:polypeptide N-acetylgalactosaminyltransferase 3 (GalNAc-T3) | polypeptide N-acetylgalactosaminyltransferase activity; calcium ion binding; transferase activity, transferring glycosyl groups; manganese ion binding; carbohydrate binding |
| *GNAZ* | guanine nucleotide binding protein (G protein), alpha z polypeptide |  |
| *GNG12-AS1* | GNG12 antisense RNA 1 |  |
| *GPATCH1* | G patch domain containing 1 | nucleic acid binding |
| *GRB10* | growth factor receptor-bound protein 10 | SH3/SH2 adaptor activity; insulin receptor binding; phospholipid binding |
| *HARBI1* | harbinger transposase derived 1 |  |
| *HOXC4* | homeobox C4 | sequence-specific DNA binding transcription factor activity; transcription corepressor activity; HMG box domain binding |
| *HOXC5* | homeobox C5 |  |
| *HOXC6* | homeobox C6 |  |
| *IBSP* | integrin-binding sialoprotein | --- |
| *IDUA* | Iduronidase, alpha-L- | L-iduronidase activity; hydrolase activity, hydrolyzing O-glycosyl compounds; cation binding |
| *IFT172* | intraflagellar transport 172 homolog (Chlamydomonas) | --- |
| *INSIG2* | insulin induced gene 2 | transcription factor binding |
| *JAG1* | Jagged 1 | Notch binding ; structural molecule activity; calcium ion binding ; growth factor activity |
| *KANSL1* | KAT8 regulatory NSL complex subunit 1 | protein binding; histone acetyltransferase activity (H4-K5 specific); histone acetyltransferase activity (H4-K8 specific); histone acetyltransferase activity (H4-K16 specific) |
| *KANSL1-AS1* | KANSL1 antisense RNA 1 |  |
| *KAT5* | K(lysine) acetyltransferase 5 | transcription coactivator activity; histone acetyltransferase activity; protein binding;metal ion binding; androgen receptor binding; repressing transcription factor binding |
| *KCNMA1* | potassium large conductance calcium-activated channel, subfamily M, alpha member 1 | nucleotide binding; actin binding; voltage-gated potassium channel activity; large conductance calcium-activated potassium channel activity |
| *KIAA1468* | KIAA1468 | --- |
| *KIAA2018* | KIAA2018 | DNA binding; mannosyl-oligosaccharide 1,2-alpha-mannosidase activity; calcium ion binding |
| *KIF2B* | kinesin family member 2B | microtubule motor activity; ATP binding |
| *KLF4* | Kruppel-like factor 4 (gut) | RNA polymerase II core promoter proximal region sequence-specific DNA binding transcription factor activity involved in positive regulation of transcription; zinc ion binding; phosphatidylinositol 3-kinase regulator activity; sequence-specific DNA binding |
| *KLF6* | Kruppel-like factor 6 |  |
| *KLHL42* | kelch-like family member 42 | --- |
| *LACTB2* | lactamase, beta 2 | hydrolase activity; metal ion binding |
| *LEKR1* | leucine, glutamate and lysine rich 1 | --- |
| *LGR4* | Leucine-rich repeat containing G protein-coupled receptor 4 | signal transducer activity; G-protein coupled receptor activity; protein-hormone receptor activity |
| *LINC00942* | long intergenic non-protein coding RNA 942 |  |
| *LRP4* | low density lipoprotein receptor-related protein 4 |  |
| *LRP4-AS1* | LRP4 antisense RNA 1 |  |
| *LRP5* | Low density lipoprotein receptor-related protein 5 | receptor activity; protein binding; coreceptor activity; Wnt-protein binding; toxin transporter activity; Wnt-activated receptor activity |
| *LUC7L* | LUC7-like (S. cerevisiae) |  |
| *MACF1* | microtubule-actin crosslinking factor 1 | actin binding; calcium ion binding;microtubule binding;ATPase activity |
| *MALAT1* | metastasis associated lung adenocarcinoma transcript 1 (non-protein coding) |  |
| *MARK3* | MAP/microtubule affinity-regulating kinase 3 | nucleotide binding; protein serine/threonine kinase activity; protein binding; ATP binding; kinase activity; transferase activity, transferring phosphorus-containing groups |
| *MBL2* | mannose-binding lectin (protein C) 2, soluble | receptor binding; protein binding; mannose binding; eukaryotic cell surface binding; calcium-dependent protein binding; bacterial cell surface binding |
| *MEF2C* | myocyte enhancer factor 2C | sequence-specific DNA binding RNA polymerase II transcription factor activity; histone deacetylase; protein heterodimerization activity, HMG box domain binding |
| *MEF2D* | myocyte enhancer factor 2D | sequence-specific DNA binding RNA polymerase II transcription factor activity; enzyme binding; activating transcription factor binding; protein homodimerization activity; histone deacetylase binding; protein heterodimerization activity |
| *MEPE* | matrix extracellular phosphoglycoprotein | extracellular matrix structural constituent ; protein binding |
| *METTL21A* | methyltransferase like 21A | methyltransferase activity |
| *MIR1208* | microRNA 1208 |  |
| *MIR4713* | microRNA 4713 |  |
| *MIR3120* | microRNA 3120 |  |
| *MIR1262* | microRNA 1262 |  |
| *MIR595* | microRNA 595 |  |
| *MIR4701* | microRNA 4701 |  |
| *MIR196A2* | microRNA 196A2 | --- |
| *MLXIP* | MLX interacting protein | DNA binding |
| *MPP7* | membrane protein, palmitoylated 7 (MAGUK p55 subfamily member 7) | protein domain specific binding; protein complex scaffold; signaling adaptor activity; protein heterodimerization activity |
| *NAB1* | NGFI-A binding protein 1 (EGR1 binding protein 1) | transcription factor binding |
| *NFATC1* | nuclear factor of activated T-cells, cytoplasmic, calcineurin-dependent 1 | RNA polymerase II transcription factor binding; RNA polymerase II distal enhancer sequence-specific DNA binding transcription factor activity involved in positive regulation of transcription; FK506 binding; mitogen-activated protein kinase p38 binding |
| *NME8* | NME/NM23 nucleoside diphosphate kinase 8 | nucleoside diphosphate kinase activity; ATP binding; kinase activity; transferase activity; metal ion binding |
| *NSF* | vesicle-fusing ATPase-like; N-ethylmaleimide-sensitive factor | protein binding; ATP binding; protein C-terminus binding; ATPase activity; nucleoside-triphosphatase activity; Rab GTPase binding; syntaxin binding; PDZ domain binding; protein complex binding; metal ion binding |
| *NTAN1* | N-terminal asparagine amidase |  |
| *PDXDC1* | pyridoxal-dependent decarboxylase domain containing 1 | carboxy-lyase activity; pyridoxal phosphate binding |
| *PKDCC* | protein kinase domain containing, cytoplasmic |  |
| *PIGN* | phosphatidylinositol glycan anchor biosynthesis, class N | catalytic activity; transferase activity |
| *PKIA* | protein kinase (cAMP-dependent, catalytic) inhibitor alpha | cAMP-dependent protein kinase inhibitor activity |
| *PKDCC* | protein kinase domain containing, cytoplasmic | --- |
| *PPP1CB* | Protein phosphatase 1, catalytic subunit, beta isozyme | protein serine/threonine phosphatase activity; protein binding; protein kinase binding; metal ion binding ; myosin-light-chain-phosphatase activity |
| *PPP6R3* | protein phosphatase 6, regulatory subunit 3 | protein phosphatase binding |
| *PTPRD* | protein tyrosine phosphatase, receptor type, D | protein tyrosine phosphatase activity; transmembrane receptor protein tyrosine phosphatase activity; receptor binding; cell adhesion molecule binding |
| *PTPRN2* | protein tyrosine phosphatase, receptor type, N polypeptide 2 | phosphoprotein phosphatase activity; receptor activity;  transmembrane receptor protein tyrosine phosphatase activity |
| *PTX4* | pentraxin 4, long | [metal ion binding](http://amigo.geneontology.org/cgi-bin/amigo/term_details?term=GO:0046872&session_id=8873amigo1366622851&) |
| *RAB36* | RAB36, member RAS oncogene family |  |
| *RAB9BP1* | RAB9B, member RAS oncogene family pseudogene 1 | --- |
| *RELA* | V-rel reticuloendotheliosis viral oncogene homolog A (avian) | chromatin binding; RNA polymerase II distal enhancer sequence-specific DNA binding transcription factor activity;ankyrin repeat binding |
| *RERE* | arginine-glutamic acid dipeptide (RE) repeats | sequence-specific DNA binding transcription factor activity; protein binding; poly-glutamine tract binding; zinc ion binding |
| *RHEBL1* | Ras homolog enriched in brain like 1 |  |
| *RHPN2* | rhophilin, Rho GTPase binding protein 2 | --- |
| *RIC8B* | resistance to inhibitors of cholinesterase 8 homolog B (C. elegans) | G-protein alpha-subunit binding; guanyl-nucleotide exchange factor activity |
| *RPS6KA5* | ribosomal protein S6 kinase, 90kDa, polypeptide 5 | nucleotide binding; magnesium ion binding; protein serine/threonine kinase activity; protein binding; ATP binding; transferase activity, transferring phosphorus-containing groups |
| *RTDR1* | rhabdoid tumor deletion region gene 1 | --- |
| *SALL1* | sal-like 1 (Drosophila) | chromatin binding; sequence-specific DNA binding transcription factor activity; histone deacetylase activity; protein binding; beta-catenin binding; zinc ion binding |
| *SAMD12-AS* | sterile alpha motif domain containing 12 antisense RNA 1 |  |
| *SEMA3D* | sema domain, immunoglobulin domain (Ig), short basic domain, secreted, (semaphorin) 3D | receptor activity |
| *SFRP4* | secreted frizzled-related protein 4 | Wnt-protein binding; PDZ domain binding; Wnt-activated receptor activity |
| *SHFM1* | split hand/foot malformation (ectrodactyly) type 1 | protein binding; peptidase activity |
| *SLC1A3* | solute carrier family 1 (glial high affinity glutamate transporter), member 3 | high-affinity glutamate transmembrane transporter activity; glutamate binding;sodium:dicarboxylate symporter activity |
| *SLC25A16* | Solute carrier family 25 (mitochondrial carrier; Graves disease autoantigen), member 16 | solute:solute antiporter activity |
| *SLX4IP* | SLX4 interacting protein |  |
| *SMAD3* | SMAD family member 3 | core promoter proximal region sequence-specific DNA binding; protein binding transcription factor activity; RNA polymerase II activating transcription factor binding; chromatin binding; transforming growth factor beta receptor binding; protein binding; collagen binding; beta-catenin binding; zinc ion binding; protein kinase binding; phosphatase binding; transforming growth factor beta receptor, pathway-specific cytoplasmic mediator activity; chromatin DNA binding; ubiquitin protein ligase binding; protein homodimerization activity; ubiquitin binding; sequence-specific DNA binding; sequence-specific DNA binding; transcription regulatory region DNA binding; SMAD binding; metal ion binding; co-SMAD binding; R-SMAD binding |
| *SMARCD3* | SWI/SNF related, matrix associated, actin dependent regulator of chromatin, subfamily d, member 3 | receptor binding; transcription factor binding; ligand-dependent nuclear receptor transcription coactivator activity |
| *SMG6* | smg-6 homolog, nonsense mediated mRNA decay factor (C. elegans) | DNA binding; endoribonuclease activity; protein binding; telomeric DNA binding ; metal ion binding |
| *SNORD67* | small nucleolar RNA, C/D box 67 |  |
| *SNORD27* | small nucleolar RNA, C/D box 27 |  |
| *SOST* | Sclerostin | transcription factor binding; heparin binding |
| *SOX6* | SRY (sex determining region Y)-box 6 | sequence-specific DNA binding transcription factor activity; protein heterodimerization activity |
| *SP7* | Sp7 transcription factor | DNA binding; zinc ion binding; DEAD/H-box RNA helicase binding; DEAD/H-box RNA helicase binding |
| *SPP1* | secreted phosphoprotein 1 |  |
| *SPTBN1* | spectrin, beta, non-erythrocytic 1 | actin binding; structural constituent of cytoskeleton; protein binding; calmodulin binding; phospholipid binding; ankyrin binding |
| *STARD3NL* | STARD3 N-terminal like | --- |
| *SUPT3H* | suppressor of Ty 3 homolog (S. cerevisiae) | DNA binding; transcription coactivator activity; histone acetyltransferase activity |
| *SUSD4* | sushi domain containing 4 | --- |
| *SUSD5* | sushi domain containing 5 | hyaluronic acid binding |
| *TESK2* | testis-specific kinase 2 | nucleotide binding; protein serine/threonine/tyrosine kinase activity; protein tyrosine kinase activity; ATP binding; transferase activity, transferring phosphorus-containing groups; metal ion binding |
| *TMEM135* | transmembrane protein 135 | --- |
| *TMEM175* | transmembrane protein 175 | --- |
| *TMEM194B* | transmembrane protein 194B | --- |
| *TNFRSF11A* | tumor necrosis factor receptor superfamily, member 11a, NFKB activator | transmembrane signaling receptor activity; tumor necrosis factor-activated receptor activity; protein binding; cytokine binding; metal ion binding |
| *TNFRSF11B* | tumor necrosis factor receptor superfamily, member 11b | receptor activity; cytokine activity |
| *TNFSF11* | tumor necrosis factor (ligand) superfamily, member 11 | receptor activity; cytokine activity ; tumor necrosis factor receptor superfamily binding |
| *ULK4* | unc-51-like kinase 4 (C. elegans) | protein serine/threonine kinase activity; ATP binding; transferase activity, transferring phosphorus-containing groups |
| *WLS* | wntless homolog | [Wnt-protein binding](http://amigo.geneontology.org/cgi-bin/amigo/term_details?term=GO:0017147&session_id=6401amigo1366622685&) |
| *WNT16* | wingless-type MMTV integration site family, member 16 | receptor binding; frizzled binding |
| *WNT2B* | wingless-type MMTV integration site family, member 2B | receptor binding; frizzled-2 binding |
| *WNT4* | wingless-type MMTV integration site family, member 4 | G-protein coupled receptor binding; transcription corepressor activity; frizzled binding; extracellular matrix structural constituent; receptor agonist activity |
| *XKR9* | XK, Kell blood group complex subunit-related family, member 9 | --- |
| *ZBTB40* | zinc finger and BTB domain containing 40 | DNA binding; zinc ion binding |
| *ZFP42* | zinc finger protein 42 homolog (mouse) | sequence-specific DNA binding transcription factor activity; zinc ion binding |
| *ZNF652* | Zinc finger protein 652 | DNA binding; protein binding; zinc ion binding |
